# The risk factors of anti-neutrophil cytoplasmic antibody-associated vasculitis-associated interstitial lung disease: a systematic review and meta-analysis

**DOI:** 10.1016/j.clinsp.2025.100823

**Published:** 2025-11-05

**Authors:** Ningxia Yu, Shuguang Yang, Danyang Zang, Lili Xu, Ruonan Yan, Xueqing Yu

**Affiliations:** a National Regional Traditional Chinese Medicine (Lung Disease) Diagnosis and Treatment Center, the First Affiliated Hospital of Henan University of Chinese Medicine, China; b The First Clinical Medical College, Henan University of Chinese Medicine, China

**Keywords:** ANCA-Associated Vasculitis, Interstitial lung disease, Risk Factors, Systematic Review, Meta-Analysis

## Abstract

**Background:**

Anti-neutrophil cytoplasmic Antibody-Associated Vasculitis (AAV) is an immune-mediated disease characterized by Anti-Neutrophil Cytoplasmic Antibody (ANCA) positivity. Patients with AAV are known to be at high risk for the occurrence of Interstitial Lung Disease (ILD). The authors aimed to explore the risk factors in patients with AAV-ILD to enhance awareness among the public and healthcare providers, and to facilitate the timely adoption of effective strategies for prevention and management.

**Methods:**

The authors searched PubMed, Embase, Cochrane Library, China National Knowledge Infrastructure Database (CNKI), the WanFang Database, Chinese biomedical literature service system (SinoMed), and China Science and Technology Journal Database (VIP) for studies that focused on the risk factors in patients with AAV-ILD from database inception to 21 September 2024. Methodological quality was assessed using the Newcastle-Ottawa Scale (NOS). The authors used a random-effects model or a fixed-effects model to evaluate the underlying risk factors of AAV-ILD.

**Results:**

A total of 25 studies were included, including 13 case-control studies, 5 cohort studies, and 7 cross-sectional studies, respectively. The authors identified preliminary risk factors for AAV-ILD, including age, male, smoking history, KL-6, ESR, MPO-ANCA, cough, dyspnea, and HRCT findings such as honeycombing, interlobular septal thickening, and lattice shadows, Ear, nose & throat involvement, Hb, alb, BVAS, and fever. The study design type, follow-up (time), Country, sample, type of AAV, quality score, and were initially considered not to be sources of heterogeneity.

**Conclusions:**

This study has preliminarily identified multiple risk factors associated with ILD in AAV patients, encompassing sociodemographic, lifestyle, and clinical factors. While these findings may serve as a reference for further investigation, large-scale prospective cohort studies or longitudinal research remain necessary for validation.

**Systematic review registration:**

PROSPERO, identifier CRD42024592874.

## Highlights

• Identifies key risk factors for AAV-ILD, encompassing sociodemographic, lifestyle, and clinical factors.
• Older age, male, and smoking history are strong predictors of AAV-ILD development.
• Clinical factors, including but not limited to KL-6, ESR, and MPO-ANCA, are risk factors for AAV-ILD.
• Findings contribute to early warning and risk stratification for AAV-ILD patients.

## Introduction

AAV is a rare but serious autoimmune disease characterized by inflammation and damage to small blood vessels, leading to multi-organ involvement and a poor prognosis. AAV imposes a massive socioeconomic burden on the global healthcare system. 1, 2, 3, 4 AAV is a group of necrotizing vasculitis that includes Granulomatosis with Polyangiitis (GPA), Microscopic Polyangiitis (MPA), and Eosinophilic Granulomatosis with Polyangiitis (EGPA). AAV is characterized by the presence of ANCA, which is typically specific for Myeloperoxidase (MPO) or Proteinase-3 (PR3). These autoantibodies play a crucial role in the pathogenesis and diagnosis of AAV. ^5^ Pulmonary involvement is a common complication in patients with AAV, manifesting as diverse patterns such as ILD. ^6^ A retrospective study^7^ showed that ILD accounted for the highest proportion of AAV cases with lung involvement, at 55.7 %. In recent years, the link between ILD and ANCA or AAV has been increasingly recognized, with studies suggesting that ILD may precede or coexist with AAV, significantly impacting disease progression and patient outcomes. ^8^ However, our understanding of the factors associated with AAV-ILD remains sparse, particularly regarding the role of sociodemographic, lifestyle, and Clinical factors in disease pathogenesis and progression.

Studies have reported that the prevalence rate of AAV ranges from 300‒421 per million persons. ^9^ AAV-ILD is identified in up to 45 % of patients with MPA and 23 % of GPA, with a particularly high prevalence observed in the Asian population. ^6^ In the majority of patients, the onset of ILD occurs concurrently with or prior to the AAV. Previous studies have reported that ILD precedes AAV in 14 %‒85 % of patients, coincides with ILD in 36 %‒67 % of patients, and follows AAV in 8 %‒21 % of patients. ^7^ However, once clinical signs of ILD become apparent, it leads to considerable mortality and poor pulmonary function, significantly impacting patient outcomes. ^10^ Thus, further understanding of the factors associated with AAV-ILD may help elucidate the pathogenesis of AAV in certain patients and provide a scientific basis for the early diagnosis, risk stratification, and personalized management of AAV patients with ILD.

According to published studies, the prevalence of AAV-ILD varies significantly from 10 % to 60 %, with higher prevalence associated with increased mortality. 11, 12, 13, 14, 15 This wide range may be attributed to differences in diagnostic criteria, study populations, and geographic regions. To date, numerous studies have identified several variables as significant predictors of AAV-ILD, including advanced age, male sex, elevated KL-6 levels, and a history of smoking. ^11^^,^^16^, ^17^, ^18^ Nevertheless, the results are discordant, with some factors, such as the use of plasma exchange, remaining controversial due to inconsistent findings across studies. ^11^^,^^19^ Moreover, no study has systematically summarized these potential risk factors or quantitatively evaluated their association with AAV-ILD. Therefore, this meta-analysis aims to comprehensively assess and quantify the risk factors for AAV-ILD, providing robust evidence for early identification and targeted interventions.

## Materials and methods

A systematic literature review and meta-analysis were conducted in accordance with the guidelines of the Preferred Reporting Items for systematic reviews and meta-analyses (PRISMA). ^20^ All steps of this systematic review and meta-analysis were performed according to protocol and prospectively registered in the International Prospective Registry of Systematic Reviews database under the number CRD42024592874.

### Search strategy

Two members of our group (Ningxia Yu and Lili Xu) searched the PubMed, Embase, Cochrane Library, China National Knowledge Infrastructure Database (CNKI), the WanFang Database, Chinese biomedical literature service system (SinoMed), and China Science and Technology Journal Database (VIP) for studies that were published from database inception to 21 September 2024, with a systemic literature search strategy that included Supplement A and B. The retrieval strategy involved using the following subject terms, which included ‘ANCA-associated vasculitis’, ‘interstitial lung disease’, and ‘risk factor’.

### Inclusion and exclusion criteria

The inclusion criteria for this study were as follows: 1) Case-control, cross-sectional, and prospective or retrospective cohort studies with AAV-ILD population; 2) Studies related to the risk factors of AAV-ILD; 3) AAV-ILD patients(AAV without ILD served as the control group); 4) Only studies written in English were included; 5) Only one study was included if the same data was published in different journals; 6) If duplicate studies were found, the study with the largest sample size or more detailed information was selected. The exclusion criteria were as follows: 1) Subclinical ILD, patients with ILD before or meantime to AAV onset were excluded; 2) Letters, reviews, commentaries, abstracts, and editorials were excluded; 3) Duplicated papers written by the same authors; 4) Studies for which full text was not available; 5) Studies focusing on animals only.

### Data extraction and risk factor assessments

The two members of our group (Ningxia Yu and Ruonan Yan) carried out data extraction independently. Discrepancies were resolved by discussion with a third author. With reference to the inclusion and exclusion criteria, the results of the study search were categorized based on EndNote X9 software and duplicate publications were removed. Then, the studies were screened by reading the titles and abstracts, and finally, the full texts were read to select the studies that fulfilled the requirements. The data of the selected articles, including the name of the first author, Country, year of publication, sample size, study design, duration of follow-up, clinical, demographic features (age, gender, smoking history, diagnosis, medication used, etc.), and quality assessment of the included studies.

### Risk-of-bias and certainty-of-evidence assessment

Two reviewers (Ningxia Yu and Ruonan Yan) separately assessed the quality of all selected studies based on the Newcastle-Ottawa Quality Assessment Scale (NOS). ^21^ Briefly, three categories of study group selection, comparability of study groups, and determination of relevant outcomes comprise the NOS. Each study was assigned a score of 0‒9, with a final score of ≥7 considered high quality. Disagreements were settled by a senior researcher (Danyang Zang). The certainty of evidence for each outcome was assessed using the Grading of Recommendations Assessment, Development, and Evaluation (GRADE) system, which categorizes evidence into 4 levels: very low, low, moderate, and high. ^22^ The quality of evidence from observational studies was initially categorized as “low” and then upgraded or downgraded according to predetermined criteria. The Agency for Healthcare Research and Quality (AHRQ) was used to evaluate the quality of cross-sectional studies. ^23^ Based on AHRQ scores of 0‒3, 4‒7 and 8‒11, studies were categorized as low, moderate or high quality.

### Data synthesis and analysis

Data extraction for the meta-analysis was performed using Stata statistical software version 18.0 and *R* statistical language version 4.4.1. A two-sided *p*-value < 0.05 was considered significant. The calculated results of the pooled RR with 95 % CI were used as effect sizes to summarize the relationship. Heterogeneity between studies was quantified by the *I*^2^ statistic. Random effects models were used for substantial or high heterogeneity, and fixed effects models (Mantel-Haenszel method) were used for low or moderate heterogeneity. ^24^ To assess potential sources of the heterogeneity and subgroup disparities, subgroup analyses were conducted according to follow-up (time), study design type, sample, and Country. Meanwhile, the authors conducted meta-regressions to evaluate subgroup differences and to further examine whether the estimates were influenced by study-level covariates. Differences between the estimates for these subgroups were considered significant when *p* < 0.1. To assess the robustness of the pooled results, the authors conducted a sensitivity analysis using the “leave-one-out” method. Egger's test and Begg's test were used to visualize the funnel plots to assess potential publication bias.

## Results

### Study selection and characteristics

**Fig. 1:**
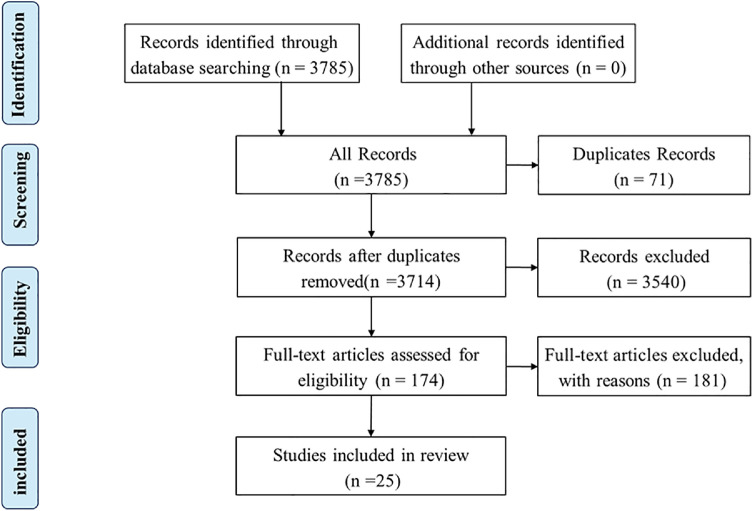
Flow diagram of study selection.

In the preliminary analysis, the search strategy identified 3785 studies. After removing duplicates, 3714 studies were screened based on titles and abstracts. Then resulting in 174 studies were selected for full-text review. Eventually, a total of 25 studies fulfilled the selection criteria and were included in our review. The flow diagram (Fig. 1) showed the process of literature selection and identification.

A total of 25 studies were included in this study, ^11^^,^^12^^,^^16^, ^17^, ^18^, ^19^^,^^25^, ^26^, ^27^, ^28^, ^29^, ^30^, ^31^, ^32^, ^33^, ^34^, ^35^, ^36^, ^37^, ^38^, ^39^, ^40^, ^41^, ^42^, ^43^ including 13 case-control studies, ^11^^,^^12^^,^^17^^,^^25^, ^26^, ^27^, ^28^, ^29^^,^^31^^,^^33^^,^^36^^,^^40^^,^^42^ 5 cohort studies, ^16^^,^^34^^,^^35^^,^^38^^,^^41^ 7 cross-sectional studies. ^18^^,^^19^^,^^30^^,^^32^^,^^37^^,^^39^^,^^43^ The publication years of the studies included in our review ranged from 2001 to 2024. The studies were conducted in different countries, including America, China, Argentina, Japan, France, Greece, Italy and Mexico. Most patients with AAV-ILD included in our review were male or smokers. AAV and ILD were determined primarily on the basis of international classification codes.

The characteristics of the 25 studies in our review were summarized in Table 1. A total of 1404 AAV-ILD and 2074 AAV patients were included. Regarding study quality, case-control study and cohort study, there was high quality in 14 studies^11^^,^^12^^,^^16^^,^^17^^,^^25^, ^26^, ^27^^,^^31^^,^^33^^,^^34^^,^^36^^,^^38^^,^^40^ and moderate quality overall in the remaining 4 studies^28^^,^^35^^,^^41^^,^^41^ according to the NOS tool. There was high quality in 2 studies^37^^,^^43^ and moderate quality overall in the remaining 5 studies, ^18^^,^^19^^,^^30^^,^^32^^,^^39^ with the median score of 6 according to the AHRQ tool. Detailed assessment of study quality for each domain of included studies is shown in Supplement C.

**Table 1:** Characteristics of the included studies.

Author, ref	Country	Study design	AAV types	Sample (AAV-ILD/AAV)	Gender (M/F)		Age (year)		Smoking		Follow-up (time/mo)		Significant risk factor
					AAV-ILD	AAV	AAV-ILD	AAV	AAV-ILD	AAV	AAV-ILD	AAV	
Maillet^11^	France	Case-control study	AAV	62/124	34/28	54/70	66 (56‒74)	67 (56‒73)	36	24	40.5 (21‒68)	66.1 (26‒128)	3,8,9,10,11
Tzelepis^12^	Greece	Case-control study	MPA	13/20	9/4	7/13	57	60	N/R	N/R	38±30	38±30	8,12,13,14,15,16
Yang^16^	China	Retrospective cohort study	AAV	101/68	58/43	27/41	70.2 ± 9.8	65.2 ± 10.3	57	23	22.7 (2.8‒51.2)	30.3 (13.1‒62.9)	1,2,3,6,17
Hozumi^17^	Japan	Case-control study	MPA	84/95	56/28	34/61	73.8 ± 8.7	72.6 ± 12.9	57	30	43.9 ± 40.1	57.1 ± 58.2	1,3
Iwata^18^	Japan	Cross-sectional study	AAV	6/7	3/3	5/2	69.83±11.37	70.29±5.22	N/R	N/R	N/R	N/R	4
Matsuda^19^	Japan	Cross-sectional study	MPA	32/17	17/15	8/9	76 (71‒82)	71 (68‒77)	18	11	N/R	N/R	2,4,11,18
Jiang^25^	China	Case-control study	AAV	72/32	25/47	14/18	64±12	49±13	N/R	N/R	N/R	N/R	2,7,17,18,19
Miao^26^	China	Case-control study	AAV	48/36	20/28	9/27	55.65±14.14	36.19±14.13	7	4	N/R	N/R	2,6,7,19,20,23
Wu^27^	China	Case-control study	AAV	15/36	6/9	14/22	72 (66, 79)^a^	65 (58, 71)^a^	4	6	37 (10, 56)	37 (10, 56)	2,5,14,15,16
Zhang^28^	China	Case-control study	AAV	93/52	54/39	32/20	63.90±13.5	67.10±11.9	22	13	N/R	N/R	NR
Xie^29^	China	Case-control study	AAV	109/247	51/58	95/152	66.00±10.21	57.50±15.03	21	35	31 (19, 56)	31 (19, 56)	2,9,19,20
Tan^30^	China	Cross-sectional study	AAV	45/42	29/16	21/21	63.29±14.37	58.12±15.74	N/R	N/R	N/R	N/R	4,6
Wang^31^	China	Case-control study	AAV	19/27	15/4	18/9	70 (63, 74)^a^	64 (58, 71)^a^	N/R	N/R	17 (2, 69)	17 (2, 69)	13,20
Liu^32^	China	Cross-sectional study	AAV	20/23	10/10	9/14	57.55±14.08	50.90±16.39	N/R	N/R	N/R	N/R	4
Jiang^33^	China	Case-control study	AAV	72/32	25/47	14/18	64±12	49±13	N/R	N/R	N/R	N/R	2,7,10,18,19,20,21,23
Matsuda^34^	Japan	Retrospective cohort study	MPA	115/94	52/63	42/52	74 (69‒79)	72 (65‒78)	N/R	N/R	N/R	N/R	4,17,18,24,25
Suzuki^35^	Japan	Prospective cohort study	MPA	74/70	42/32	24/46	73.4 ± 7.5	68.8 ± 13.4	31	22	N/R	N/R	1,4,14,15,16
Fernandez Casares^36^	Argentina	Case-control study	AAV	9/19	5/4	7/12	60±14	55±18	7	5	76±60	67±39	3,26
Conticini^37^	Italy	Cross-sectional study	GPA, MPA	4/8	2/2	2/6	73.73±8.12	52.75±11.90	N/R	N/R	N/R	N/R	4
Doliner^38^	America	Retrospective cohort study	AAV	91/593	42/49	238/355	67±12	58±18	N/R	N/R	N/R	N/R	2,17,19
Matsuda^39^	Japan	Cross-sectional study	MPA	32/17	17/15	8/9	76 (71‒82)	71 (68‒77)	N/R	N/R	N/R	N/R	2,4,18,24
Matsuda^40^	Japan	Case-control study	MPA	47/33	23/24	15/18	76 (70‒80)	71 (67‒78)	27	17	N/R	N/R	2,4,17,18
Sada^41^	Japan	Cohort study	AAV	61/95	28/33	33/62	69.3 ± 1.6	67.3 ± 1.3	N/R	N/R	N/R	N/R	9,17,19
Namba^42^	Japan	Case-control study	AAV	163/264	74/89	102/162	71.5 ± 10.2	64.8 ± 13.7	N/R	N/R	N/R	N/R	27
Flores-Suárez^43^	Mexico	Cross-sectional study	MPA	17/23	9/8	5/18	54.2	54.2	N/R	N/R	N/R	N/R	1,7,10,12,22,25,26

Note: 1. Sex (male); 2. Age; 3. Smoking; 4. KL-6; 5. Pulmonary comorbidity; 6. CRP; 7. ESR; 8. Fever; 9. Ear, nose & throat; 10. Peripheral neuropathy; 11. Plasma exchange; 12. Myalgias; 13. Dyspnoea; 14. Reticular pattern; 15. Interlobular septal thickening; 16. Honeycombing; 17. BVAS; 18. Cr; 19. MPO-ANCN; 20. Cough; 21. Dyspnea; 22. Haematuria; 23. Rash; 24. Alb; 25. Hb; 26. Diffuse alveolar haemorrhage; 27. rs35705950T; N/R. Not Report. ^a^ Mean(P25, P75).

### Risk factors associated with AAV-ILD

Twenty-five studies elaborated on the risk factors and measured effects. ^11^^,^^12^^,^^16^, ^17^, ^18^, ^19^^,^^25^, ^26^, ^27^, ^28^, ^29^, ^30^, ^31^, ^32^, ^33^, ^34^, ^35^, ^36^, ^37^, ^38^, ^39^, ^40^, ^41^, ^42^, ^43^ Data on these variables could only be pooled if they were collected when AAV-ILD was first diagnosed. Risk factors were divided into three categories: sociodemographic factors included age, female, and male; lifestyle factors included smoking history; and clinical factors included, having cardiovascular damage, cardiac damage, honeycombing, lattice shadows, interlobular septal thickening, positive MPO-ANCA, lower Erythrocyte Sedimentation Rate (ESR), higher Krebs von den Lungen-6 (KL-6), higher Hemoglobin (Hb), moderate and high Birmingham Vasculitis Activity Score (BVAS), Ear, nose & throat, dyspnea, cough, peripheral neuropathy, albumin (alb), pulmonary comorbidities, Positive Proteinase 3 (PR3)-ANCA, use of plasma exchange, myalgias, Diffuse Alveolar Haemorrhage (DAH), rash, fever, C-Reactive Protein (CRP), Serum Creatinine (SCr), haematuria, bloody sputum, shortness of breath, nasal crusting, acute respiratory failure, disease duration, chest pain, Granulocyte macrophage-colony stimulating factor (GM-CSF), Interleukin-6 (IL-6), Interleukin-2 (IL-2), CCL-2, Interleukin-17 (IL-17), Carcinoembryonic Antigen (CEA), Carbohydrate Antigen 199 (CA199), Carbohydrate Antigen 125 (CA125), pulmonary diffusion dysfunction, central neuropathy, eye involvement, head and neck involvement, thyroid gland involvement, the percentage of Five Factor Score (FFS) ≥ 3, follow-up (times), cutaneous, gastrointestinal, pulmonary nodules, mononeuropathy multiplex, immunosuppressive agent, cyclophosphamide, rituximab, methotrexate (Methotrexate), mycophenolate mofetil (CellCept), general symptom involvement, renal involvement, Ground-Glass Opacity (GGO), rs35705950T, respiratory symptoms, Forced Vital Capacity (FVC) ( %), Carbon monoxide Diffusing Capacity (DLCO) ( %), Total Lung Capacity (TLC), traction bronchiectasis, pleural effusion, emphysema, bronchiectasis, micronodules, centrilobular nodules and HRCT score. Table 2 summarizes the pooled results of all the factors related to AAV-ILD. All forest plots with risk factors in RA-ILD are shown in Supplement D (Fig. 2‒27).

**Table 2:** Meta-analysis of risk factors for AAV-ILD.

Outcomes	Number of trials	Effect size	95 %CI	*p*-value	Heterogeneity	
					*I*^2^	*p*-value
Sociodemographic						
Male	3	1.65^O^^Rs^	1.20‒2.28	<0.05	0.0 %	0.806
Age	10	8.76 ^WMDs^	5.95‒11.57	<0.05	79.0 %	<0.1
Lifestyle						
Having smoking history	4	2.21^O^^Rs^	1.61‒3.04	<0.05	0.0 %	0.552
Clinical						
KL-6	8	400.05 ^WMDs^	285.20‒514.91	<0.05	87.8 %	<0.1
Pulmonary comorbidities	3	0.98^O^^Rs^	0.30‒3.21	0.973	75.5 %	<0.1
PR3-ANCA	3	0.66^O^^Rs^	0.13‒3.22	0.606	76.1 %	<0.1
Plasma exchange	2	1.71^O^^Rs^	0.01‒299.82	0.838	87.3 %	<0.1
Myalgias	2	1.80^O^^Rs^	0.09‒34.39	0.695	79.8 %	<0.1
Hb	2	0.91^O^^Rs^	0.37‒1.45	<0.05	0.0 %	0.381
ESR	3	21.83 ^WMDs^	7.67‒36.00	<0.05	81.7 %	<0.1
Alveolar haemorrhage	2	0.71 ^ORs^	0.02‒24.25	0.825	80.5 %	<0.1
CRP	3	5.51 ^WMDs^	−20‒31.01	0.672	88.7 %	<0.1
Cr	4	−0.65 ^WMDs^	−1.71‒0.41	0.229	95.8 %	<0.1
BVAS	5	−2.83 ^WMDs^	−4.20‒−1.45	<0.05	93.2 %	<0.1
Alb	2	0.22 ^WMDs^	0.04‒0.41	<0.05	0.0 %	0.756
Honeycombing	3	59.53 ^ORs^	8.42‒420.92	<0.05	0.0 %	0.672
Interlobular septal thickening	3	12.44 ^ORs^	3.92‒39.48	<0.05	4.7 %	0.350
Lattice shadows	3	35.34 ^ORs^	10.51‒118.82	<0.05	44.3 %	0.166
MPO-ANCA	5	1.39 ^ORs^	1.14‒1.69	<0.05	16.0 %	0.313
Ear, nose & throat	3	0.34 ^ORs^	0.21‒0.55	<0.05	0.0 %	0.425
Fever	2	0.57 ^ORs^	0.34‒0.96	<0.05	0.0 %	0.799
Cough	3	1.60 ^ORs^	1.15‒2.23	<0.05	48.8 %	0.142
Dyspnea	2	5.96 ^ORs^	1.94‒18.3	<0.05	0.0 %	0.653
Peripheral neuropathy	3	1.55 ^ORs^	0.41‒5.84	0.518	69.3 %	<0.1
Rash	2	0.92 ^ORs^	0.35‒2.39	0.861	64.7 %	<0.1
Haematuria	2	1.93 ^ORs^	0.99‒3.76	0.052	0.0 %	0.390

### Sociodemographic factors

Among sociodemographic factors, older age and being male were found to be the most significantly related to AAV-ILD. With respect to older age in 10 studies, a correlation between older age and greater frequency for AAV-ILD was found (WMDs = 8.76, 95 % CI: 5.95–11.57; *p* < 0.05, Fig. 2). ^16^^,^^19^^,^^25^, ^26^, ^27^^,^^29^^,^^33^^,^^38^, ^39^, ^40^ Meanwhile, patients with AAV-ILD were inclined to be male in 3 studies (ORs = 1.65, 95 % CI: 1.20–2.28; *p* < 0.05, Fig. 3). ^16^^,^^17^^,^^35^ However, Flores-Suárez et al.^43^ found that female sex was linked to AAV-ILD.

### Lifestyle factors

Results from the present study indicated that having a smoking history had a significant association with AAV-ILD (ORs = 2.21, 95 % CI: 1.61–3.04; *p* < 0.05, Fig. 4). ^11^^,^^16^^,^^17^^,^^36^ In addition to smoking, Yang et al.^16^ found that BMI was not linked to AAV-ILD.

### Clinical factors

Having honeycombing, lattice shadows, interlobular septal thickening, positive MPO-ANCA, lower ESR, higher KL-6, higher Hb, moderate and high BVAS, Ear, nose & throat, fever, dyspnea, cough, and alb have a significant association with AAV-ILD. The pooled estimate indicated that moderate and high honeycombing, lattice shadows and interlobular septal thickening could be a notable risk factor for AAV-ILD (ORs = 59.53, 95 % CI: 8.42‒420.92; *p* < 0.05, Fig. 5), ^12^^,^^27^^,^^35^ (ORs = 35.34, 95 % CI: 10.51‒118.82; *p* < 0.05, Fig. 6), ^12^^,^^27^^,^^35^ (ORs = 12.44, 95 % CI: 3.92‒39.48; *p* < 0.05, Fig. 7), ^12^^,^^27^^,^^35^ respectively. In studies reporting ORs, positive MPO-ANCA displayed a significant correlation with AAV-ILD (ORs = 1.39, 95 % CI: 1.14‒1.69; *p* < 0.05, Fig. 8). ^25^^,^^26^^,^^29^^,^^38^^,^^41^ Furthermore, three studies showed that higher ESR was correlated with AAV-ILD (WMDs = 21.83, 95 % CI: 7.67‒36.00; *p* < 0.05, Fig. 9). ^25^^,^^26^^,^^43^ Several studies reporting WMDs showed higher KL-6 and AAV-ILD were significantly associated (WMDs = 400.05, 95 % CI: 285.20‒514.91; *p* < 0.05, Fig. 10). ^18^^,^^30^^,^^32^^,^^34^^,^^35^^,^^37^^,^^39^^,^^40^ Two studies reporting WMDs revealed a positive Hb status was more probable to be AAV-ILD (WMDs = 0.91, 95 % CI: 0.37‒1.45; *p* < 0.05, Fig. 11). ^34^^,^^43^ BVAS was associated with AAV-ILD (WMDs = −2.83, 95 % CI: −4.20‒1.45; *p* < 0.05, Fig. 12). ^16^^,^^34^^,^^38^^,^^40^^,^^41^ Additionally, the pooled results manifested that Ear, nose & throat was an important risk factor (ORs = 0.34, 95 % CI: 0.21‒0.55; *p* < 0.05, Fig. 13). ^11^^,^^29^^,^^41^ Fever, dyspnea and cough were associated with AAV-ILD (ORs = 0.57, 95 % CI: 0.34‒0.96; *p* < 0.05, Fig. 14), ^11^^,^^12^ (ORs = 5.96, 95 % CI: 1.94‒18.3; *p* < 0.05, Fig. 15), ^25^^,^^31^ (ORs = 1.60, 95 % CI: 1.15‒2.23; *p* < 0.05, Fig. 16), ^25^^,^^26^^,^^29^ respectively. Finally, two studies revealed a notable correlation between alb and AAV-ILD (WMDs = 0.22, 95 % CI: 0.04‒0.41; *p* < 0.05, Fig. 17). ^34^^,^^39^

In contrast, pulmonary comorbidities, positive PR3-ANCA, use of plasma exchange, myalgias, DAH, CRP, Cr, rash, peripheral neuropathy, and haematuria showed no correlation with AAV-ILD (*p* > 0.05, Fig. 18‒27). Meta-analysis was not performed because there was only one report on these risk factors, including bloody sputum, shortness of breath, nasal crusting, acute respiratory failure, disease duration, GM-CSF, IL-2, CCL-2, CEA, CA199, pulmonary diffusion dysfunction, central neuropathy, eye involvement, head and neck involvement, chest pain, thyroid gland involvement, the percentage of FFS ≥ 3, follow-up, cutaneous, cardiac, cardiovascular, gastrointestinal, pulmonary nodules, mononeuropathy multiplex, immunosuppressive agent, cyclophosphamide, rituximab, methotrexate (Methotrexate), mycophenolate mofetil (CellCept), general symptom involvement, renal involvement, GGO, rs35705950T, respiratory symptoms, FVC (%), DLCO (%), TLC, traction bronchiectasis, pleural effusion, emphysema, bronchiectasis, micronodules, centrilobular nodules and HRCT score.

### Sensitivity analysis

The combined WMD values for each risk factor (age, BVAS) with significant heterogeneity remained consistent with the above results, even after testing using a skew one sensitivity analysis, suggesting that the results were stable (Supplement E). However, for KL-6 and ESR, the sensitivity analysis results suggested the presence of heterogeneity (Supplement E). For KL-6, 3 out of 8 studies indicated sources of heterogeneity; for ESR, 1 out of 3 studies suggested a source of heterogeneity.

### Subgroup analysis and meta-regression

In terms of study design type, subgroup analysis revealed that the risk factors of AAV-ILD in the case-control study studies within age, KL-6, ESR, and BVAS were (WMDs = 11.18, 95 % CI: 6.59‒15.78), (WMDs = 540.94, 95 % CI: 355.27‒726.60), (WMDs = 27.79, 95 % CI: 15.12‒40.45), and (WMDs = −5.40, 95 % CI: −9.56‒−1.24), respectively. The risk factors of AAV-ILD within age, KL-6, and BVAS in cohort studies were (WMDs = 7.05, 95 % CI: 3.13‒10.97), (WMDs = 257.34, 95 % CI: 157.20‒357.49), and (WMDs = −2.61, 95 % CI: −4.04‒−1.18), respectively (Table 2, Table 3, Table 4). The risk factors of AAV-ILD within age, KL-6, and ESR in cross-sectional studies were (WMDs = 4.28, 95 % CI: 1.06‒7.50), (WMDs = 256.08, 95 % CI: 158.14‒354.02), and (WMDs = 12.00, 95 % CI: 2.32‒21.68), respectively (Table 3, Table 4, Table 5, Table 6), (Supplement F).

**Table 3:** Subgroup analysis of the age and subgroup differences.

Subgroup	Number of trials (N)	Effect size	95 %CI	*p*	Test (s) of heterogeneity		Univariate meta regression (*p*)
					*I*^2^	p-value	
Country							
China	6	11.41 ^WMDs^	7.22‒15.60	<0.05	81.8 %	<0.1	Ref
America	1	9.00 ^WMDs^	6.14‒11.86	<0.05	NA	<0.1	0.635
Japan	3	3.81 ^WMDs^	1.40‒6.22	<0.05	0.0 %	0.913	0.055
Study design type							
Case-control study	6	11.18 ^WMDs^	6.59‒15.78	<0.05	83.8 %	<0.1	Ref
Cohort study	2	7.05 ^WMDs^	3.13‒10.97	<0.05	71.0 %	0.063	0.339
Cross-sectional study	3	4.28 ^WMDs^	1.06‒7.50	<0.05	0.0 %	1.000	0.146
Sample							
≥100	5	9.91 ^WMDs^	6.68‒13.15	<0.05	75.6 %	<0.1	0.447
<100	5	7.46 ^WMDs^	2.32‒12.59	<0.05	82.0 %	<0.1	Ref
Follow-up (time)							
≥1 year	3	7.01 ^WMDs^	4.57‒9.46	<0.05	29.6 %	0.242	0.504
Not report	7	9.71 ^WMDs^	5.50‒13.92	<0.05	84.5 %	<0.1	Ref
Types of AAV							
AAV	7	10.88 ^WMDs^	7.59‒14.17	<0.05	78.2 %	<0.1	0.050
MPA	3	3.81 ^WMDs^	5.95‒11.58	<0.05	0.0 %	0.91	Ref

**Table 4:** Subgroup analysis of the KL-6 and subgroup differences.

Subgroup	Number of trials (N)	Effect size	95 % CL	*p*	Test (s) of heterogeneity	
					*I*^2^	*p*-value
Country						
China	2	466.08^WMDs^	401.90‒530.27	<0.05	0.0 %	0.542
Italy	1	667.40^WMDs^	271.49‒1063.31	<0.05	0.0 %	<0.1
Japan	5	340.34^WMDs^	204.43‒ 476.24	<0.05	86.9 %	<0.1
Study design type						
Case-control study	5	540.94 ^WMDs^	355.27‒726.60	<0.05	87.6 %	<0.1
Cohort study	2	257.34 ^WMDs^	157.20‒357.49	<0.05	68.1 %	0.077
Cross-sectional study	1	256.08^WMDs^	158.14‒354.02	<0.05	0.0 %	<0.1
Sample						
≥100	2	257.34^WMDs^	157.20‒357.49	<0.05	68.1 %	0.077
< 100	6	478.47^WMDs^	320.29‒636.64	<0.05	88.2 %	<0.1
Types of AAV						
AAV	4	630.618^WMDs^	427.41‒833.83	<0.05	83.6	<0.1
MPA	4	257.953^WMDs^	211.31‒304.61	<0.05	7.2 %	0.357
Quality score						
High	3	265.238^WMDs^	400.272‒58.159	<0.05	45.0 %	0.122
Low	5	629.215^WMDs^	200.283‒330.193	<0.05	88.6 %	<0.1

**Table 5:** Subgroup analysis of the BVAS and subgroup differences.

Subgroup	Number of trials (N)	Effect size	95 % CL	*p*	Test (s) of heterogeneity	
					*I*^2^	*p*-value
Country						
China	1	−3.30^WMDs^	−4.93‒−1.68	<0.05	0.0 %	<0.1
America	1	−1.00 ^WMDs^	−1.441‒−0.56	<0.05	0.0 %	<0.1
Japan	3	−3.03^WMDs^	−3.32‒−2.73	<0.05	0.0 %	0.428
Study design type						
Case-control study	1	−5.40^WMDs^	−9.56‒−1.24	<0.05	0.0 %	<0.1
Cohort study	4	−2.61^WMDs^	−4.04‒−1.18	<0.05	94.7 %	<0.1
Sample						
≥100	4	−2.61^WMDs^	−4.04‒−1.18	<0.05	94.7 %	<0.1
<100	1	−5.40^WMDs^	−9.56‒−1.24	<0.05	0.0 %	<0.1
Follow-up (time)						
≥1 year	1	−3.30^WMDs^	−4.93‒−1.68	<0.05	0.0 %	<0.1
Not report	4	−2.73^WMDs^	−4.31‒−1.14	<0.05	94.8 %	<0.1
Types of AAV						
AAV	3	−2.357 ^WMDs^	−3.97‒−0.743	<0.05	96.4 %	<0.1
MPA	2	−4.032 ^WMDs^	−5.87‒−2.20	<0.05	0.0 %	0.472

**Table 6:** Subgroup analysis of the ESR and subgroup differences.

Subgroup	Number of trials (N)	Effect size	95 % CL	*p*	Test (s) of heterogeneity	
					*I*^2^	*p*-value
Country						
China	2	27.79^WMDs^	15.12‒40.45	<0.05	57.9 %	0.123
Mexico	1	12.00^WMDs^	2.32‒21.69	<0.05	0.0 %	<0.1
Study design type						
Case-control study	2	27.79^WMDs^	15.12‒40.45	<0.05	57.9 %	0.123
Cross-sectional study	1	12.00^WMDs^	2.32‒21.68	<0.05	0.0 %	<0.1
Sample						
≥ 100	1	32.90^WMDs^	24.84‒40.96	<0.05	0.0 %	<0.1
< 100	2	14.27^WMDs^	6.16‒22.39	<0.05	0.0 %	0.400

Subgroup analysis regarding the sample, the risk factor with sample ≥ 100 were (WMDs = 9.91, 95 % CI: 6.68‒13.15) within age, (WMDs = 257.34, 95 % CI: 157.20‒357.49) within KL-6, (WMDs = 32.90, 95 % CI: 24.84‒40.96) within ESR, and (WMDs = −2.61, 95 % CI: −4.04‒−1.18) within BVAS. In those sample < 100 were (WMDs = 7.46, 95 % CI: 2.32‒12.59), (WMDs = 478.47, 95 % CI: 320.29‒636.64), (WMDs = 14.27, 95 % CI: 6.16‒22.39), and (WMDs = −5.40, 95 % CI: −9.56‒−1.24), respectively (Table 3, Table 4, Table 5, Table 6), (Supplement F).

The Country within age, KL-6, ESR, and BVAS were (WMDs = 11.41, 95 % CI: 7.22‒15.60), (WMDs = 466.08, 95 % CI: 401.90‒530.27), (WMDs = 27.79, 95 % CI: 15.12‒40.45), and (WMDs = −3.30, 95 % CI: −4.93‒−1.68) in the China, respectively. In Japan, the age, KL-6, and BVAS were (WMDs = 3.81, 95 % CI: 1.40–6.22), (WMDs = 340.34, 95 % CI: 204.43‒476.24), and (WMDs = −3.03, 95 % CI: −3.32‒−2.73), respectively. The age and BVAS were (WMDs = 9.00, 95 % CI: 6.14‒11.86) and (WMDs = −1.00, 95 % CI: −1.441‒−0.56) in America. The KL-6 was (WMDs = 667.40, 95 % CI: 271.49‒1063.31) in Italy. The ESR was (WMDs = 12.00, 95 % CI: 2.32‒21.69) in Mexico (Table 3, Table 4, Table 5, Table 6 and Supplement F).

The subgroup analysis of follow-up (time) ≥ 1-year within age was (WMDs = 7.01, 95 % CI: 4.57‒9.46) and (WMDs = −3.30, 95 % CI: −4.93‒−1.68) within BVAS, respectively. The subgroup results without follow-up (time) were (WMDs = 9.71, 95 % CI: 5.50‒13.92) and (WMDs = −2.73, 95 % CI: −4.31‒−1.14), respectively (Table 3, Table 4, Table 5, Table 6 and Supplement F).

The type of AAV within age, KL-6, and BVAS were (WMDs = 10.88, 95 % CI: 7.59‒14.17), (WMDs = 630.618, 95 % CI: 427.41‒833.83), and (WMDs = −2.357, 95 % CI: −3.97‒−0.743) in the AAV, respectively. The subgroup results without MPA were (WMDs = 3.81, 95 % CI: 5.95‒11.58), (WMDs = 257.953, 95 % CI: 211.31‒304.61), and (WMDs = −4.032, 95 % CI: −5.87‒−2.20), respectively (Table 3, Table 4, Table 5, Table 6 and Supplement F).

Subgroup analysis regarding Quality score, On the one hand, high quality score within KL-6 was (WMDs = 265.238, 95 % CI: 400.272‒58.159), on the other hand, low quality score within KL-6 was (WMDs = 629.215, 95 % CI: 200.283‒330.193) (Tables 4 and Supplement F).

No subgroup differences in follow-up (time), Country, sample, type of AAV and study design type within age were found (Table 3, Table 4, Table 5, Table 6 and Supplement E).

### Publication bias

A total of 10 studies examined the age factor, with meta-analysis revealing statistically significant differences (*p* < 0.05; see Table 2 and Fig. 2). Egger’s tests indicated no significant publication bias in risk factor of AAV-ILD within age (*p* = 0.203, Fig. 28). Meanwhile, there was no publication bias within age (*p* = 0.064, Fig. 29). Although Egger’s test indicated no significant publication bias, trim-and-fill analysis was performed due to the observed funnel plot asymmetry. Based on the original meta-analysis of 10 studies (*p* < 0.05), one imputed study was added, and the analysis was repeated. As shown in Figure 30, the result remained statistically significant (*p* < 0.05) without reversal after adding the imputed study, indicating that the pooled result is robust.

## Discussion

The occurrence of ILDs in AAV patients has posed a serious social and economic burden compared with AAV patients without ILD, significantly impacting public healthcare systems worldwide. 44, 45, 46 This study is the first to systematically summarize and elucidate the potential risk factors for the development of ILDs in AAV, addressing a critical gap in the literature and providing a foundation for future research and clinical practice. The overall quality of the 25 studies was assessed favorably. The systematic evaluation and meta-analysis showed that pooled of the occurrence of ILDs in AAV.

Of all 71 factors, 27 were included in our meta-analysis, while the remaining factors were analyzed qualitatively. The authors identified 17 potential risk factors associated with AAV-ILD: age, male, having a smoking history, honeycombing, lattice shadows, interlobular septal thickening, positive MPO-ANCA, lower ESR, higher KL-6, dyspnea, and cough. Moreover, Ear, nose & throat, moderate and high BVAS, higher Hb, fever, and alb were found to be protective factors. Most data contained were multivariable-adjusted effect estimates (OR, WMD, and 95 % CI). Understanding potential risk factors, which could be easily evaluated in day-to-day clinical practice, will help identify at-risk patients, thus paving the way for developing strategies to improve survival and alter the natural course of the disease before clinical symptoms become severe.

With respect to sociodemographic factors and lifestyle factors, the present study found that older age, ^16^^,^^19^^,^^25^, ^26^, ^27^^,^^29^^,^^33^^,^^38^, ^39^, ^40^ males, ^16^^,^^17^^,^^35^ and having a smoking history^11^^,^^16^^,^^17^^,^^36^ tended to be AAV-ILD, which is also consistent with previous studies. ^15^ These observations are consistent with previous reports from around the world, including recent reports from Latin America, where it has also been found that a significant proportion of patients may present with an ILD prior to the development of other disease features. ^47^ Although women are at a risk of developing ILD in the present study, ^43^ males are inclined to predominate among patients with AAV-ILD. Similar to Idiopathic Pulmonary Fibrosis (IPF), our findings also revealed the role of smoking in the development of progressive fibrotic ILD in AAV patients, as AAV-ILD cases smoked more frequently than the AAV-non-ILD group. ^48^^,^^49^ This may be related to the likelihood of classification of AAV-ILD due to the appearance of clinical symptoms or more frequent CT scanning in AAV individuals. These findings have important clinical ramifications. Since smoking is an emerging modifiable potential risk factor for AAV-ILD, clinicians can recommend their patients on lifestyle habit changes that can lead to a more favorable health outcome.

In addition, clinical factors were prone to be AAV-ILD, including having positive MPO-ANCA, lower ESR, higher KL-6, higher Hb, moderate and high BVAS, fever, Ear, nose & throat, dyspnea, cough, alb, and HRCT findings such as honeycombing, interlobular septal thickening, and lattice shadows. Regarding clinical manifestations, our studies observed that the risk of AAV-ILD was elevated by clinical manifestations, such as Ear, nose & throat, fever, dyspnea, and cough at baseline. ^11^^,^^29^^,^^41^ This might be due to the arising of pulmonary inflammatory response by the underlying clinical manifestations, resulting in persistent lung injury, thereby increasing the risk of ILD. In recent years, other pulmonary clinical manifestations like alveolar hemorrhage, have been revealed as potential contributors to the occurrence of AAV-ILD. ^36^^,^^43^ Chung et al.^50^ observed that pulmonary involvement may occur in 25 % to 55 % of AAV patients, with DAH being the most common manifestation. Therefore, promoting the awareness of pulmonary clinical manifestations may have a beneficial influence on the public’s health.

Apart from clinical manifestations, laboratory parameters displayed a significant correlation with AAV-ILD, such as positive MPO-ANCA, higher KL-6, lower ESR, and higher Hb. Positive MPO-ANCA was related to AAV-ILD. ^51^ To our knowledge, when diagnosing AAV, the seropositivity of MPO-ANCA is favorable. In Asian countries, ILD in AAV is more commonly associated with MPO-ANCA than in Western countries, possibly owing to the lower latitude of Asian countries. 52, 53, 54 Therefore, given the frequency of ILD in AAV, especially positive MPO-ANCA in AAV, it makes clinical sense for rheumatologists, pulmonologists, and others who routinely manage AAV to consider laboratory screening for ILD, even in those patients who are asymptomatic. Methods of laboratory screening, including the way and frequency, need further study. Concerns about a correlation with KL-6 and the deterioration of AAV-ILD have been emphasized in several recent studies. ^18^^,^^35^^,^^37^ Several studies have observed higher KL-6 in AAV patients with ILD than in patients without lung involvement, suggesting that KL-6 may be a clinically meaningful prognostic marker. ^55^^,^^56^ Members of the MUC family, particularly MUC1 (such as KL-6), are considered to be key effector during tissue remodeling and cell growth, consistent with the process of pulmonary fibrosis. ^57^ A threshold value of 465 U/mL for KL-6 was established to distinguish patients with fibrotic ILD from healthy individuals and other patients with non-fibrotic lung disease. ^58^ Therefore, serum KL-6 level can be used as a sensitive and specific biological indicator for the diagnosis of AAV combined with ILD, and can be used to monitor the changes of AAV combined with ILD; meanwhile, the persistent elevation of serum KL-6 level may indicate that the prognosis of this disease is poor. In this study, the authors found that ESR was associated with ILD in AAV patients. Elevated ESR suggests that AAV patients have active disease, which is a guide for prognosis and observation of the efficacy of treatment. Apart from ANCA positivity, patients with AAV usually have a higher ESR, and patients with pulmonary fibrosis usually do not have isolated ANCA positivity. ^59^ One study^8^ revealed that elevated ESR was independently associated with poor prognosis and that patients in the AAV-ILD group with elevated ESR had a poorer prognosis than those with normal inflammatory markers. The results of the present study also confirmed this finding, with elevated ESR being more pronounced in the AAV-ILD group compared to the AAV group. Two studies have reported that Hb^34^^,^^43^ and alb^27^^,^^39^ are more likely to be AAV-ILD compared with AAV patients, respectively.

BVAS as an indicator of disease activity has application for AAV-ILD diagnosis and monitoring. Total BVAS is related to a bad prognosis in AAV. ^60^ Compared with AAV patients without ILD, BVAS was apparently lower in the AAV patients with ILD. ^16^^,^^38^^,^^41^ Therefore, it is necessary to perform BVAS in patients with AAV and to evaluate the organ involvement. Moreover, ILD is one of the most common manifestations of pulmonary involvement in AAV, and HRCT has become one of the main methods of clinical evaluation of ILD. ^61^ It has been suggested that the presence of a baseline pulmonary honeycomb shadow is associated with a high risk of late infection in patients with AAV, thereby increasing the risk of patient mortality. ^62^ In the present study, patients with AAV had a variety of minor HRCT findings, such as honeycombing, interlobular septal thickening, and lattice shadows. These HRCT findings can assist radiologists and pulmonologists to consider radiologic diagnosis of AAV with UIP. ^12^^,^^27^^,^^35^ Patients with AAV should be alerted to the possibility of combined ILD if they present early with symptoms of pulmonary involvement (e.g., HRCT manifestations such as honeycombing, interlobular septal thickening, and lattice shadows).

In contrast, the results of meta-analysis showed that pulmonary comorbidities, PR3-ANCA positivity, use of plasma exchange, myalgia, DAH, CRP, Cr, rash, peripheral neuropathy, and hematuria did not correlate with AAV-ILD (*p* > 0.05). This may be related to the fact that too few studies were included. The large differences between studies may be related to the number of cases and the source of the cases, to be confirmed by further follow-up studies. Meanwhile, meta-analysis was not performed because there was only one report on these risk factors, including bloody sputum, shortness of breath, nasal crusting, acute respiratory failure, disease duration, GM-CSF, IL-2, CCL-2, CEA, CA199, pulmonary diffusion dysfunction, central neuropathy, eye involvement, head and neck involvement, chest pain, thyroid gland involvement, the percentage of FFS ≥3, follow-up, cutaneous, cardiac, cardiovascular, gastrointestinal, pulmonary nodules, mononeuropathy multiplex, immunosuppressive agent, cyclophosphamide, rituximab, methotrexate (Methotrexate), mycophenolate mofetil (CellCept), general symptom involvement, renal involvement, GGO, rs35705950T, respiratory symptoms, FVC (%), DLCO (%), TLC, traction bronchiectasis, pleural effusion, emphysema, bronchiectasis, micronodules, centrilobular nodules and HRCT score. Further research on these factors may be needed in the future.

Finally, the sensitivity analysis results indicate that the factors of age and BVAS did not show significant bias, while the factors of KL-6 and ESR suggested the presence of bias. The observed heterogeneity in the sensitivity analysis may stem from several sources. Variations in study design, such as differences in sample selection criteria or measurement protocols for KL-6 and ESR, could contribute to the inconsistency. Additionally, demographic and clinical characteristics of the study populations, such as age, disease severity, or comorbidities, might have influenced the results. To address the heterogeneity, the authors performed subgroup analyses based on study design and population characteristics. The results showed that the heterogeneity was significantly reduced in studies with standardized measurement protocols, suggesting that methodological differences might be a major source of inconsistency. Furthermore, sensitivity analysis excluding one outlier study for ESR demonstrated improved consistency, supporting the robustness of the primary findings. Despite our efforts to address heterogeneity through subgroup and sensitivity analyses, some limitations remain. The variability in measurement protocols for KL-6 and ESR across studies may have contributed to the observed inconsistency. Future research should aim to standardize measurements of key variables and include more homogeneous populations to reduce heterogeneity, enhancing the reliability of the findings. These efforts will enhance the reliability and generalizability of the findings, providing more robust evidence for clinical decision-making and public health interventions.

## Limitations

There are several limitations that need to be acknowledged. Firstly, this study's limitation to English and Chinese publications may introduce language bias. While this choice was made to ensure linguistic accuracy in data extraction (particularly for culture-dependent variables) and feasibility of translation quality control, the authors acknowledge that language restrictions may have excluded 2 %‒15 % of potentially eligible studies. Future meta-analyses should incorporate major WHO official languages (Arabic, French, Russian, and Spanish), ideally through multilingual research consortia to mitigate translation bias. Second, Significant heterogeneity was observed among the included studies, particularly for the biomarkers KL-6, ESR, and BVAS. This variability may be associated with differences in standardization of KL-6 and ESR assay methodologies, as well as discrepancies in BVAS scoring versions. While the subgroup analyses based on follow-up duration, country, sample size, and study design did not reveal significant subgroup differences, this supports the reliability of these results. Meanwhile, a concordant trend persisted in the pre-specified high-methodological-quality subgroup (NOS ≥7), reinforcing the robustness of our findings. Third, the risk factor estimates were based on case-control studies, cohort studies, and cross-sectional studies, which may affect the robustness of our meta-analysis results. Two key limitations of cross-sectional designs for causal inference must be acknowledged, including the inability to establish temporality and the lack of dynamic assessment of interactions. ^63^ Therefore, these findings can only suggest associations between KL-6, ESR, BVAS, and the severity of pulmonary fibrosis, while the causal directions require further validation through subsequent cohort studies or Mendelian randomization analyses. Fourth, while this review provides a comprehensive synthesis of existing evidence, the methodological quality of the included studies varied. Notably, only a proportion of the included studies explicitly reported obtaining informed consent and institutional ethical approval. This lack of reporting does not necessarily imply ethical misconduct, but it highlights a significant gap in transparent research practice and may raise concerns about the potential for selection bias in those individual studies. Finally, it is important to note that some risk factors were investigated in only a small number of studies, which may limit the statistical power and reliability of our findings for these factors. The present study rigorously applied GRADE methodology, explicitly labeling evidence of very low certainty and modifying conclusions to be hypothesis-generating only. To strengthen evidence reliability, the authors recommend that future studies: (a) Emphasize enrollment in adequately powered prospective cohorts (target *n* ≥ 500 per arm), (b) Utilize IPD meta-analytic approaches to address ecological bias, and (c) Integrate small-study effects evaluation into GRADE frameworks.

## Conclusions

This meta-analysis provides a comprehensive summary of the knowledge regarding AAV-ILD patients and the associated risk factors. Our findings indicate that the onset of AAV-ILD is associated with a range of lifestyle, sociodemographic, and clinical factors, including age, male, smoking history, KL-6, ESR, MPO-ANCA, cough, dyspnea, and HRCT findings such as honeycombing, interlobular septal thickening, and lattice shadows, Ear, nose & throat involvement, Hb, alb, BVAS, and fever. It is urgent to comprehensively identify the risk factors for AAV-ILD and to implement timely strategies for prevention and management to address both persistent and emerging cases of AAV-ILD. These findings may have outstanding public health significance as they offer meaningful insights for conducting valuable interventions, which can guide clinical prevention and management with the target of diminishing the risk of AAV-ILD. However, limitations including small sample sizes, substantial heterogeneity, the predominance of low-quality cross-sectional studies, and language restrictions (only English and Chinese publications included) may affect the robustness of our findings. While these results may serve as a reference for future research, prospective large-scale cohort studies or longitudinal designs are warranted for validation.

## Funding

This study has been supported by the Young Scientists Fund of the National Science Foundation (No. 82,505,809), the National Science Foundation of China (No. 82,174,307), and the Young Scientists Fund of the National Science Foundation (No. 82,305,138). All the participant experts are appreciated for their diligence in this study.

## Data availability

All data produced or analyzed in this study are included in this paper and in the supplemental data.

## CRediT authorship contribution statement

**Ningxia Yu:** Writing – original draft, Writing – review & editing. **Shuguang Yang:** Data curation, Methodology. **Danyang Zang:** Visualization, Validation. **Lili Xu:** Data curation, Methodology. **Ruonan Yan:** Visualization, Validation. **Xueqing Yu:** Conceptualization.

## Declaration of competing interest

The authors declare no conflicts of interest.

## Appendix Supplementary materials

Image, application 1
