# Supplementary material for: The risk factors of anti-neutrophil cytoplasmic antibody-associated vasculitis-associated interstitial lung disease: a systematic review and meta-analysis
Source: Clinics (Sao Paulo). 2025 Nov 5;80:100823. doi: 10.1016/j.clinsp.2025.100823 (PMC12637398; doi:10.1016/j.clinsp.2025.100823)

CLINICS-D-25-00336_Supplementary Material

**Appendix A** The risk factors of anti-neutrophil cytoplasmic antibody-associated vasculitis-associated interstitial lung disease: a systematic review and meta-analysis.

**Supplement A** PRISMA checklist.

| **Section and Topic** | **Item #** | **Checklist item** | **Location where item is reported** |
| --- | --- | --- | --- |
| **TITLE** | | |  |
| Title | 1 | Identify the report as a systematic review. | p1 |
| **ABSTRACT** | | |  |
| Abstract | 2 | See the PRISMA 2020 for Abstracts checklist. | P2 |
| **INTRODUCTION** | | |  |
| Rationale | 3 | Describe the rationale for the review in the context of existing knowledge. | P2 |
| Objectives | 4 | Provide an explicit statement of the objective(s) or question(s) the review addresses. | p3 |
| **METHODS** | | |  |
| Eligibility criteria | 5 | Specify the inclusion and exclusion criteria for the review and how studies were grouped for the syntheses. | p4 |
| Information sources | 6 | Specify all databases, registers, websites, organisations, reference lists and other sources searched or consulted to identify studies. Specify the date when each source was last searched or consulted. | p4 |
| Search strategy | 7 | Present the full search strategies for all databases, registers, and websites, including any filters and limits used. | p4 |
| Selection process | 8 | Specify the methods used to decide whether a study met the inclusion criteria of the review, including how many reviewers screened each record and each report retrieved, whether they worked independently, and if applicable, details of automation tools used in the process. | p5 |
| Data collection process | 9 | Specify the methods used to collect data from reports, including how many reviewers collected data from each report, whether they worked independently, any processes for obtaining or confirming data from study investigators, and if applicable, details of automation tools used in the process. | p5 |
| Data items | 10a | List and define all outcomes for which data were sought. Specify whether all results that were compatible with each outcome domain in each study were sought (e.g., for all measures, time points, analyses), and if not, the methods used to decide which results to collect. | p5 |
|  | 10b | List and define all other variables for which data were sought (e.g., participant and intervention characteristics, funding sources). Describe any assumptions made about any missing or unclear information. | p5 |
| Study risk of bias assessment | 11 | Specify the methods used to assess risk of bias in the included studies, including details of the tool(s) used, how many reviewers assessed each study and whether they worked independently, and if applicable, details of automation tools used in the process. | p5 |
| Effect measures | 12 | Specify for each outcome the effect measure(s) (e.g., risk ratio, mean difference) used in the synthesis or presentation of results. | p5 |
| Synthesis methods | 13a | Describe the processes used to decide which studies were eligible for each synthesis (e.g., tabulating the study intervention characteristics and comparing against the planned groups for each synthesis (item #5)). | p5 |
|  | 13b | Describe any methods required to prepare the data for presentation or synthesis, such as handling of missing summary statistics, or data conversions. | p5 |
|  | 13c | Describe any methods used to tabulate or visually display results of individual studies and syntheses. | p5 |
|  | 13d | Describe any methods used to synthesize results and provide a rationale for the choice(s). If meta-analysis was performed, describe the model(s), method(s) to identify the presence and extent of statistical heterogeneity, and software package(s) used. | p5 |
|  | 13e | Describe any methods used to explore possible causes of heterogeneity among study results (e.g., subgroup analysis, meta-regression). | p5 |
|  | 13f | Describe any sensitivity analyses conducted to assess robustness of the synthesized results. | p5 |
| Reporting bias assessment | 14 | Describe any methods used to assess risk of bias due to missing results in a synthesis (arising from reporting biases). | p5 |
| Certainty assessment | 15 | Describe any methods used to assess certainty (or confidence) in the body of evidence for an outcome. | p5 |
| **RESULTS** | | |  |
| Study selection | 16a | Describe the results of the search and selection process, from the number of records identified in the search to the number of studies included in the review, ideally using a flow diagram. | P6-7 |
|  | 16b | Cite studies that might appear to meet the inclusion criteria, but which were excluded, and explain why they were excluded. | P6-7 |
| Study characteristics | 17 | Cite each included study and present its characteristics. | P6-7 |
| Risk of bias in studies | 18 | Present assessments of risk of bias for each included study. | P6-7 |
| Results of individual studies | 19 | For all outcomes, present, for each study: (a) summary statistics for each group (where appropriate) and (b) an effect estimate and its precision (e.g. confidence/credible interval), ideally using structured tables or plots. | P6-7 |
| Results of syntheses | 20a | For each synthesis, briefly summarize the characteristics and risk of bias among contributing studies. | P8-19 |
|  | 20b | Present results of all statistical syntheses conducted. If meta-analysis was done, present for each the summary estimate and its precision (e.g., confidence/credible interval) and measures of statistical heterogeneity. If comparing groups, describe the direction of the effect. | P8-19 |
|  | 20c | Present results of all investigations of possible causes of heterogeneity among study results. | P8-19 |
|  | 20d | Present results of all sensitivity analyses conducted to assess the robustness of the synthesized results. | P8-19 |
| Reporting biases | 21 | Present assessments of risk of bias due to missing results (arising from reporting biases) for each synthesis assessed. | P20 |
| Certainty of evidence | 22 | Present assessments of certainty (or confidence) in the body of evidence for each outcome assessed. | P20 |
| **DISCUSSION** | | |  |
| Discussion | 23a | Provide a general interpretation of the results in the context of other evidence. | P21-26 |
|  | 23b | Discuss any limitations of the evidence included in the review. | p21-26 |
|  | 23c | Discuss any limitations of the review processes used. | p21-26 |
|  | 23d | Discuss implications of the results for practice, policy, and future research. | p21-26 |
| **OTHER INFORMATION** | | |  |
| Registration and protocol | 24a | Provide registration information for the review, including the register name and registration number, or state that the review was not registered. | P2 |
|  | 24b | Indicate where the review protocol can be accessed, or state that a protocol was not prepared. | - |
|  | 24c | Describe and explain any amendments to information provided at registration or in the protocol. | - |
| Support | 25 | Describe sources of financial or non-financial support for the review, and the role of the funders or sponsors in the review. | P27 |
| Competing interests | 26 | Declare any competing interests of review authors. | P27 |
| Availability of data, code and other materials | 27 | Report which of the following are publicly available and where they can be found: template data collection forms; data extracted from included studies; data used for all analyses; analytic code; any other materials used in the review. | p6 |

From: Page MJ, McKenzie JE, Bossuyt PM, Boutron I, Hoffmann TC, Mulrow CD, et al. The PRISMA 2020 statement: an updated guideline for reporting systematic reviews. BMJ 2021;372:n71. doi: 10.1136/bmj.n71. This work is licensed under CC BY 4.0. To view a copy of this license, visit https://creativecommons.org/licenses/by/4.0/.

**Supplement B** Details of the literature search strategy Supplement.

| **Cochrane Library** | | |
| --- | --- | --- |
| #1 | (interstitial lung disease): ti, ab, kw OR (lung fibrosis): ti,ab, kw OR (ILD): ti,ab, kw OR (UIP): ti,ab, kw OR (NSIP): ti,ab,kw (Word variations have been searched) | **6407** |
| #2 | (PPFE): ti,ab,kw OR (pleuroparenchymal fibroelastosis): ti,ab,kw OR (Bronchiolitis): ti,ab,kw OR (Alveolitis): ti,ab,kw OR (pulmonary fibrosis): ti,ab,kw (Word variations have been searched) | **6649** |
| #3 | (anti-neutrophil cytoplasmic antibody-associated vasculitis): ti,ab,kw OR (antineutrophil cytoplasmic antibod*): ti,ab,kw OR (ANCA): ti,ab,kw OR (churg-strauss syndrome): ti,ab,kw OR (eosinophilic granulomatosis with polyangiiti*): ti,ab,kw (Word variations have been searched) | **858** |
| #4 | (granulomatosis with polyangiitis): ti,ab,kw OR (wegener’s granulomatosis): ti,ab,kw OR (Microscopic Polyangiitis): ti,ab,kw OR (wegener*): ti,ab,kw OR (polyangiiti*): ti,ab,kw (Word variations have been searched) | **487** |
| #5 | (polyarteriti*): ti,ab,kw OR (systemic vasculitis): ti,ab,kw OR (pauci immune vasculiti*): ti,ab,kw OR (small vessel vasculiti*): ti,ab,kw (Word variations have been searched) | **758** |
| #6 | (Diffuse Parenchymal Lung Disease): ti,ab,kw OR (Interstitial Pneumonia): ti,ab,kw OR (Interstitial Pneumonitides): ti,ab,kw OR (Interstitial Pneumonitis): ti,ab,kw (Word variations have been searched) | **1105** |
| #7 | #1 OR #2 OR #6 | **10376** |
| #8 | #3 OR #4 | **1002** |
| #9 | #7 AND #8 | **27** |
| **Pubmed** | | |
| #1 | (("anti neutrophil cytoplasmic antibody associated vasculitis"[MeSH Terms] OR ("anti neutrophil"[All Fields] AND "cytoplasmic"[All Fields] AND "antibody associated"[All Fields] AND "vasculitis"[All Fields]) OR "anti neutrophil cytoplasmic antibody associated vasculitis"[All Fields] OR ("anti"[All Fields] AND "neutrophil"[All Fields] AND "cytoplasmic"[All Fields] AND "antibody"[All Fields] AND "associated"[All Fields] AND "vasculitis"[All Fields]) OR "anti neutrophil cytoplasmic antibody associated vasculitis"[All Fields] OR (("antineutrophil"[All Fields] OR "antineutrophilic"[All Fields]) AND ("cytoplasm"[MeSH Terms] OR "cytoplasm"[All Fields] OR "cytoplasmic"[All Fields] OR "cytoplasms"[All Fields] OR "cytoplasmically"[All Fields]) AND "antibod*"[All Fields]) OR ("antibodies, antineutrophil cytoplasmic"[MeSH Terms] OR ("antibodies"[All Fields] AND "antineutrophil"[All Fields] AND "cytoplasmic"[All Fields]) OR "antineutrophil cytoplasmic antibodies"[All Fields] OR "anca"[All Fields]) OR ("churg strauss syndrome"[MeSH Terms] OR ("churg strauss"[All Fields] AND "syndrome"[All Fields]) OR "churg strauss syndrome"[All Fields] OR ("churg"[All Fields] AND "strauss"[All Fields] AND "syndrome"[All Fields]) OR "churg strauss syndrome"[All Fields]) OR (("eosinophil s"[All Fields] OR "eosinophile"[All Fields] OR "eosinophiles"[All Fields] OR "eosinophilic"[All Fields] OR "eosinophillic"[All Fields] OR "eosinophills"[All Fields] OR "eosinophils"[MeSH Terms] OR "eosinophils"[All Fields] OR "eosinophil"[All Fields]) AND "granulomatosis"[All Fields] AND "polyangiiti*"[All Fields]) OR ("granulomatosis with polyangiitis"[MeSH Terms] OR ("granulomatosis"[All Fields] AND "polyangiitis"[All Fields]) OR "granulomatosis with polyangiitis"[All Fields]) OR ("granulomatosis with polyangiitis"[MeSH Terms] OR ("granulomatosis"[All Fields] AND "polyangiitis"[All Fields]) OR "granulomatosis with polyangiitis"[All Fields] OR ("wegener s"[All Fields] AND "granulomatosis"[All Fields]) OR "wegener s granulomatosis"[All Fields]) OR ("microscopic polyangiitis"[MeSH Terms] OR ("microscopic"[All Fields] AND "polyangiitis"[All Fields]) OR "microscopic polyangiitis"[All Fields]) OR "wegener*"[All Fields] OR "polyangiiti*"[All Fields] OR "polyarteriti*"[All Fields] OR ("systemic vasculitis"[MeSH Terms] OR ("systemic"[All Fields] AND "vasculitis"[All Fields]) OR "systemic vasculitis"[All Fields]) OR ("pauci"[All Fields] AND ("immune"[All Fields] OR "immuned"[All Fields] OR "immunes"[All Fields] OR "immunisation"[All Fields] OR "vaccination"[MeSH Terms] OR "vaccination"[All Fields] OR "immunization"[All Fields] OR "immunization"[MeSH Terms] OR "immunisations"[All Fields] OR "immunizations"[All Fields] OR "immunise"[All Fields] OR "immunised"[All Fields] OR "immuniser"[All Fields] OR "immunisers"[All Fields] OR "immunising"[All Fields] OR "immunities"[All Fields] OR "immunity"[MeSH Terms] OR "immunity"[All Fields] OR "immunization s"[All Fields] OR "immunize"[All Fields] OR "immunized"[All Fields] OR "immunizer"[All Fields] OR "immunizers"[All Fields] OR "immunizes"[All Fields] OR "immunizing"[All Fields]) AND "vasculiti*"[All Fields]) OR (("small"[Journal] OR "small"[All Fields]) AND ("blood vessels"[MeSH Terms] OR ("blood"[All Fields] AND "vessels"[All Fields]) OR "blood vessels"[All Fields] OR "vessel"[All Fields] OR "vessels"[All Fields] OR "vessel s"[All Fields] OR "vesselness"[All Fields]) AND "vasculiti*"[All Fields])) | **50779** |
| #2 | ("interstitial lung disease"[Title/Abstract] OR "lung fibrosis"[Title/Abstract] OR "ILD"[Title/Abstract] OR "UIP"[Title/Abstract] OR "NSIP"[Title/Abstract] OR "PPFE"[Title/Abstract] OR "pleuroparenchymal fibroelastosis"[Title/Abstract] OR "Bronchiolitis"[Title/Abstract] OR "Alveolitis"[Title/Abstract] OR "pulmonary fibrosis"[Title/Abstract] OR "Diffuse Parenchymal Lung Disease"[Title/Abstract] OR "Interstitial Pneumonia"[Title/Abstract] OR "Interstitial Pneumonitides"[Title/Abstract] OR "Interstitial Pneumonitis"[Title/Abstract]) | **72052** |
| #3 | #1 and #2 Filters: from database inception to ‒ 2024/9/21 | **820** |
| **Embase** | | |
| #1 | ('anti-neutrophil cytoplasmic antibody-associated vasculitis'/exp OR 'anti-neutrophil cytoplasmic antibody-associated vasculitis' OR ('anti neutrophil' AND cytoplasmic AND 'antibody associated' AND ('vasculitis'/exp OR vasculitis)) OR 'antineutrophil cytoplasmic' OR (antineutrophil AND cytoplasmic AND antibod*) OR 'anca'/exp OR anca OR 'churg-strauss syndrome'/exp OR 'churg-strauss syndrome' OR (('churg strauss'/exp OR 'churg strauss') AND ('syndrome'/exp OR syndrome)) OR 'eosinophilic granulomatosis with' OR (eosinophilic AND ('granulomatosis'/exp OR granulomatosis) AND with AND polyangiiti*) OR 'granulomatosis with polyangiitis'/exp OR 'granulomatosis with polyangiitis' OR (('granulomatosis'/exp OR granulomatosis) AND with AND ('polyangiitis'/exp OR polyangiitis)) OR 'wegener granulomatosis'/exp OR 'wegener granulomatosis' OR (wegener AND ('granulomatosis'/exp OR granulomatosis)) OR 'microscopic polyangiitis'/exp OR 'microscopic polyangiitis' OR (microscopic AND ('polyangiitis'/exp OR polyangiitis)) OR wegener* OR polyangiiti* OR polyarteriti* OR 'systemic vasculitis'/exp OR 'systemic vasculitis' OR (systemic AND ('vasculitis'/exp OR vasculitis)) OR 'pauci immune' OR (pauci AND ('immune'/exp OR immune) AND vasculiti*) OR 'small vessel' OR (small AND ('vessel'/exp OR vessel) AND vasculiti*)) | **123605** |
| #2 | (interstitial AND ('lung'/exp OR lung) AND ('disease'/exp OR disease) OR (('lung'/exp OR lung) AND ('fibrosis'/exp OR fibrosis)) OR ild OR uip OR nsip OR ppfe OR (pleuroparenchymal AND ('fibroelastosis'/exp OR fibroelastosis)) OR bronchiolitis OR 'alveolitis'/exp OR alveolitis OR (pulmonary AND ('fibrosis'/exp OR fibrosis)) OR diffuse AND parenchymal AND 'lung'/exp AND 'disease'/exp OR (interstitial AND 'pneumonia'/exp) OR (interstitial AND pneumonitides) OR (interstitial AND 'pneumonitis'/exp)) | **41276** |
| #3 | #1 and #2 Filters: from database inception to ‒ 2024/9/21 | **1781** |
| **CNKI (From establishment till Sep 21^th^ 2024)** | | |
| 1 | SU=(间质性肺疾病 + 肺间质疾病 + 间质性肺病 + 间质性肺炎 + 肺间质纤维化 + 肺间质病变) | **9975** |
| 2 | SU=血管炎 | **6029** |
| 3 | 1 AND 2 | **96** |
| **CBM (From establishment till Sep 21^th^ 2024)** | | |
| 1 | ("间质性肺疾病"[常用字段:智能] OR "肺间质疾病"[常用字段:智能] OR "间质性肺病"[常用字段:智能] OR "间质性肺炎"[常用字段:智能] OR "肺间质纤维化"[常用字段:智能] OR "肺间质病变"[常用字段:智能]) | **37214** |
| 2 | "血管炎"[常用字段:智能] | **51573** |
| 3 | 1 AND 2 | **430** |
| **VIP** Data (**From establishment till Sep 21^th^ 2024)** | | |
| 1 | M=(间质性肺疾病 or 肺间质疾病 or 间质性肺病 or 间质性肺炎 or 肺间质纤维化 or 肺间质病变) | **8309** |
| 2 | K=(血管炎) | **5516** |
| 3 | 1 AND 2 | **65** |
| WanFang Data (**From establishment till Sep 21^th^ 2024)** | | |
| 1 | 主题:(间质性肺疾病 or 肺间质疾病 or 间质性肺病 or 间质性肺炎 or 肺间质纤维化 or 肺间质病变) | **19298** |
| 2 | 主题:(血管炎) | **15926** |
| 3 | 1 AND 2 | **566** |

**Supplement C** Study quality of cohort study.

| **Author** | **Representativeness of the exposed cohort** | **Selection of the non exposed cohort** | **Ascertainment of exposure** | **Demonstration that outcome of interest was not present at start of study** | **Comparability of cohorts on the basis of the design or analysis** | **Assessment of outcome** | **Was follow-up long enough for outcomes to occur** | **Adequacy of follow up of cohorts** | **Total scores** |
| --- | --- | --- | --- | --- | --- | --- | --- | --- | --- |
| Matsuda[34] | ★ | ★ | ★ | ★ | ☆☆ | ★ | ★ | ★ | 7 |
| Suzuki[35] | ★ | ★ | ★ | ☆ | ★★ | ★ | ☆ | ☆ | 6 |
| Doliner[38] | ★ | ★ | ★ | ☆ | ★★ | ★ | ★ | ★ | 8 |
| Yang[16] | ★ | ★ | ★ | ★ | ★☆ | ★ | ★ | ★ | 8 |
| Sada[41] | ★ | ★ | ★ | ★ | ★☆ | ★ | ☆ | ☆ | 6 |

**Supplement C** Study quality of case-control study.

| **Author** | **Is the case definition adequate?** | **Representativeness of the Cases** | **Selection of Controls** | **Definition of Controls** | **Comparability of Cases and Controls on the Basis of the Design or Analysis** | **Ascertainment of exposure** | **Same method of ascertainment for cases and controls** | **Non-Response rate** | **Total scores** |
| --- | --- | --- | --- | --- | --- | --- | --- | --- | --- |
| Jiang[25] | ★ | ★ | ★ | ★ | ★★ | ☆ | ★ | ★ | 8 |
| Miao[26] | ★ | ★ | ★ | ★ | ★★ | ☆ | ★ | ★ | 8 |
| Wu[27] | ★ | ☆ | ★ | ★ | ★★ | ☆ | ★ | ★ | 7 |
| Zhang[28] | ★ | ☆ | ★ | ★ | ☆☆ | ★ | ★ | ★ | 6 |
| Xie[29] | ★ | ★ | ★ | ★ | ★☆ | ★ | ★ | ★ | 8 |

**Supplement C** Study quality of case-control study. Continued.

| **Author** | **Is the case definition adequate?** | **Representativeness of the Cases** | **Selection of Controls** | **Definition of Controls** | **Comparability of Cases and Controls on the Basis of the Design or Analysis** | **Ascertainment of exposure** | **Same method of ascertainment for cases and controls** | **Non-Response rate** | **Total scores** |
| --- | --- | --- | --- | --- | --- | --- | --- | --- | --- |
| Wang[31] | ★ | ★ | ★ | ★ | ★★ | ☆ | ★ | ☆ | 7 |
| Hozumi[17] | ★ | ★ | ★ | ★ | ★★ | ★ | ★ | ★ | 9 |
| Maillet[11] | ★ | ★ | ★ | ★ | ★☆ | ★ | ★ | ★ | 8 |
| Fernandez Casares[36] | ★ | ★ | ★ | ★ | ★★ | ★ | ★ | ★ | 9 |
| Tzelepis[12] | ★ | ★ | ★ | ★ | ☆☆ | ★ | ★ | ★ | 7 |
| Matsuda[40] | ★ | ★ | ★ | ★ | ★☆ | ★ | ★ | ☆ | 7 |
| Namba[42] | ★ | ☆ | ☆ | ☆ | ★★ | ★ | ★ | ★ | 6 |
| Jiang[33] | ★ | ★ | ★ | ★ | ★★ | ☆ | ★ | ★ | 8 |

**Supplement C** Study quality of cross-sectional study.

| **Author** | **Define the source of information** | **List inclusion and exclusion criteria for exposed and unexposed subjects or refer to previous publications** | **Indicate time period used for identifying patients** | **Indicate whether or not subjects were consecutive if not population-based** | **Indicate if evaluators of subjective components of study were masked to other aspects of the status of the participants** | **Describe any assessments undertaken for quality assurance purposes** | **Explain any patient exclusions from analysis** | **Describe how confounding was assessed and/or controlled** | **If applicable, explain how missing data were handled in the analysis** | **Summarize patient response rates and completeness of data collection** | **Clarify what follow-up, if any, was expected and the percentage of patients for which incomplete data or follow-up was obtained** | **Total scores** |
| --- | --- | --- | --- | --- | --- | --- | --- | --- | --- | --- | --- | --- |
| Tan[30] | Yes | Yes | No | Yes | Unclear | Yes | No | Unclear | Unclear | No | Unclear | 4 |
| Liu[32] | Yes | Yes | No | Yes | Unclear | Yes | No | Unclear | Unclear | No | Unclear | 4 |
| Conticini [37] | Yes | Yes | Yes | Yes | Yes | No | No | Yes | Unclear | Yes | Yes | 8 |
| Matsuda [19] | Yes | Yes | Yes | No | Yes | Yes | No | No | Unclear | Yes | Unclear | 6 |
| Matsuda [39] | Yes | Yes | Yes | No | Yes | Yes | No | No | Unclear | Yes | Unclear | 6 |
| Flores-Suárez[43] | Yes | Yes | Yes | Yes | Yes | Yes | No | Yes | Unclear | Yes | Unclear | 8 |
| Iwata[18] | Yes | Yes | No | Yes | Yes | Yes | Yes | No | Unclear | Yes | Unclear | 7 |

**Supplement D** Forest plots with risk factor in AAV-ILD

**Figure 2** Forest plots of WMDs for the correlation age with AAV-ILD.


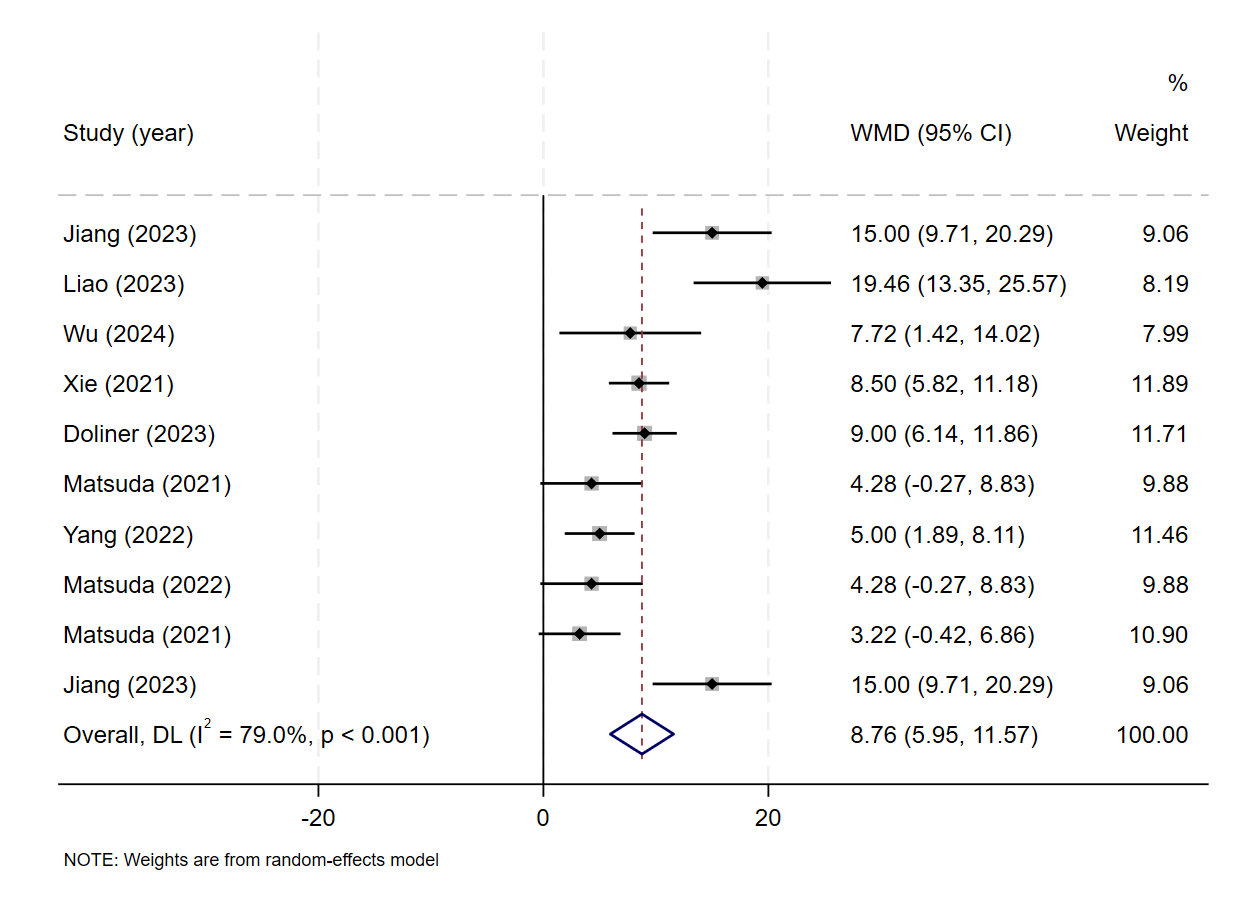


**Figure 3** Forest plots of ORs for the correlation male with AAV-ILD.


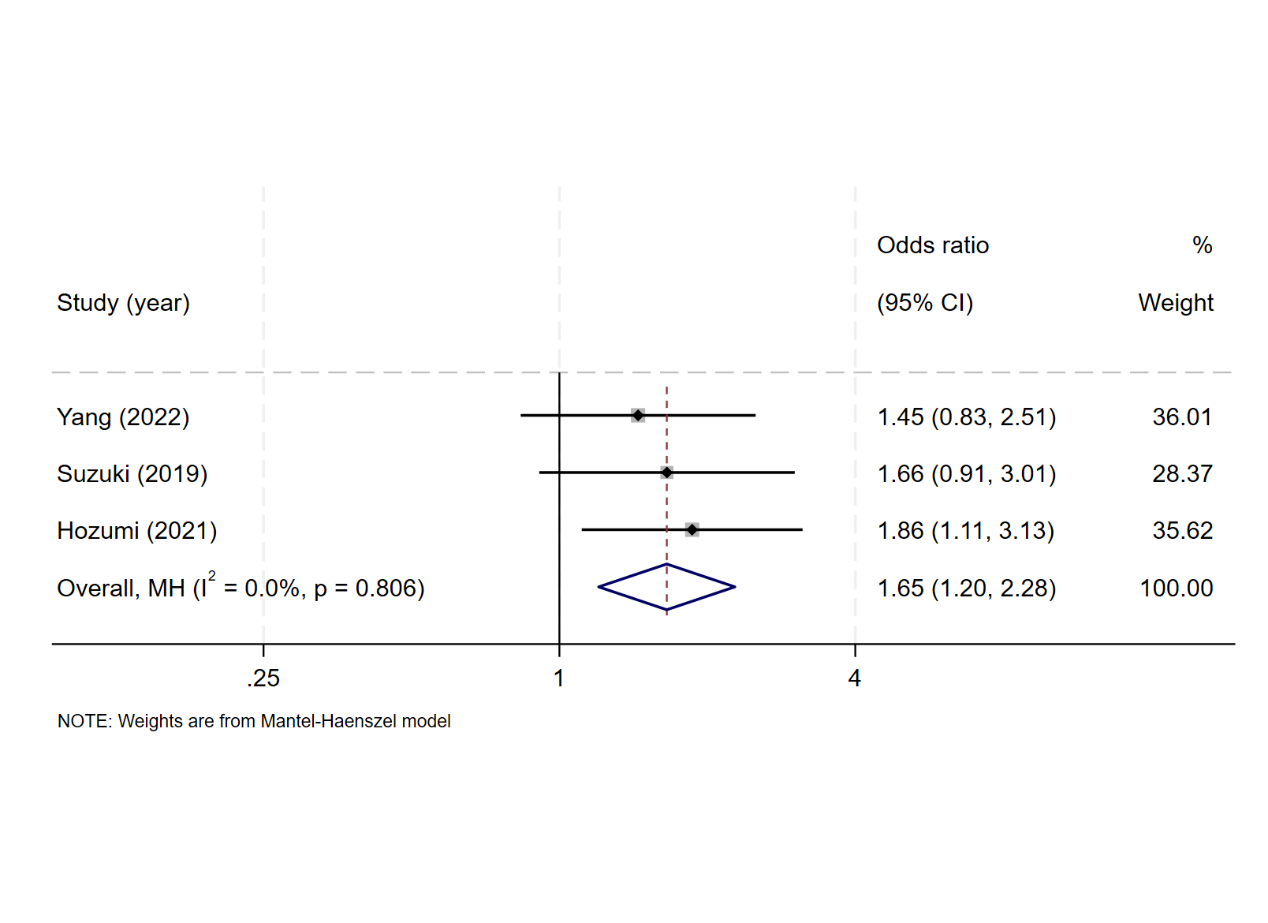


**Figure 4** Forest plots of ORs for the correlation smoking with AAV-ILD.


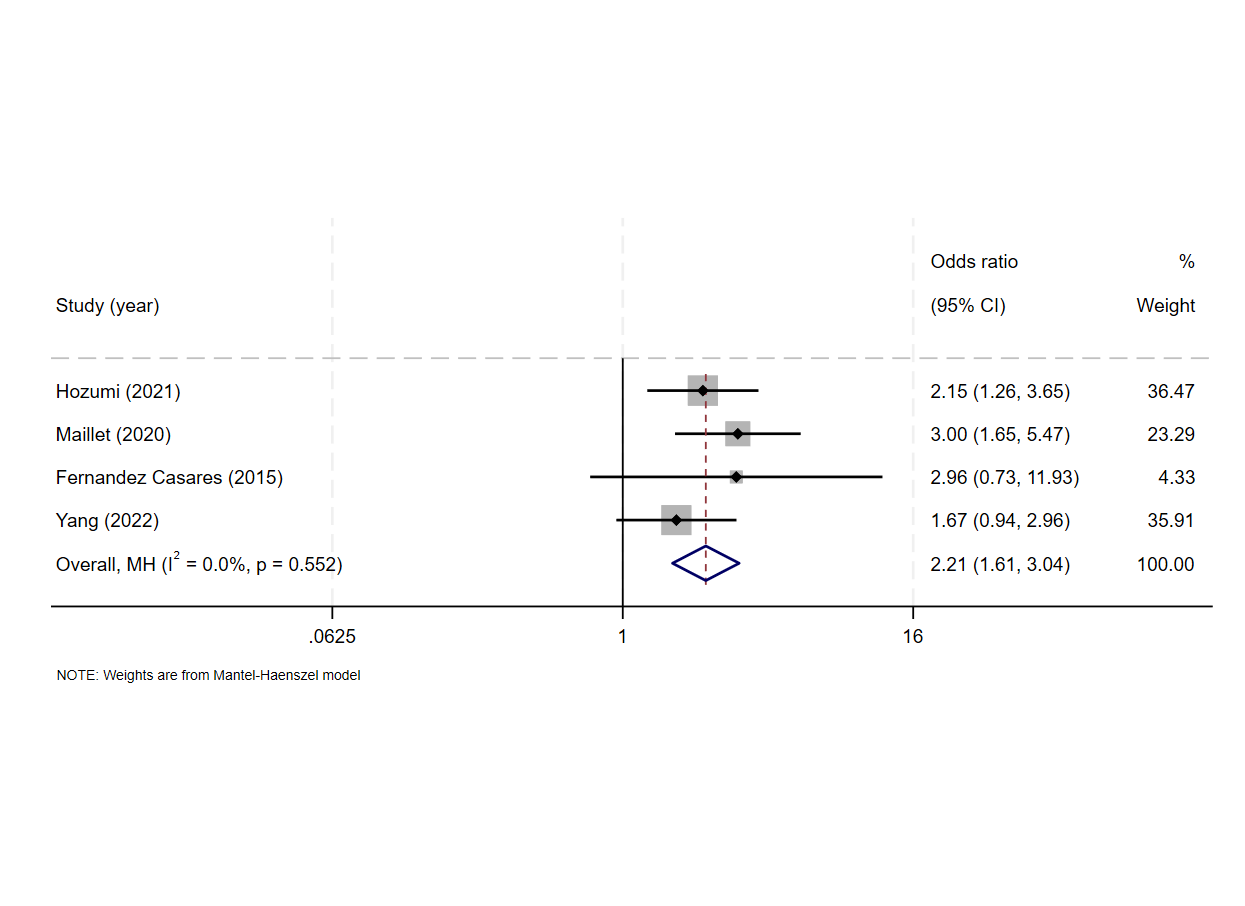


**Figure 5** Forest plots of ORs for the correlation honeycombing with AAV-ILD.


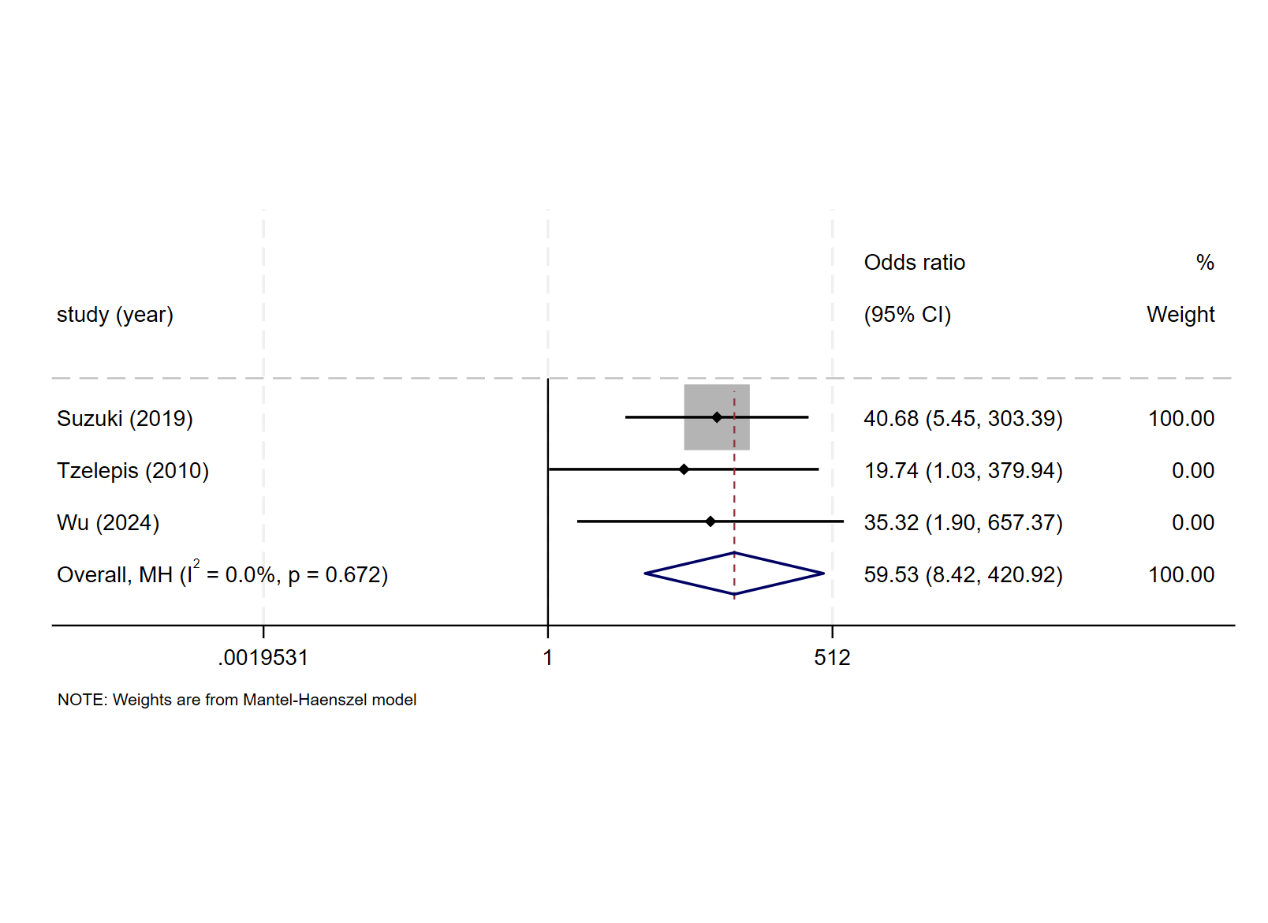


**Figure 6** Forest plots of ORs for the correlation lattice shadows with AAV-ILD.


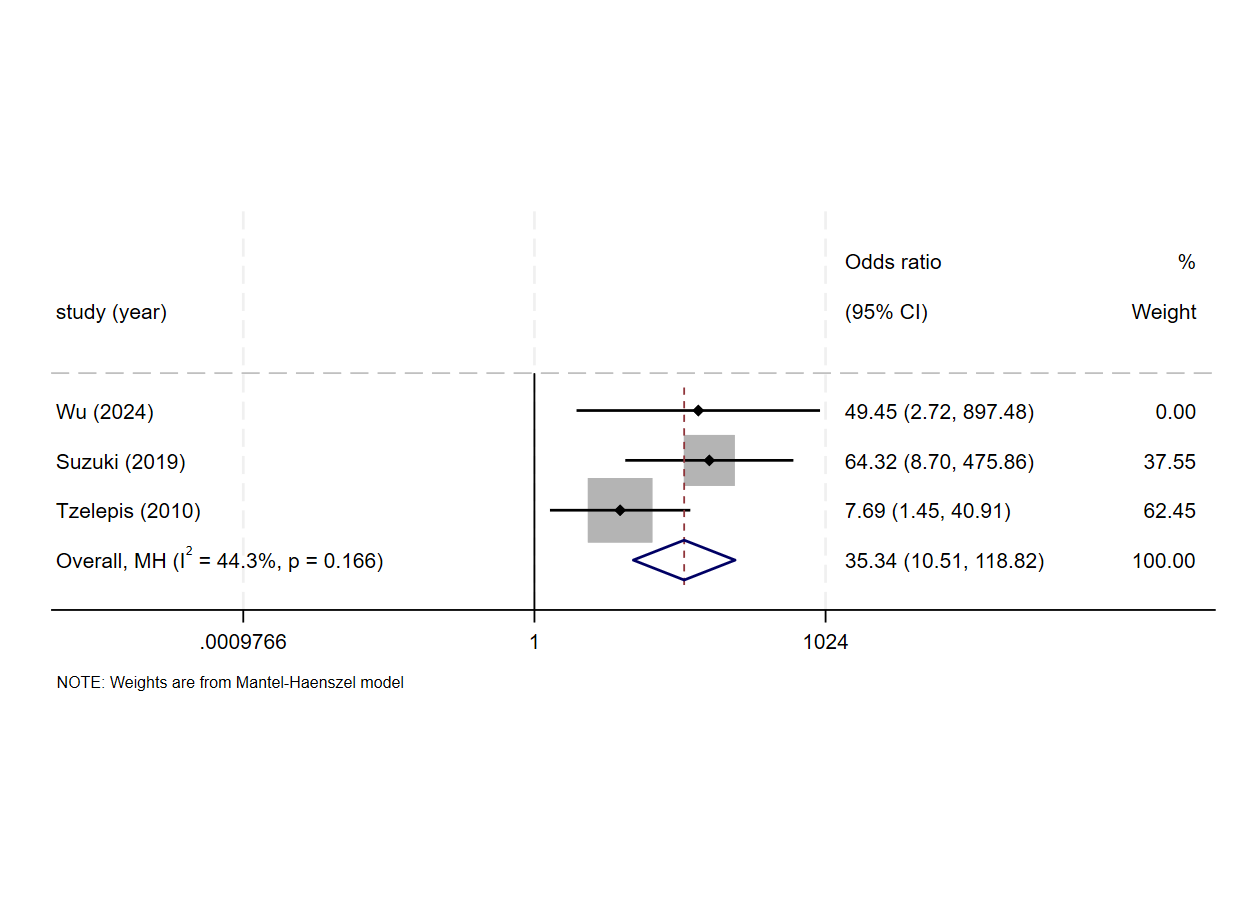


**Figure 7** Forest plots of ORs for the correlation interlobular septal thickening with AAV-ILD.


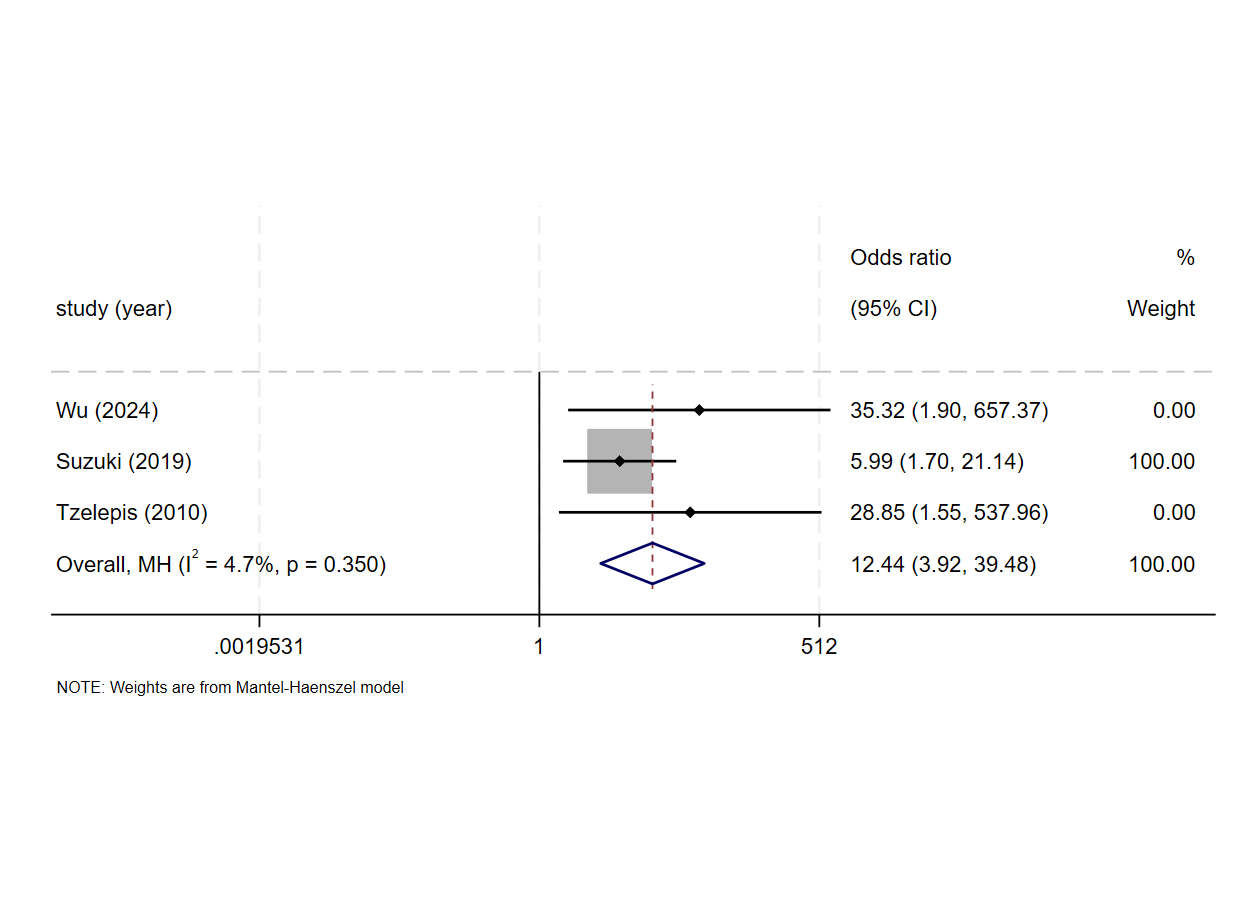


**Figure 8** Forest plots of ORs for the correlation MPO-ANCA with AAV-ILD.


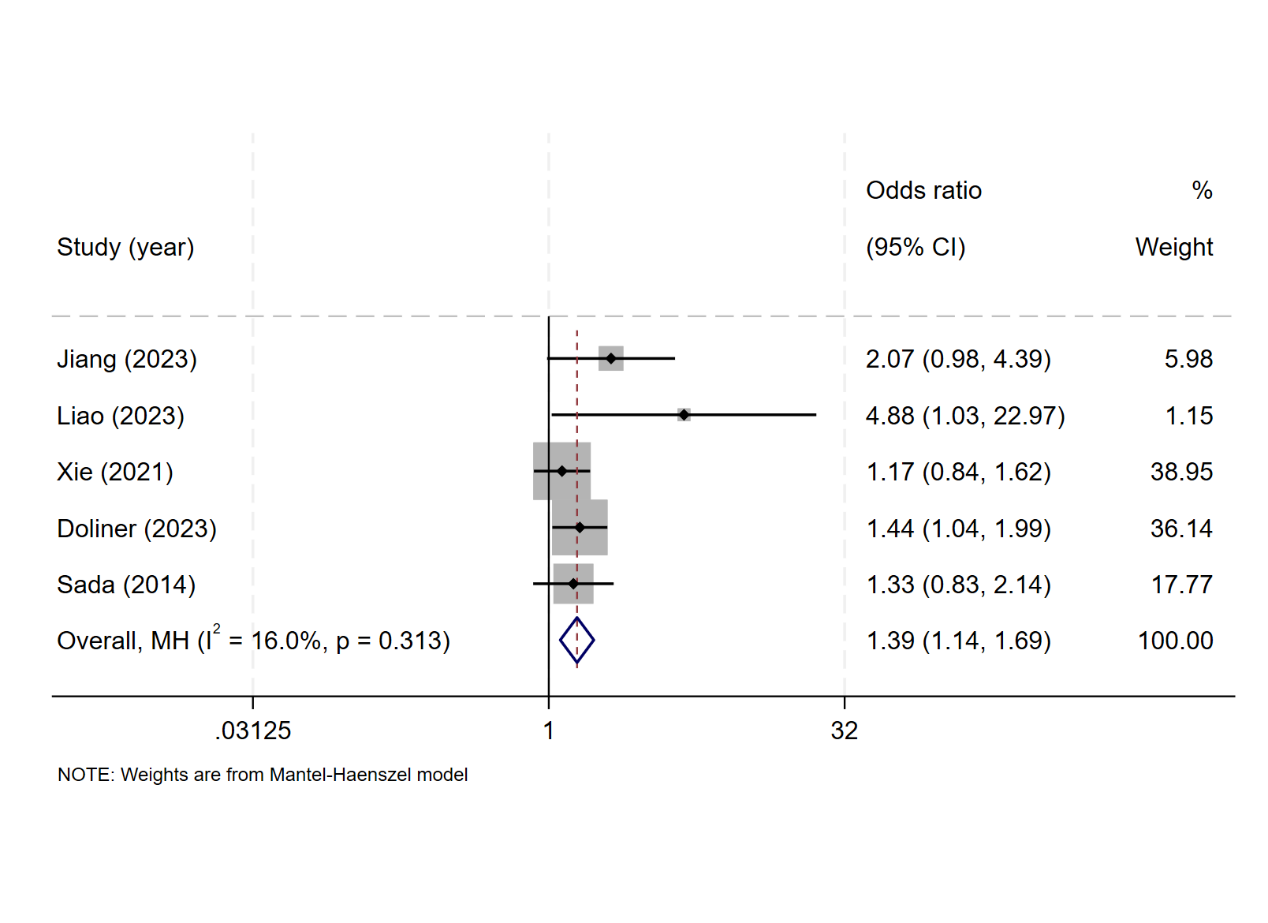


**Figure 9** Forest plots of WMDs for the correlation ESR with AAV-ILD.


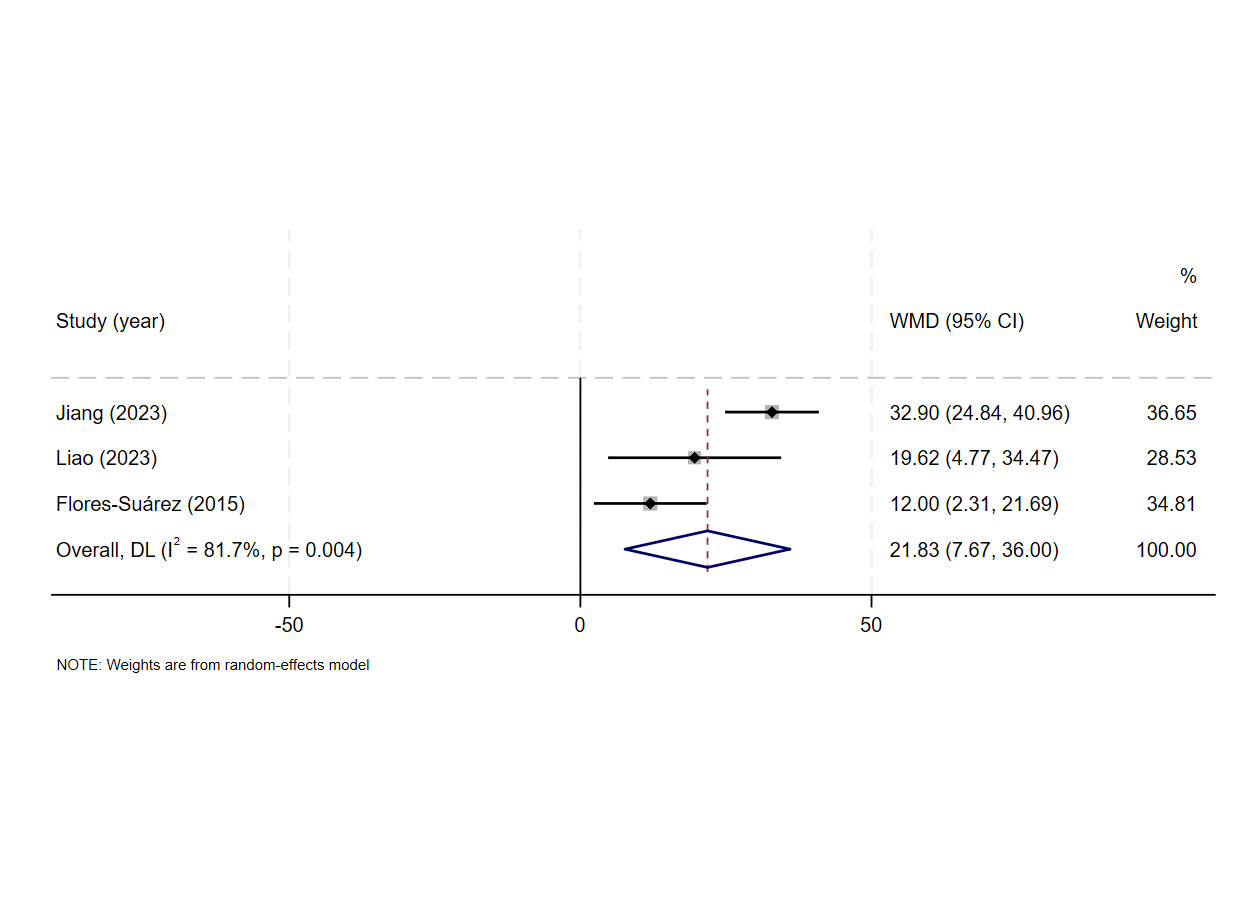


**Figure 10** Forest plots of WMDs for the correlation KL-6 with AAV-ILD.


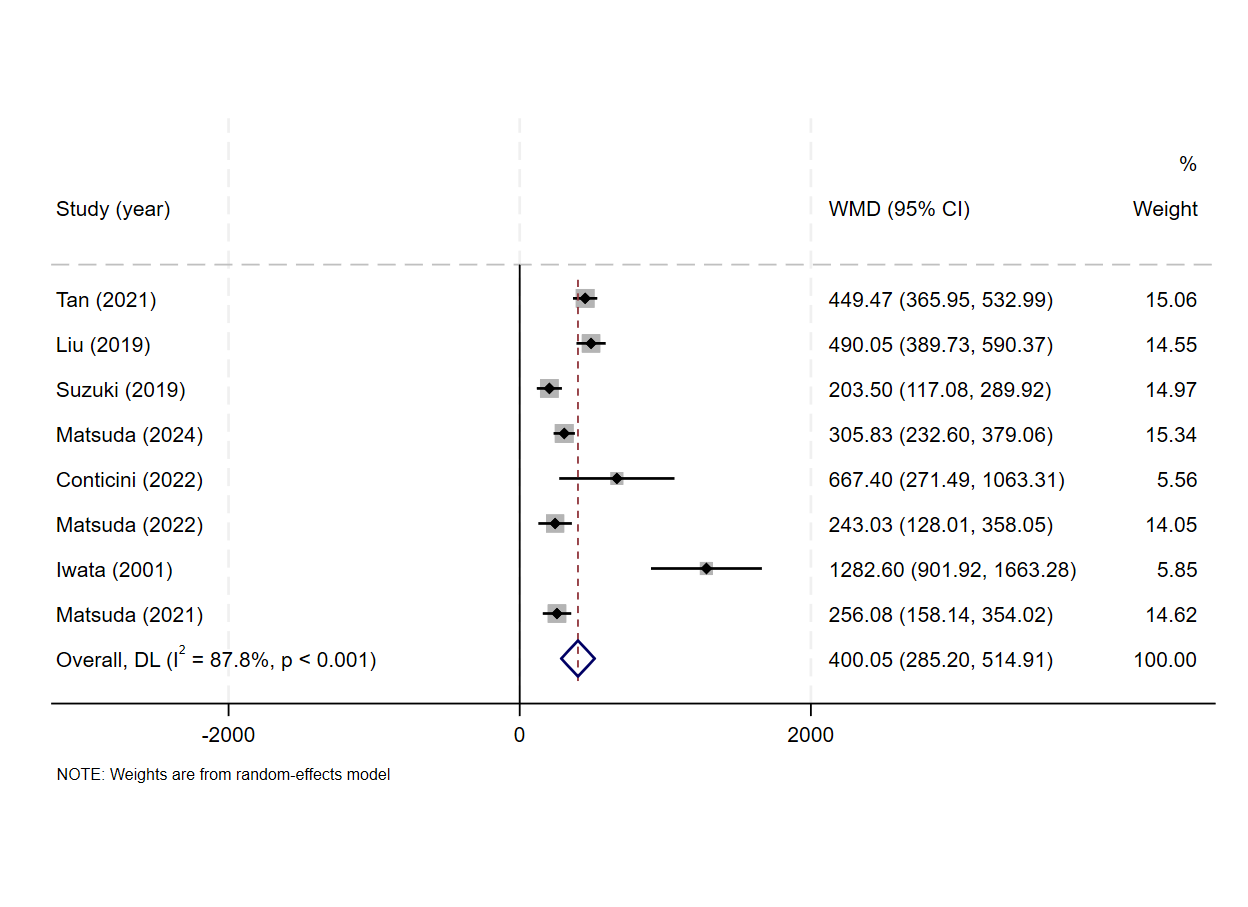


**Figure 11** Forest plots of WMDs for the correlation Hb with AAV-ILD.


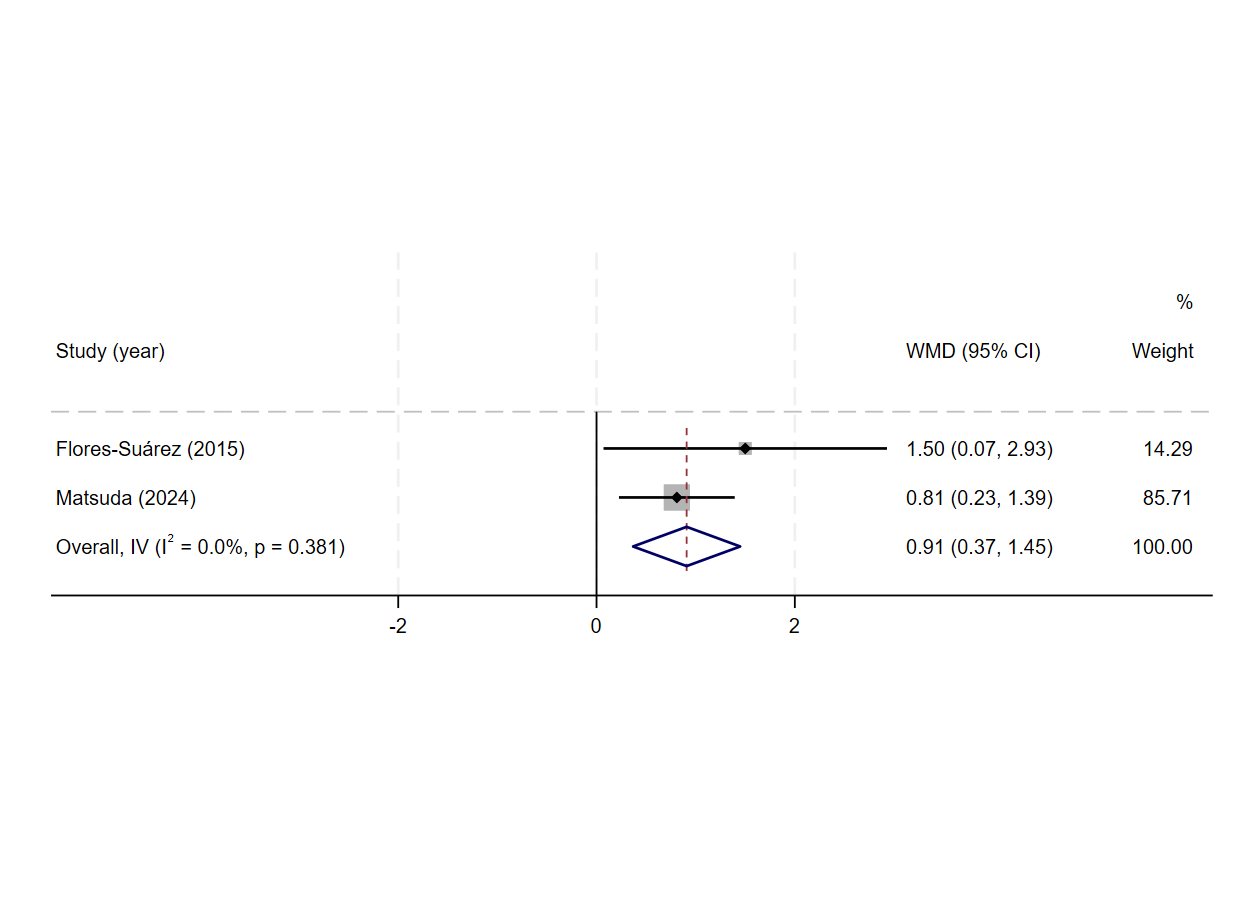


**Figure 12** Forest plots of WMDs for the correlation BVAS with AAV-ILD.


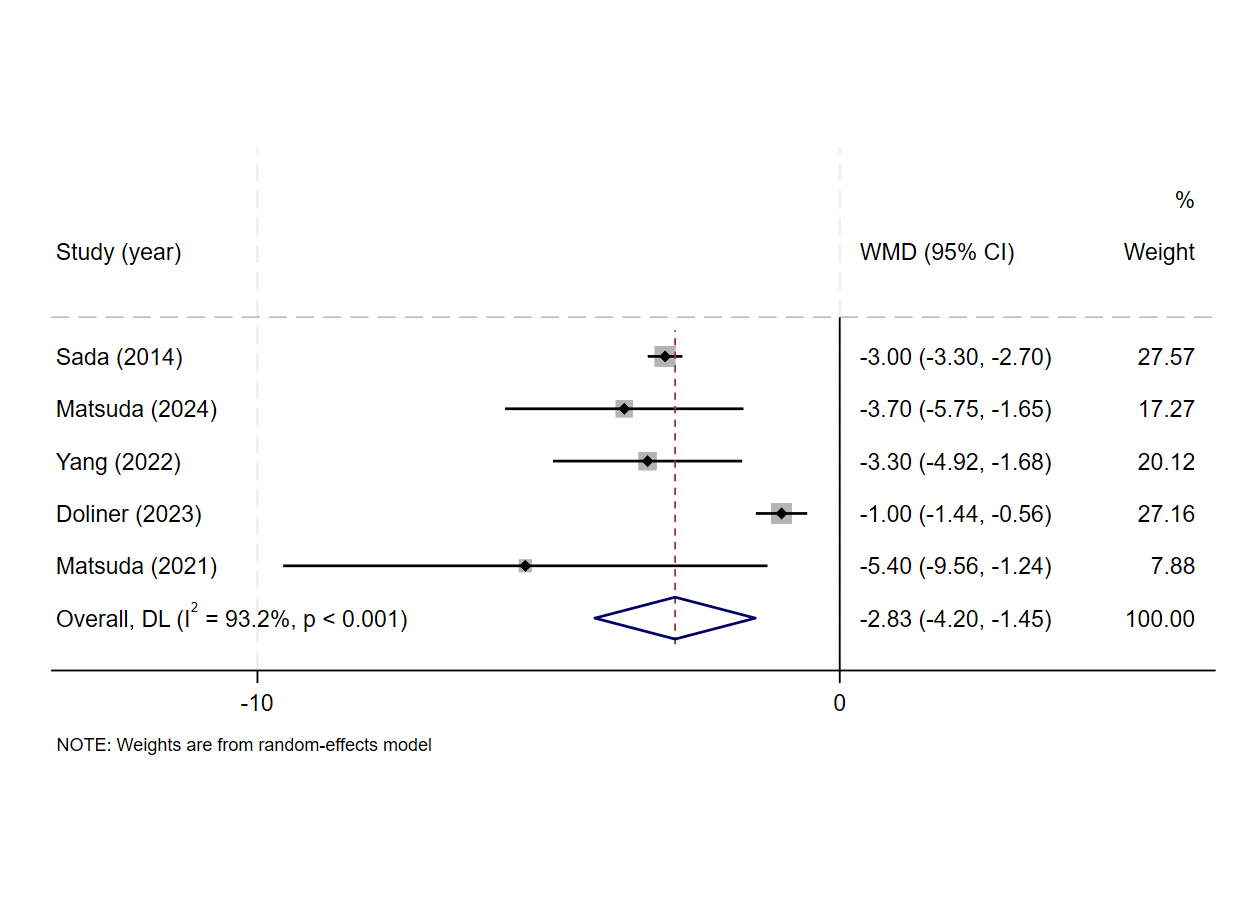


**Figure 13** Forest plots of ORs for the correlation Ear, nose & throat with AAV-ILD.


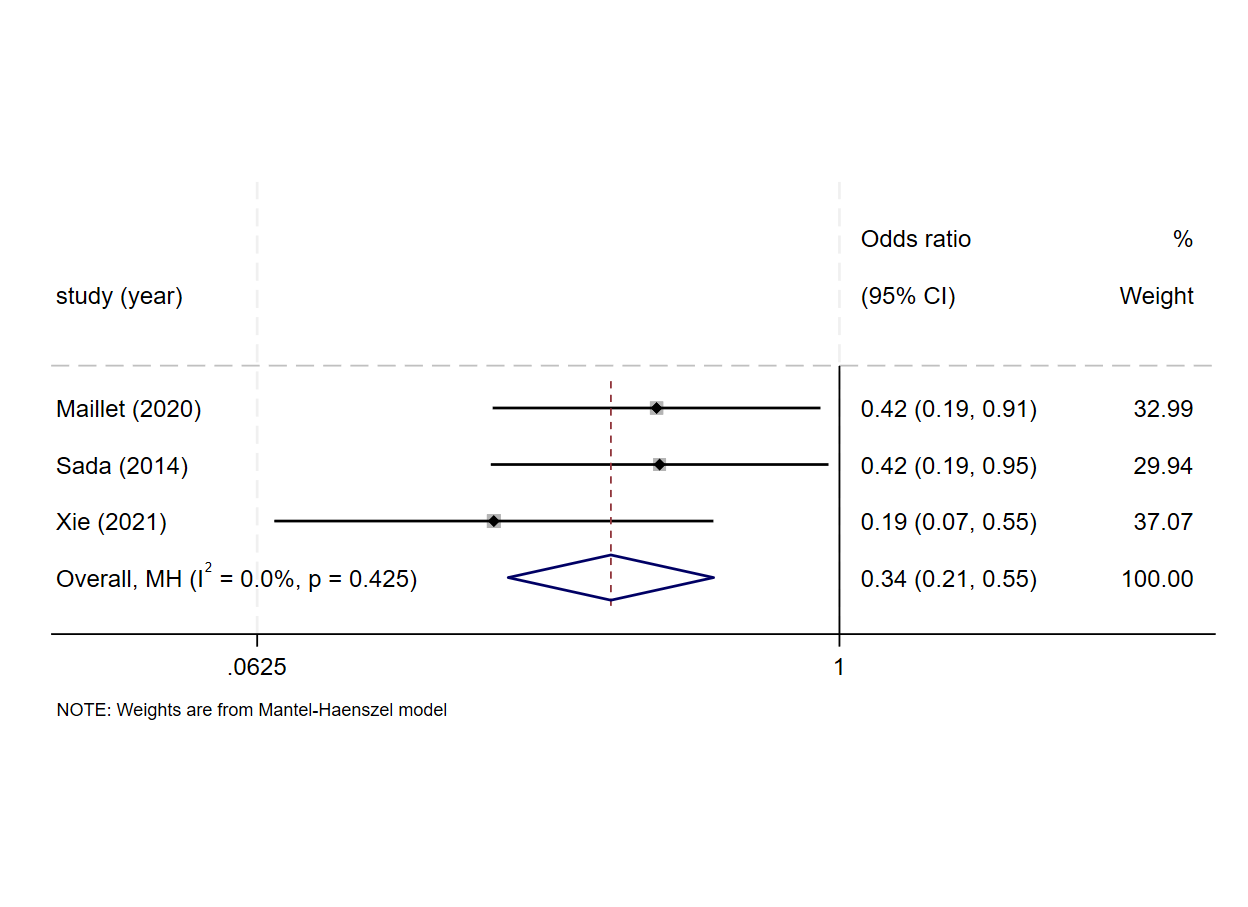


**Figure 14** Forest plots of ORs for the correlation fever with AAV-ILD.


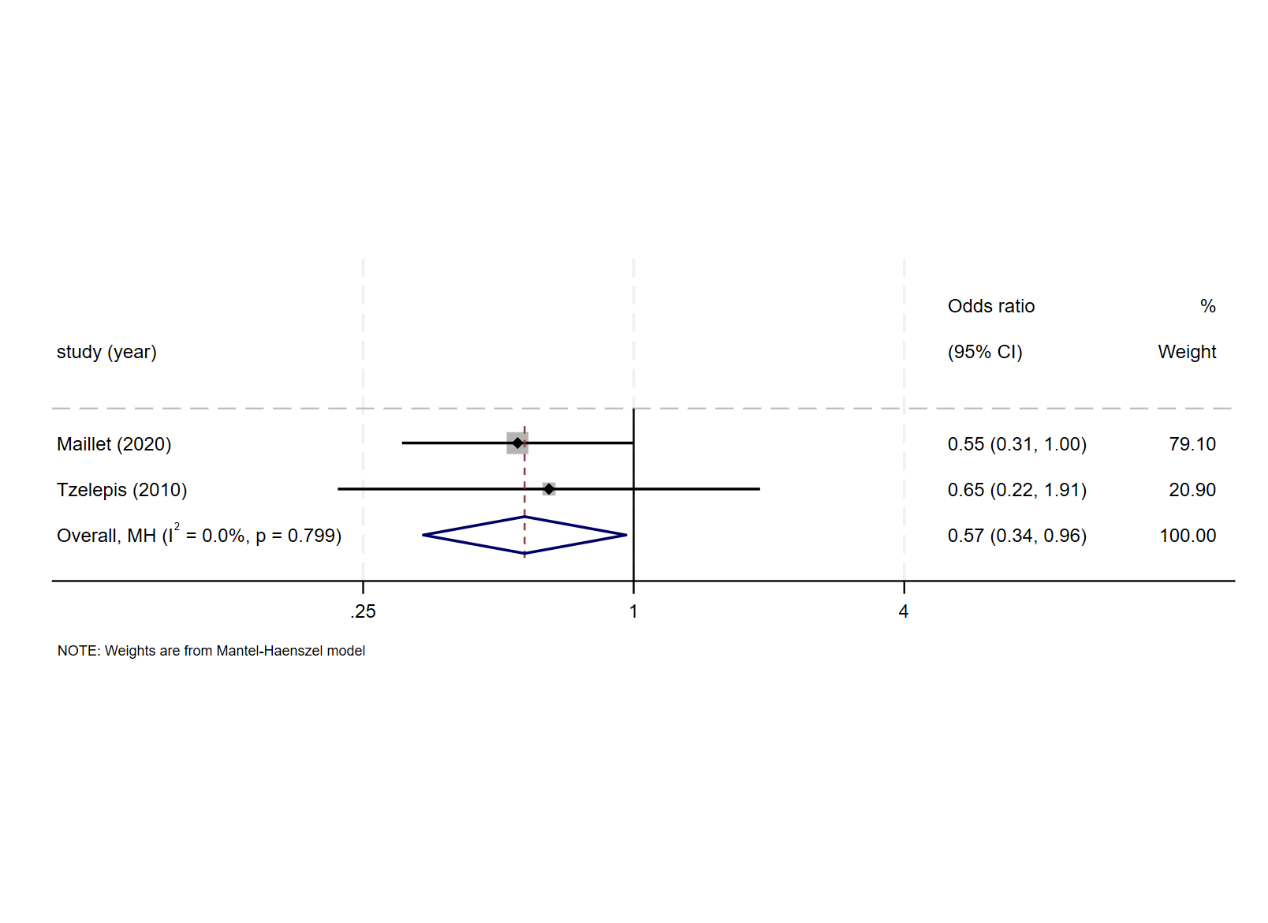


**Figure 15** Forest plots of ORs for the correlation dyspnea with AAV-ILD.


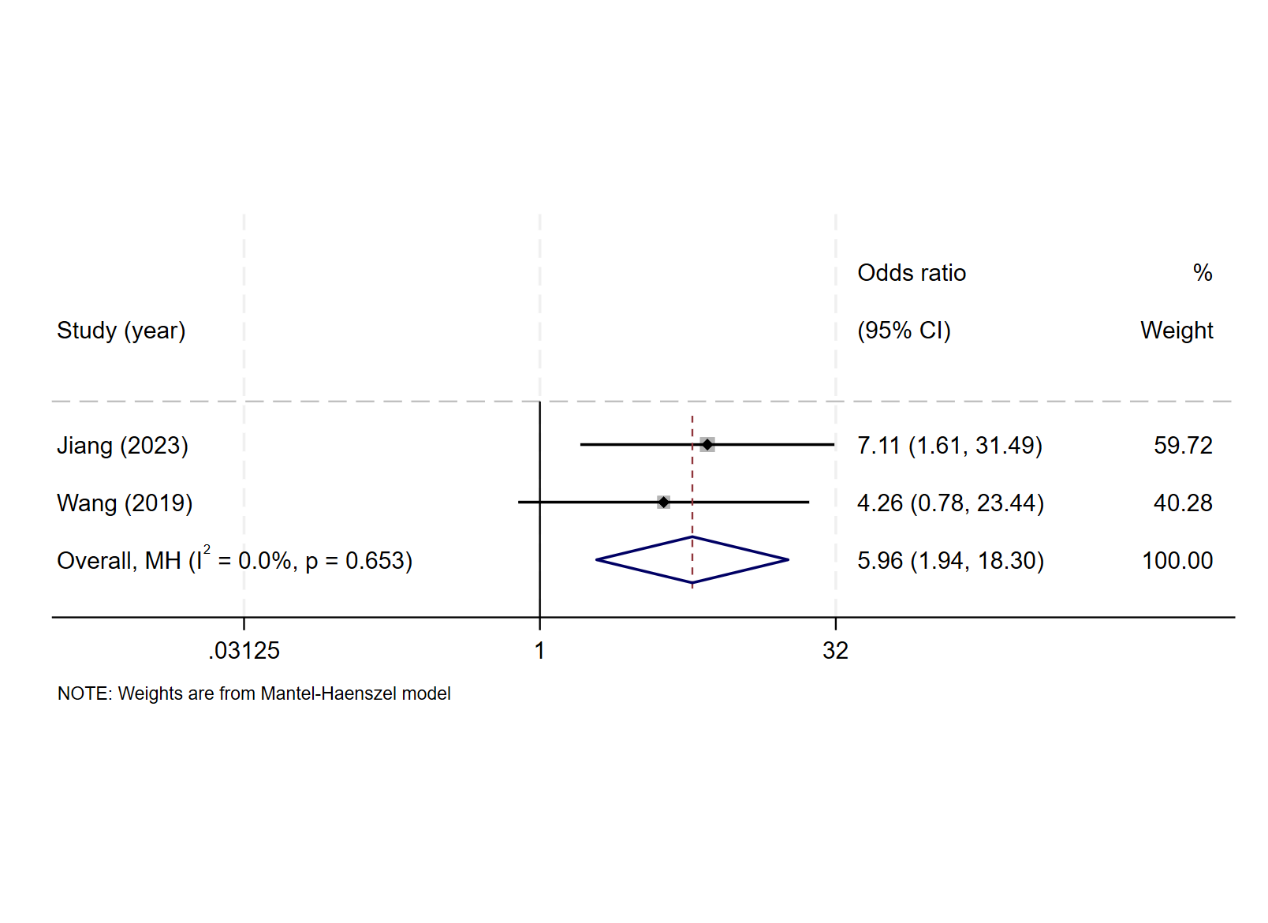


**Figure 16** Forest plots of ORs for the correlation of cough with AAV-ILD.


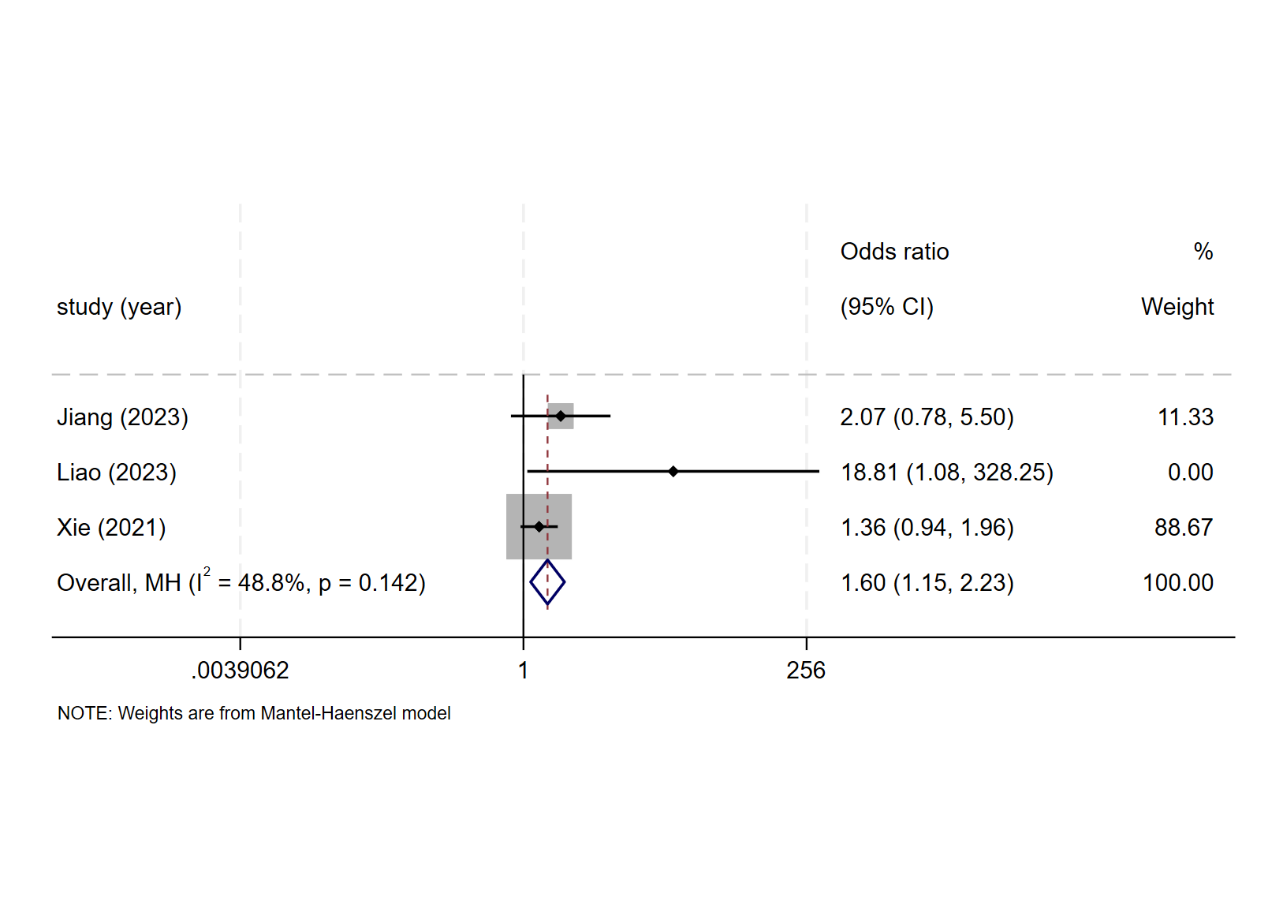


**Figure 17** Forest plots of WMDs for the correlation alb with AAV-ILD.


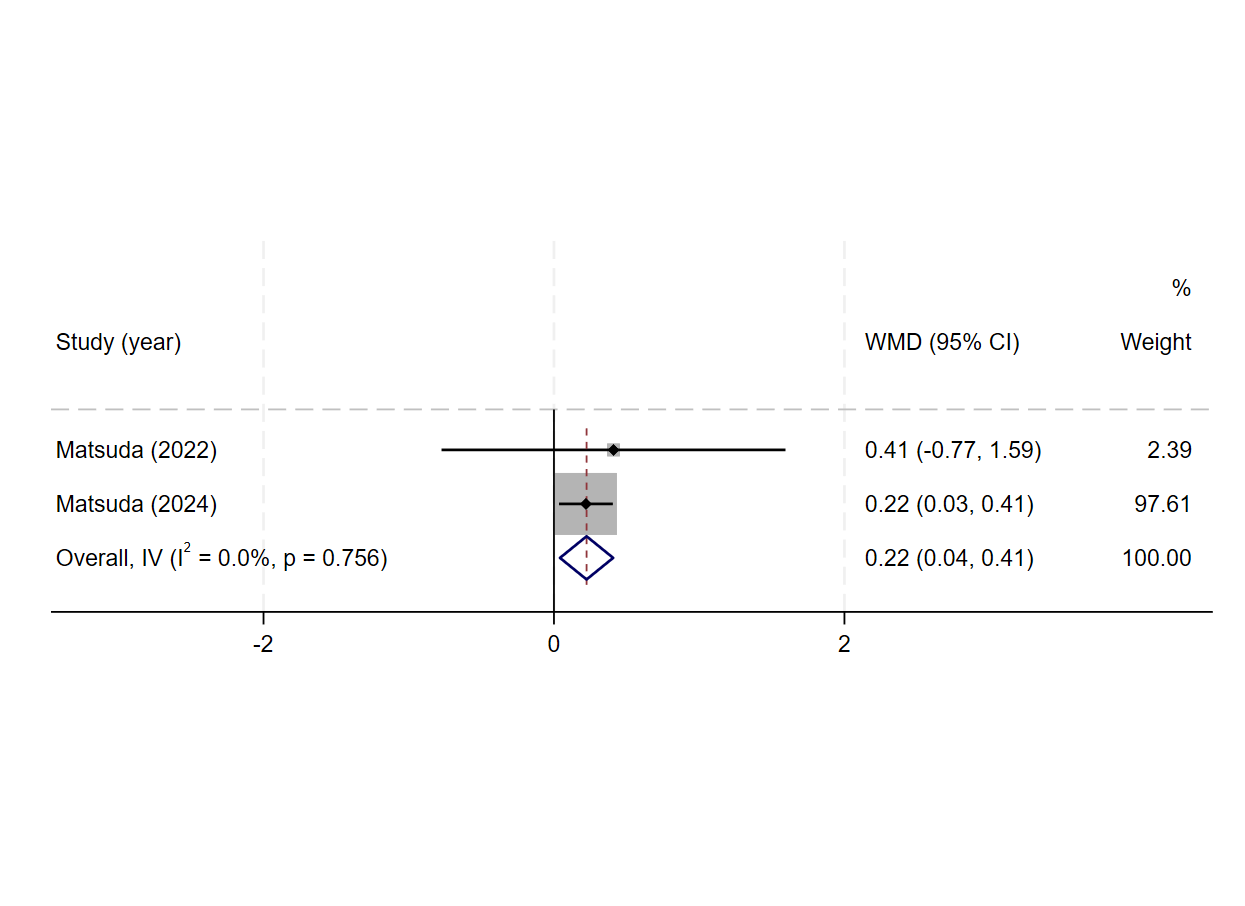


**Figure 18** Forest plots of ORs for the correlation pulmonary comorbidities with AAV-ILD.


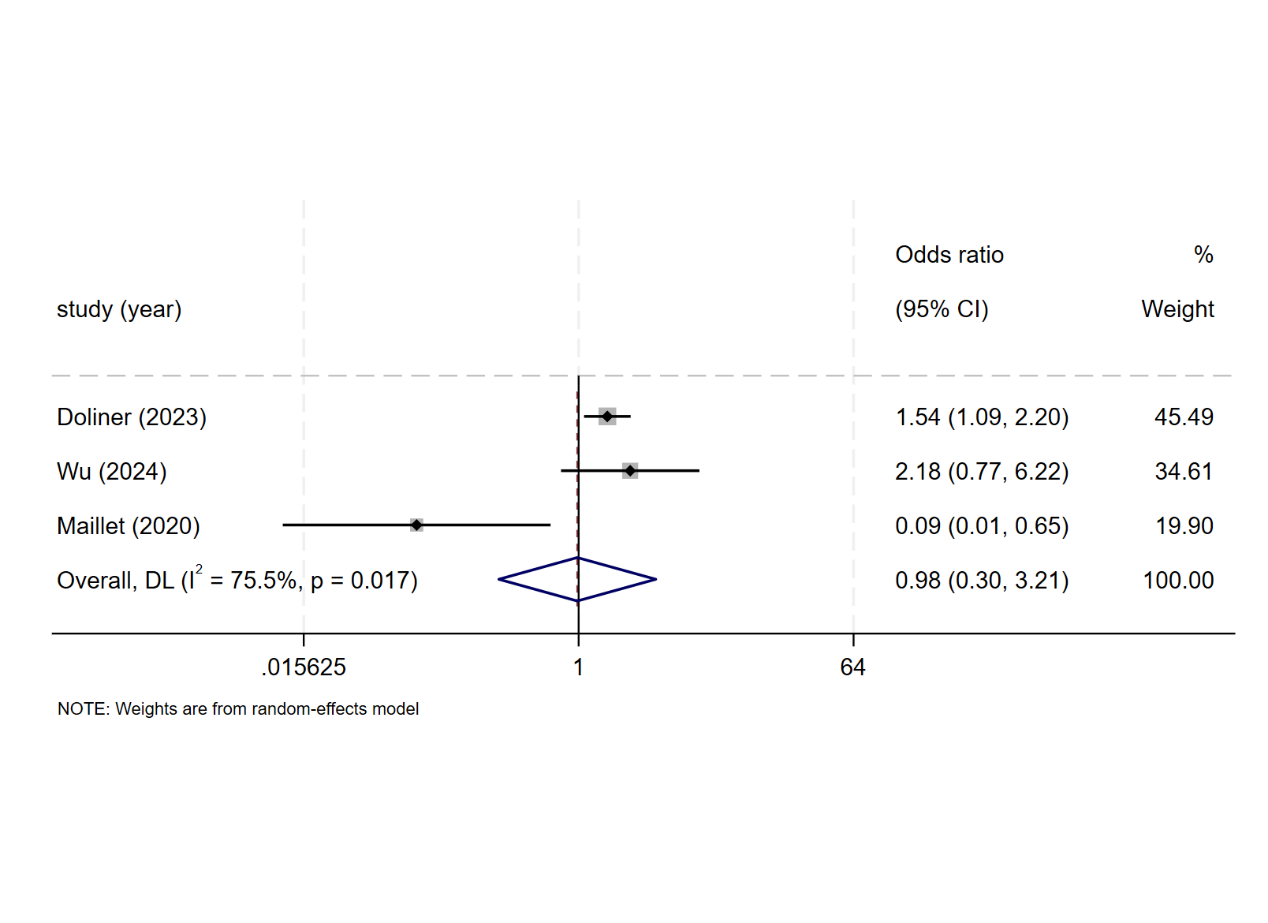


**Figure 19** Forest plots of ORs for the correlation PR3-ANCA with AAV-ILD.


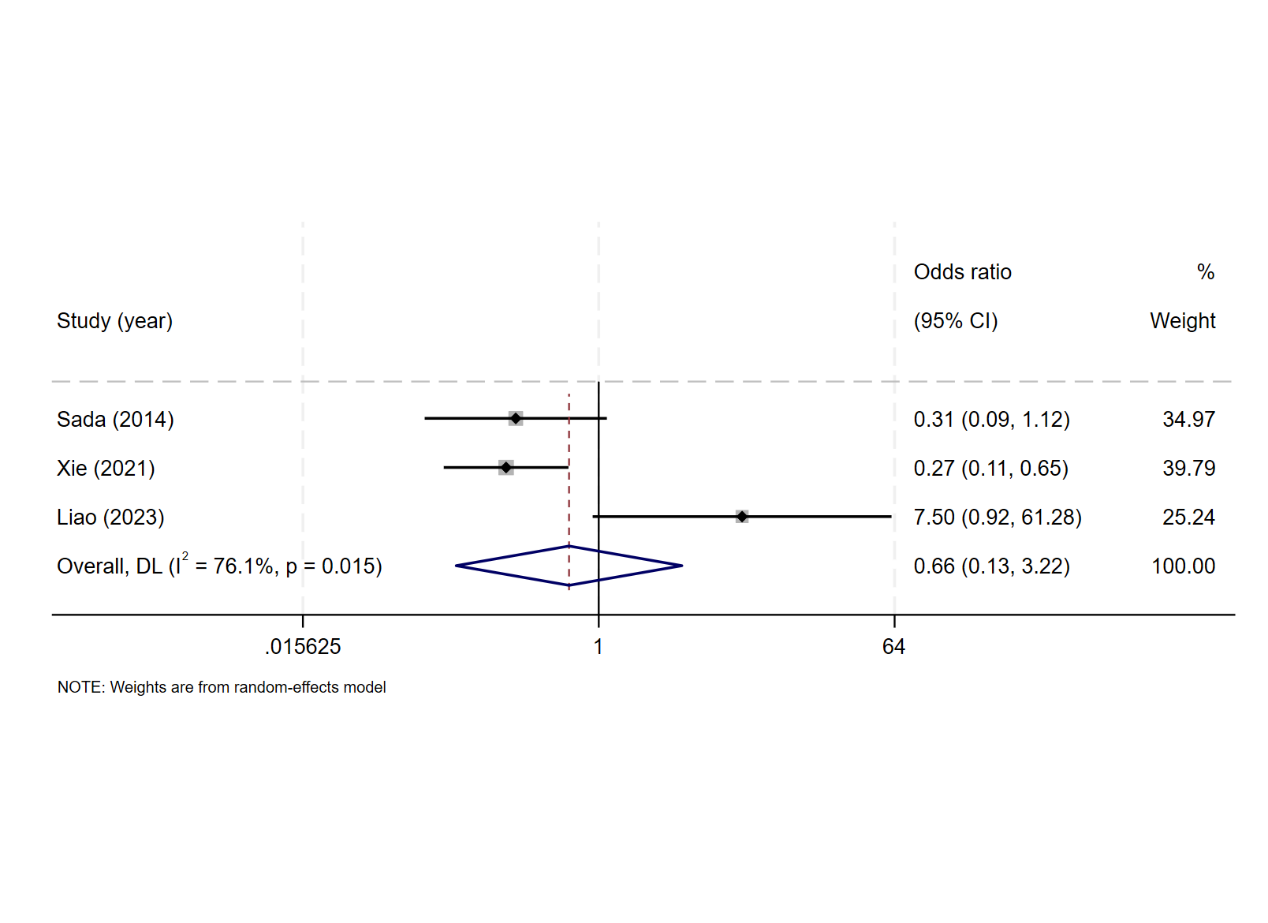


**Figure 20** Forest plots of ORs for the correlation plasma exchange with AAV-ILD.


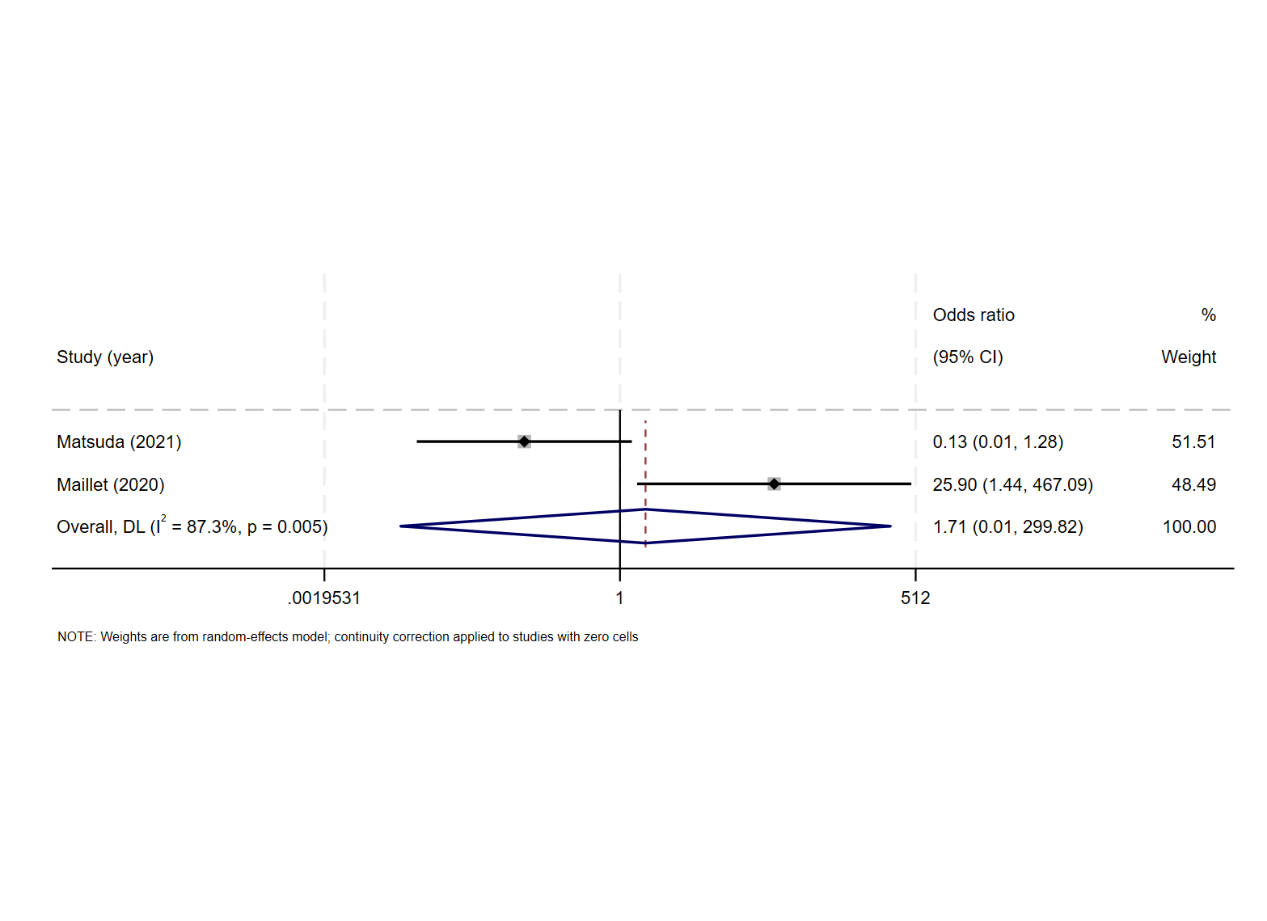


**Figure 21** Forest plots of ORs for the correlation myalgias with AAV-ILD.


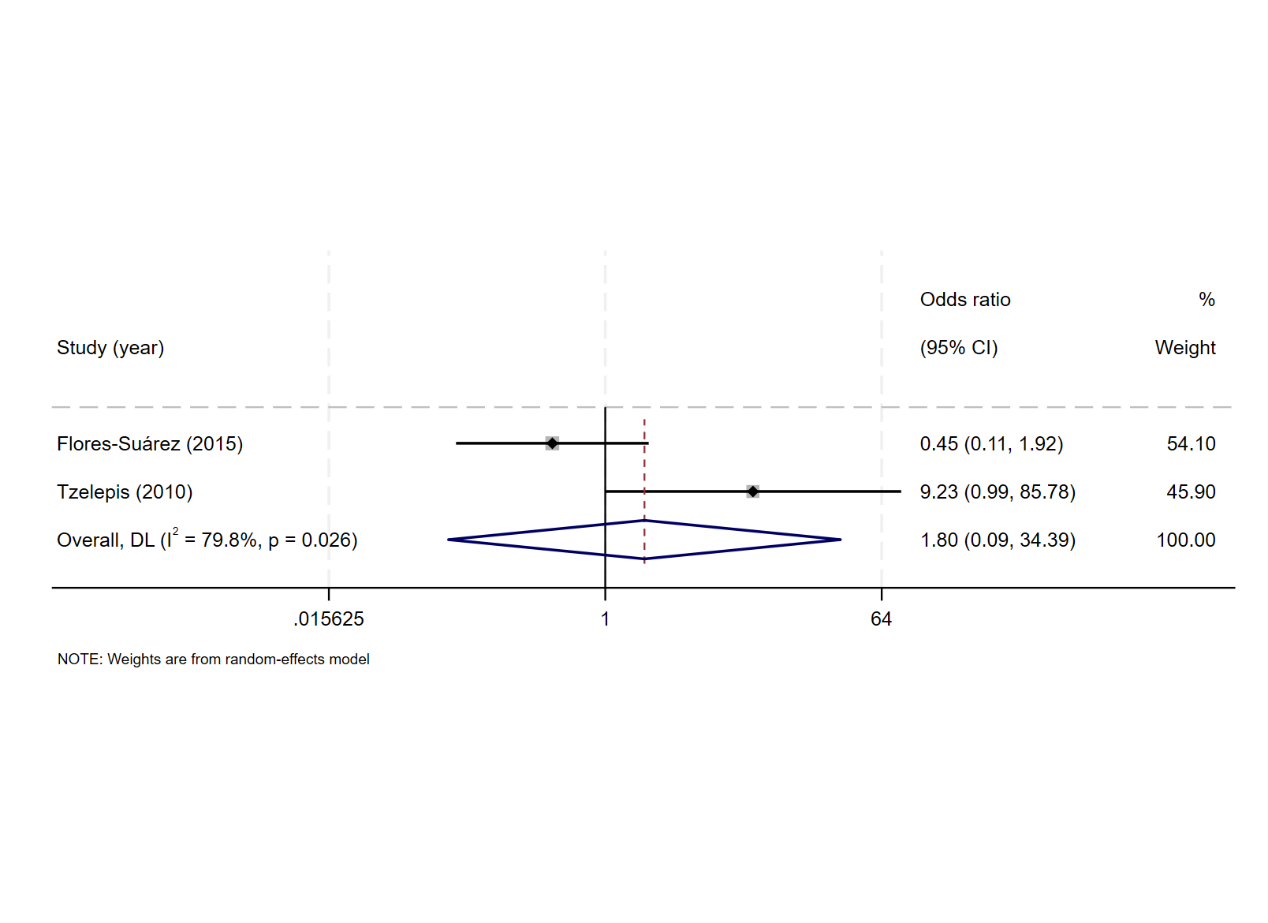


**Figure 22** Forest plots of ORs for the correlation DAH with AAV-ILD.


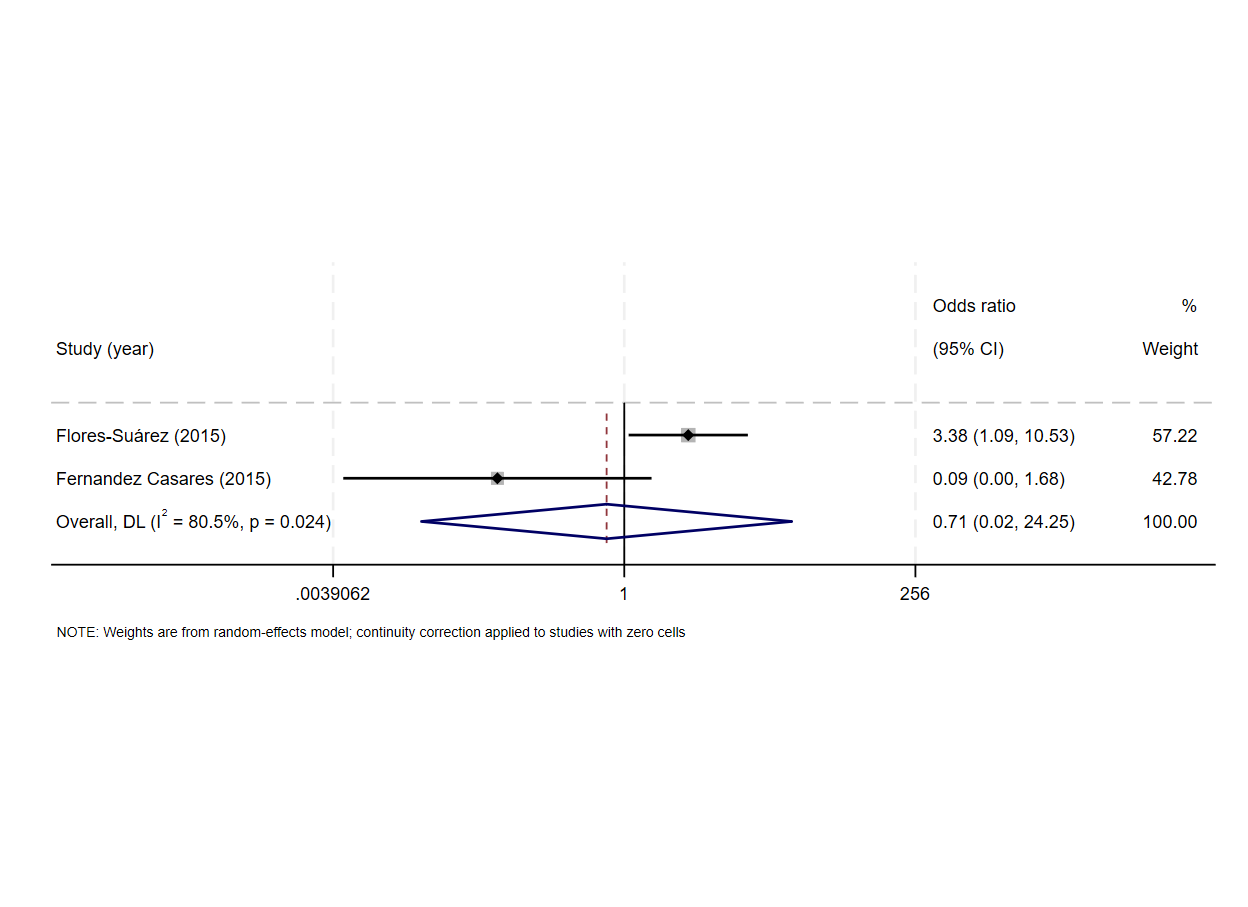


**Figure 23** Forest plots of WMDs for the correlation CRP with AAV-ILD.


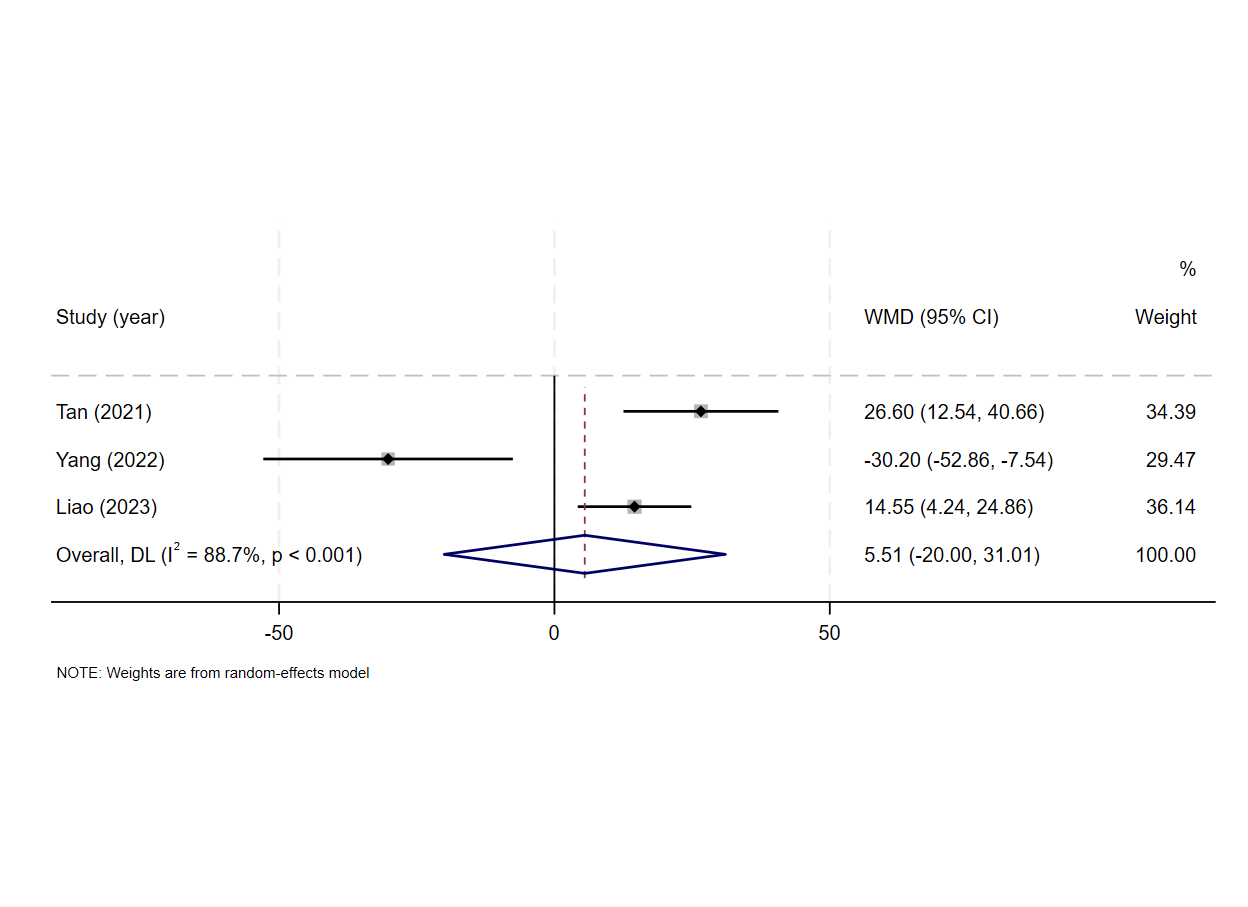


**Figure 24** Forest plots of WMDs for the correlation Cr with AAV-ILD.


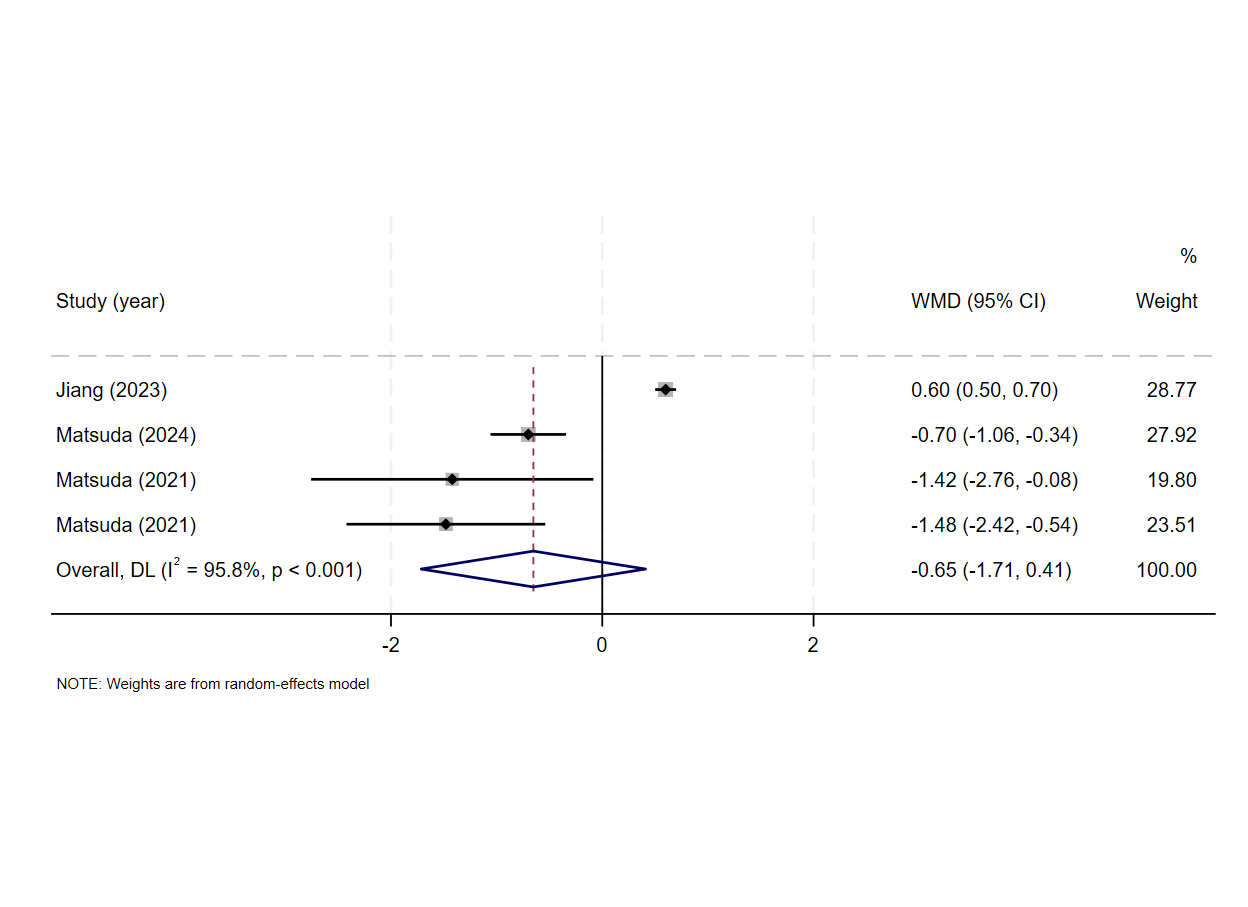


**Figure 25** Forest plots of ORs for the correlation rash with AAV-ILD.


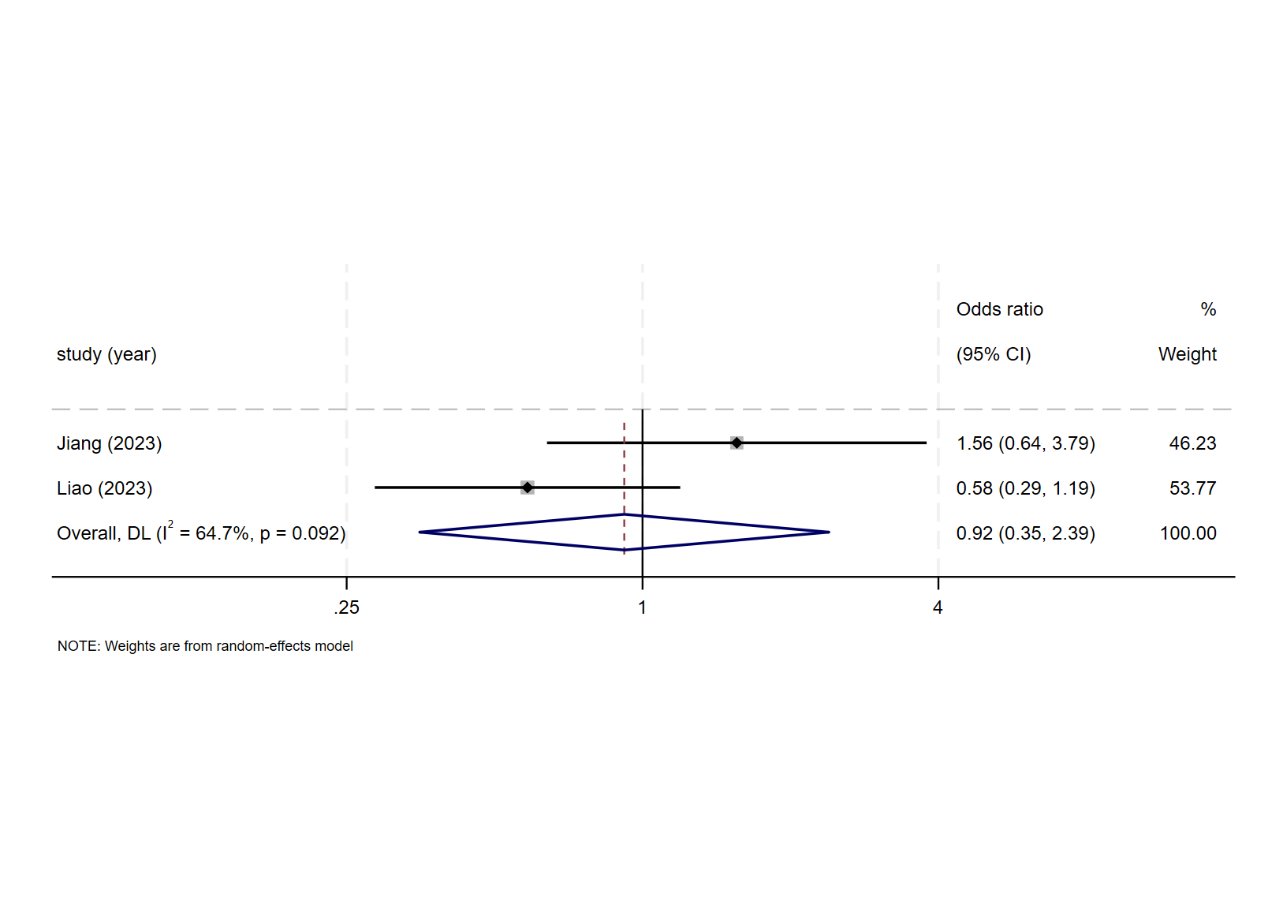


**Figure 26** Forest plots of ORs for the correlation peripheral neuropathy with AAV-ILD.


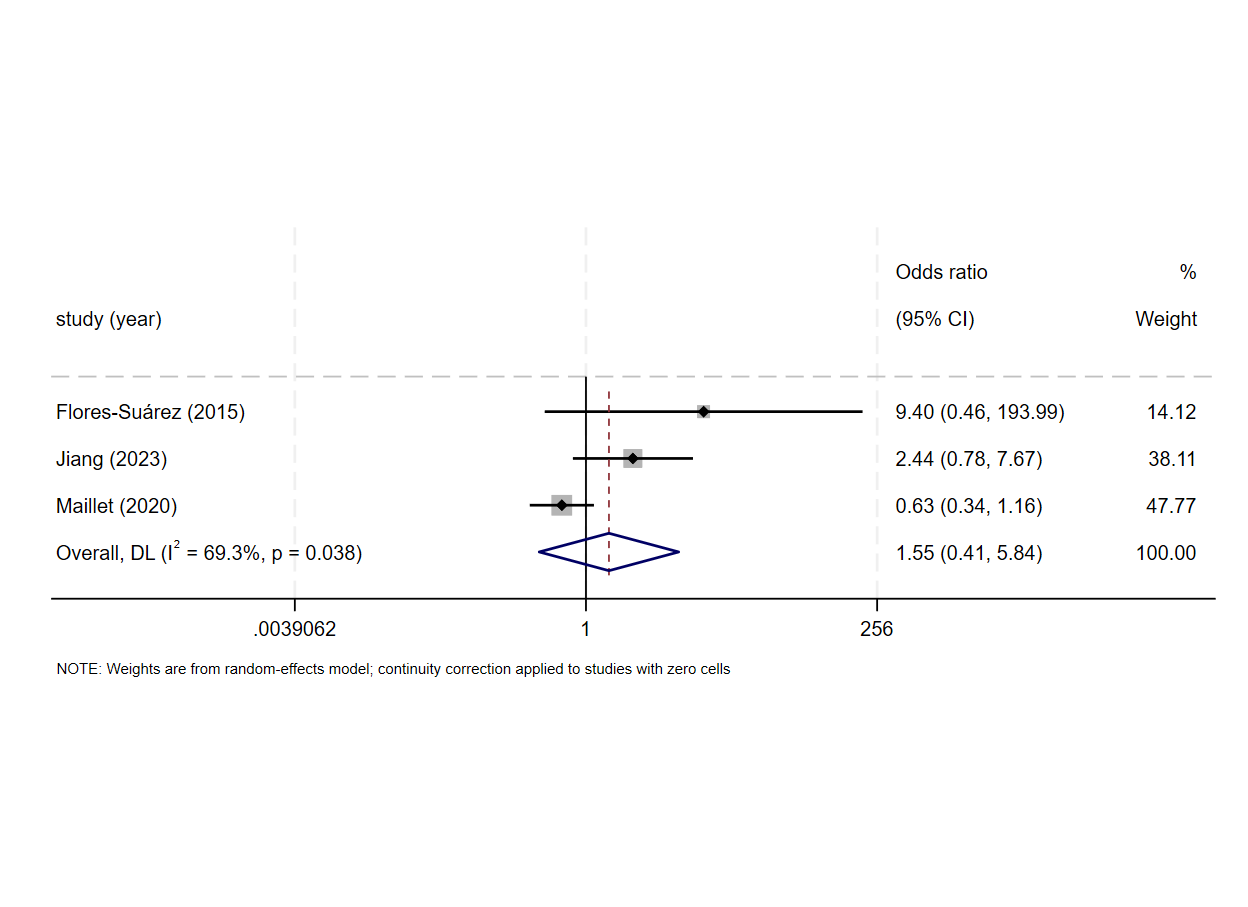


**Figure 27** Forest plots of ORs for the correlation haematuria with AAV-ILD.


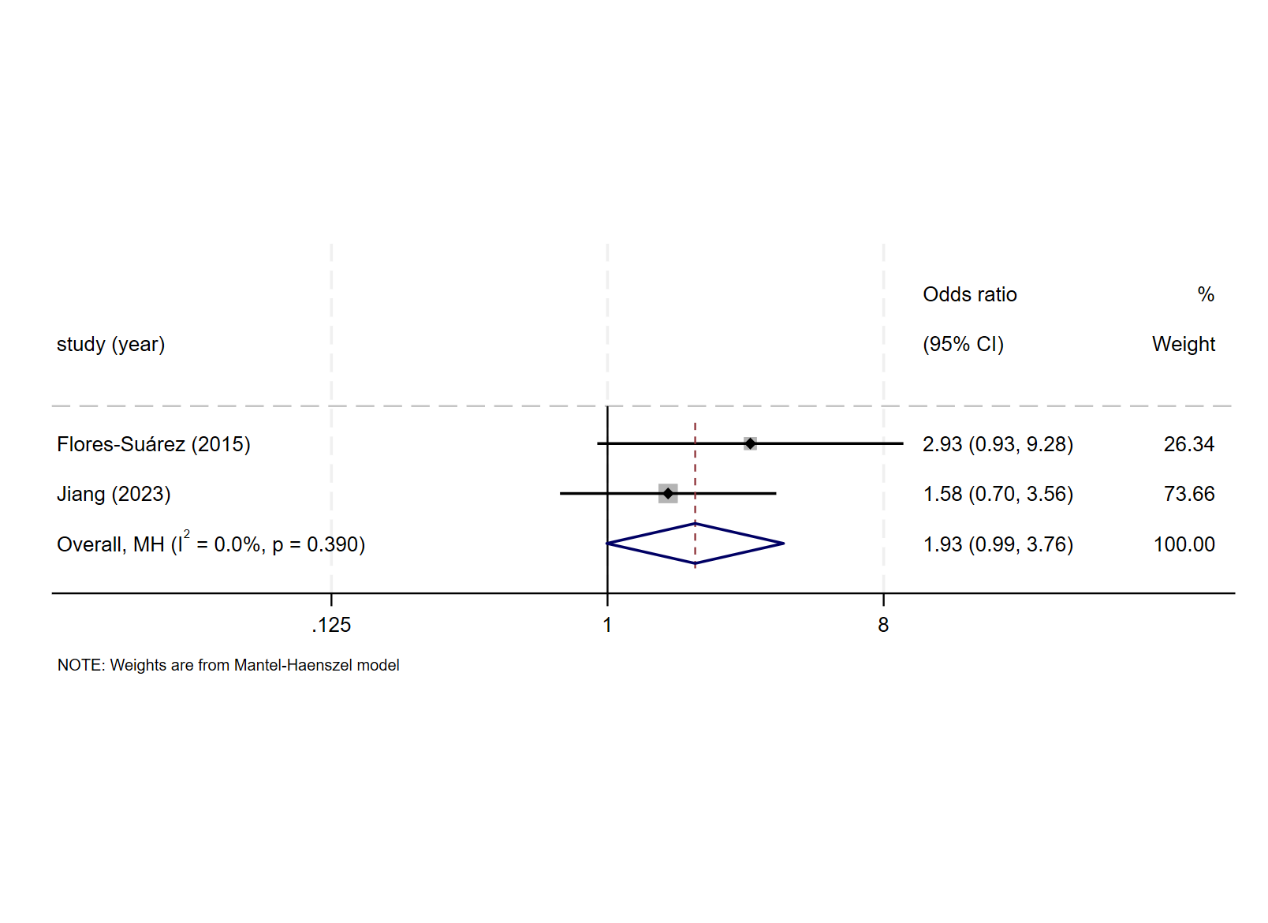


**Figure 28** Egger’s publication bias plot of included studies on age for AAV-ILD.


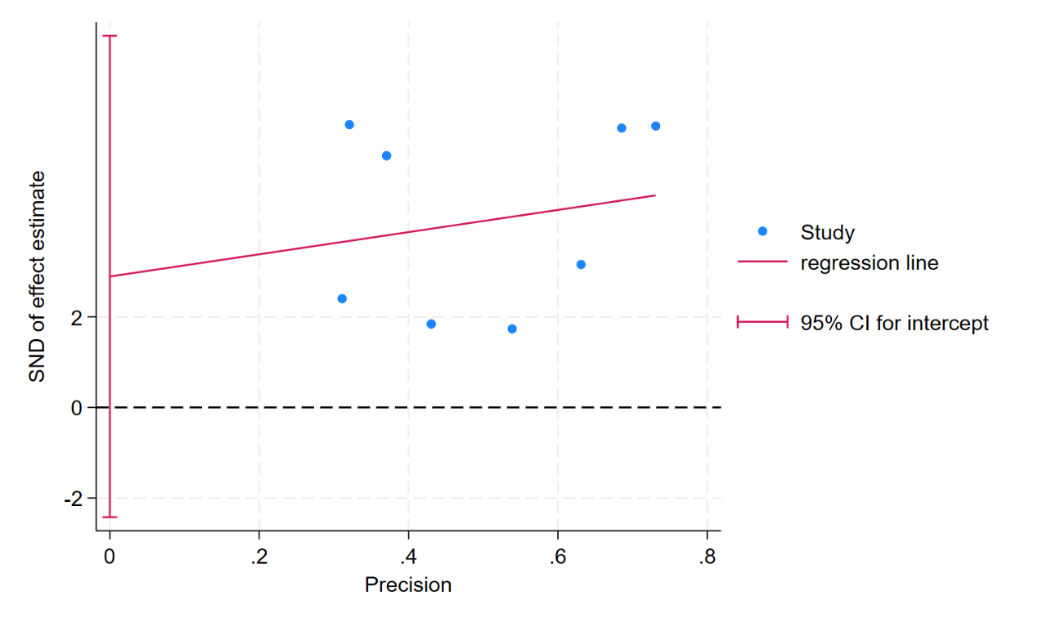


**Figure 29** funnel plot of the age of AAV-ILD.

**
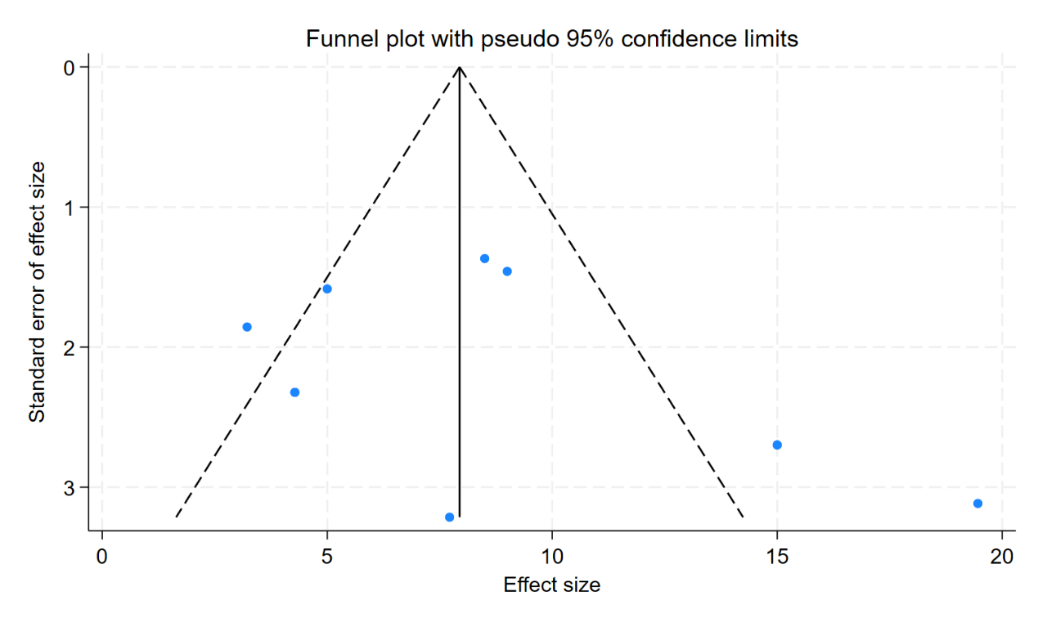
**

**Figure 30** Trim-and-fill plot of included studies on age for AAV-ILD.


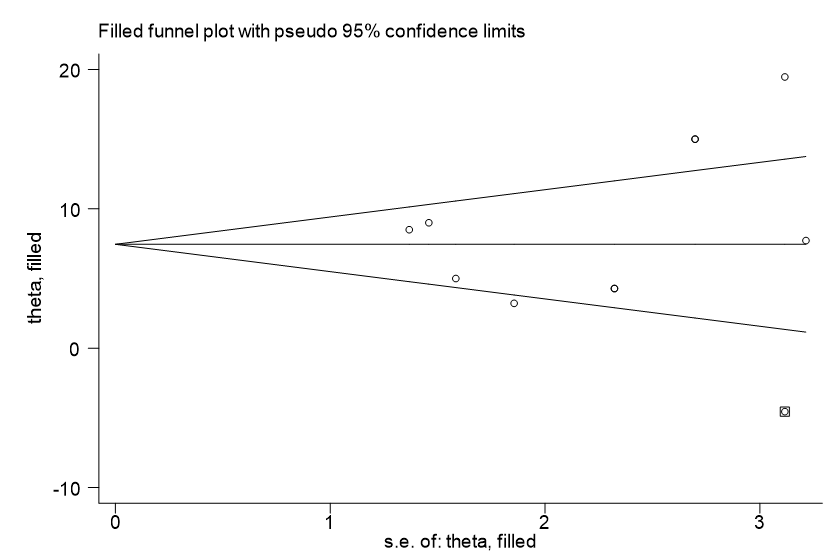


**Supplement E** Sensitivity analysis of included studies on risk factor for AAV-ILD.

**Figure E.1** Sensitivity analysis of included studies on age for AAV-ILD.

**Figure E.2** Sensitivity analysis of included studies on KL-6 for AAV-ILD.

**Figure E.3** Sensitivity analysis of included studies on ESR for AAV-ILD.

**Figure E.4** Sensitivity analysis of included studies on BVAS for AAV-ILD.

**Supplement F** Subgroup analysis with risk factor in AAV-ILD.

**Figure F.1** Subgroup analysis of the age and subgroup differences of study design.


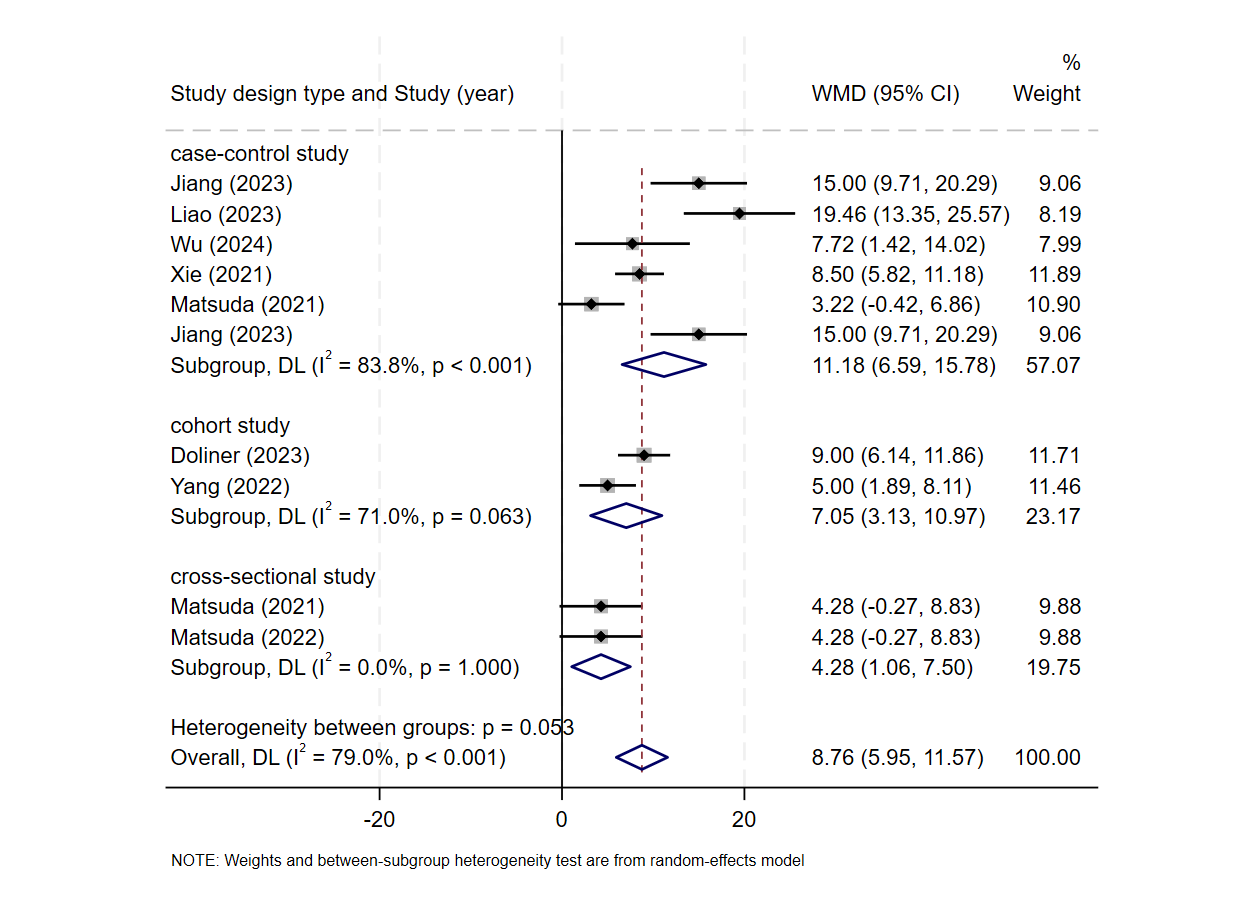


**Figure F.2** Subgroup analysis of the KL-6 and subgroup differences of study design.


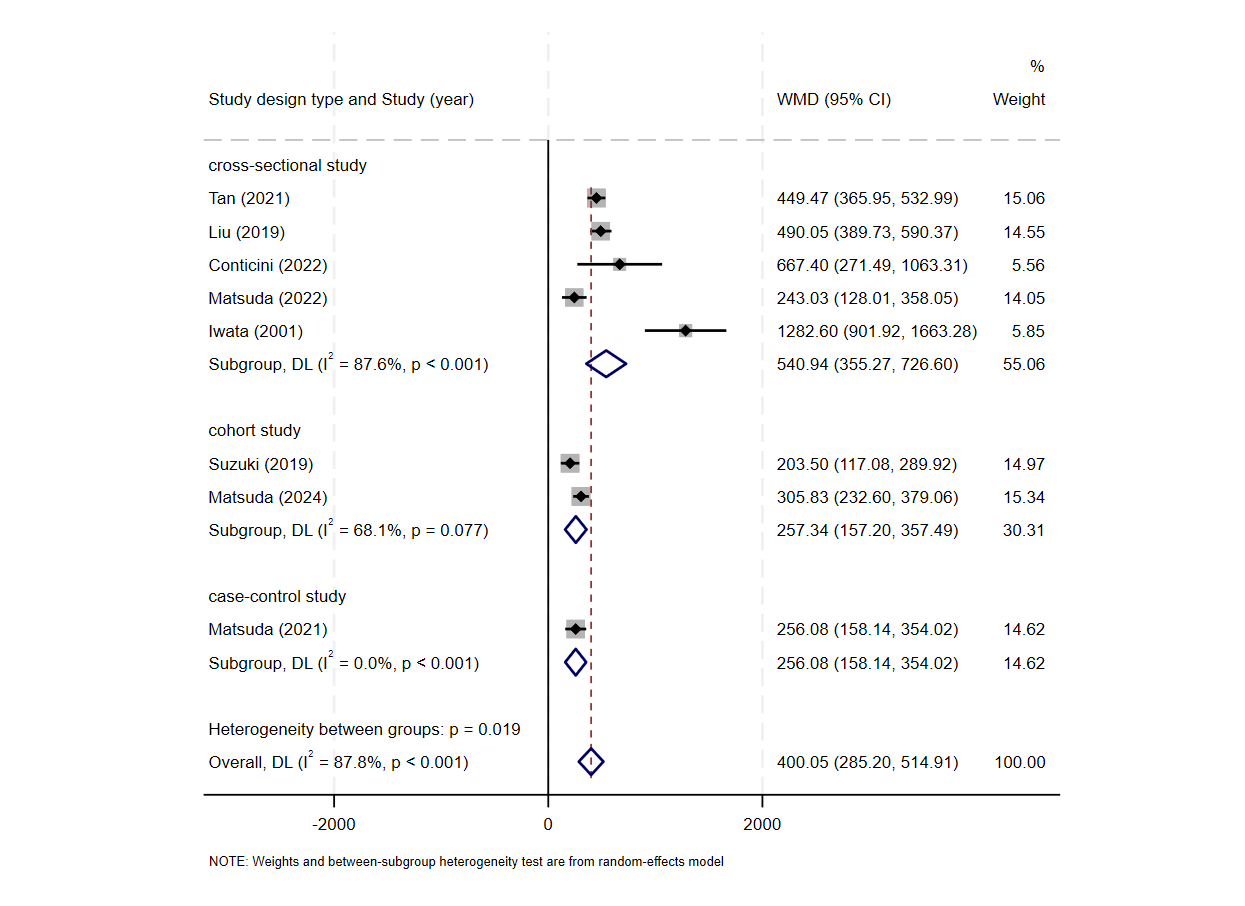


**Figure F.3** Subgroup analysis of the ESR and subgroup differences of study design
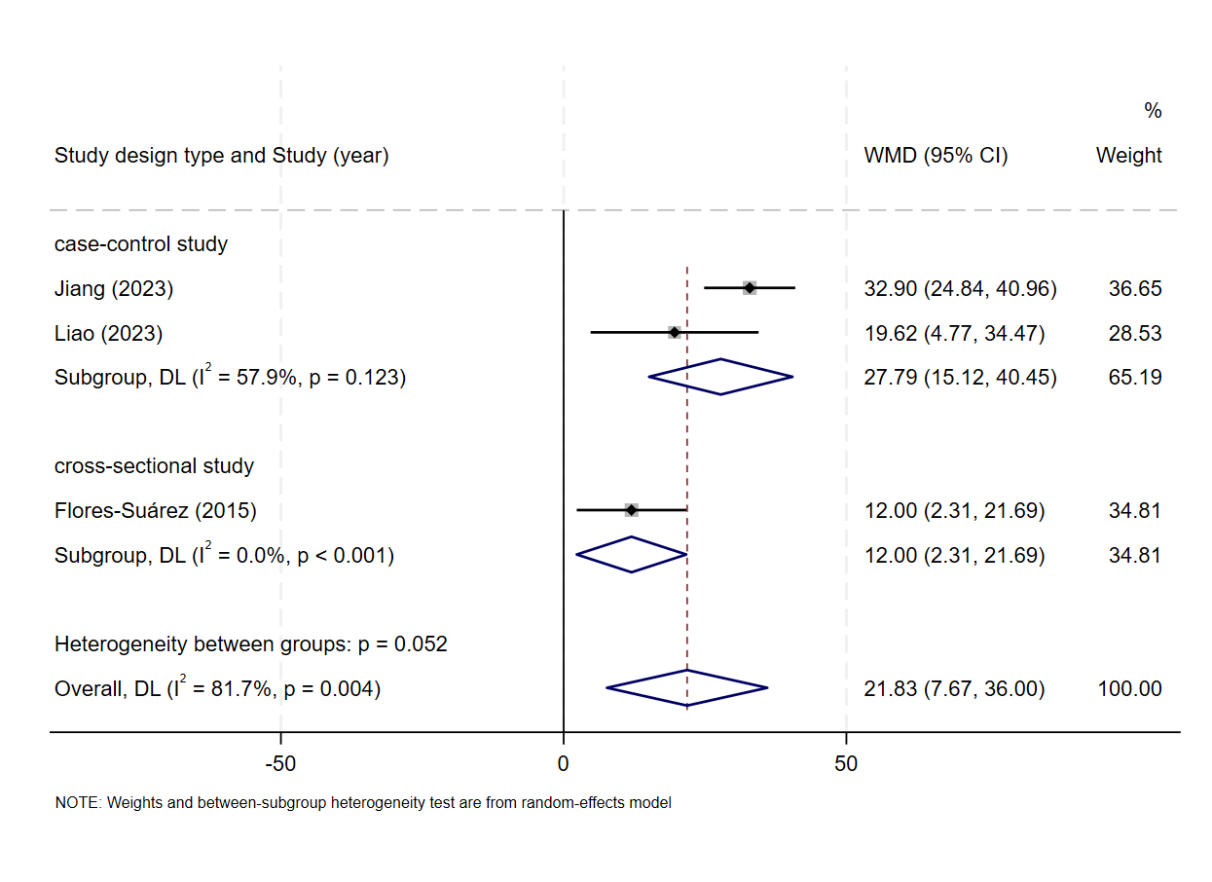


**Figure F.4** Subgroup analysis of the BVAS and subgroup differences of study design.


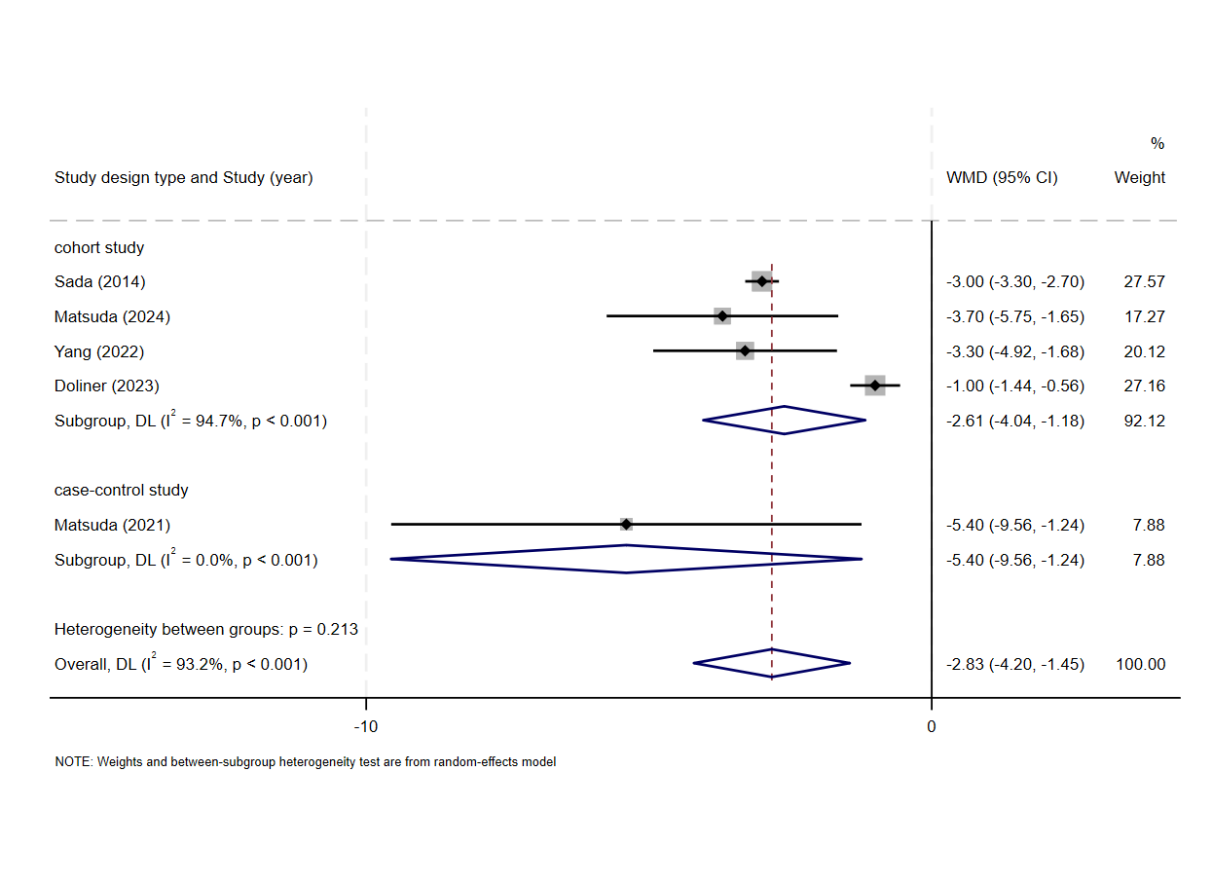


**Figure F.5** Subgroup analysis of the age and subgroup differences of sample.

**
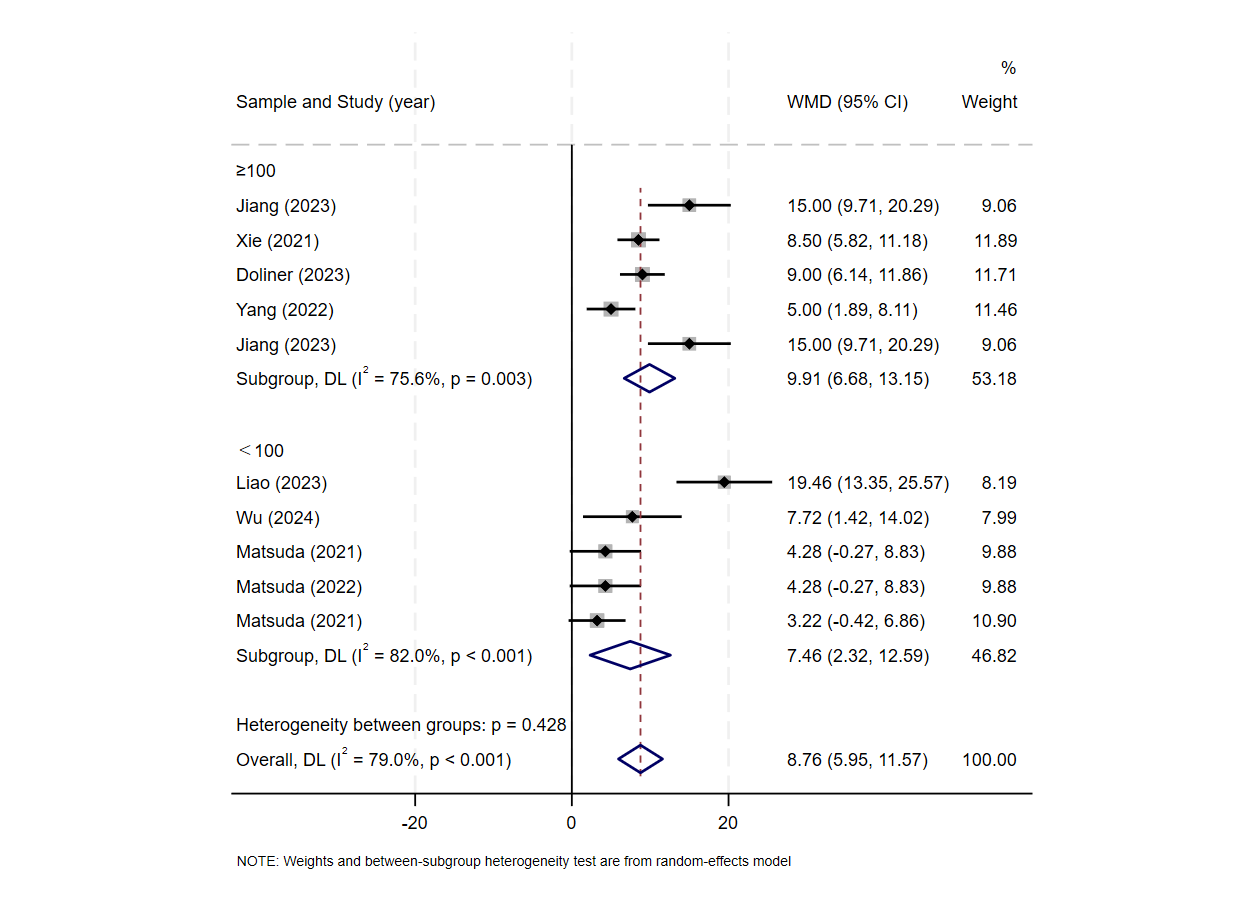
**

**Figure F.6** Subgroup analysis of the KL-6 and subgroup differences of sample.


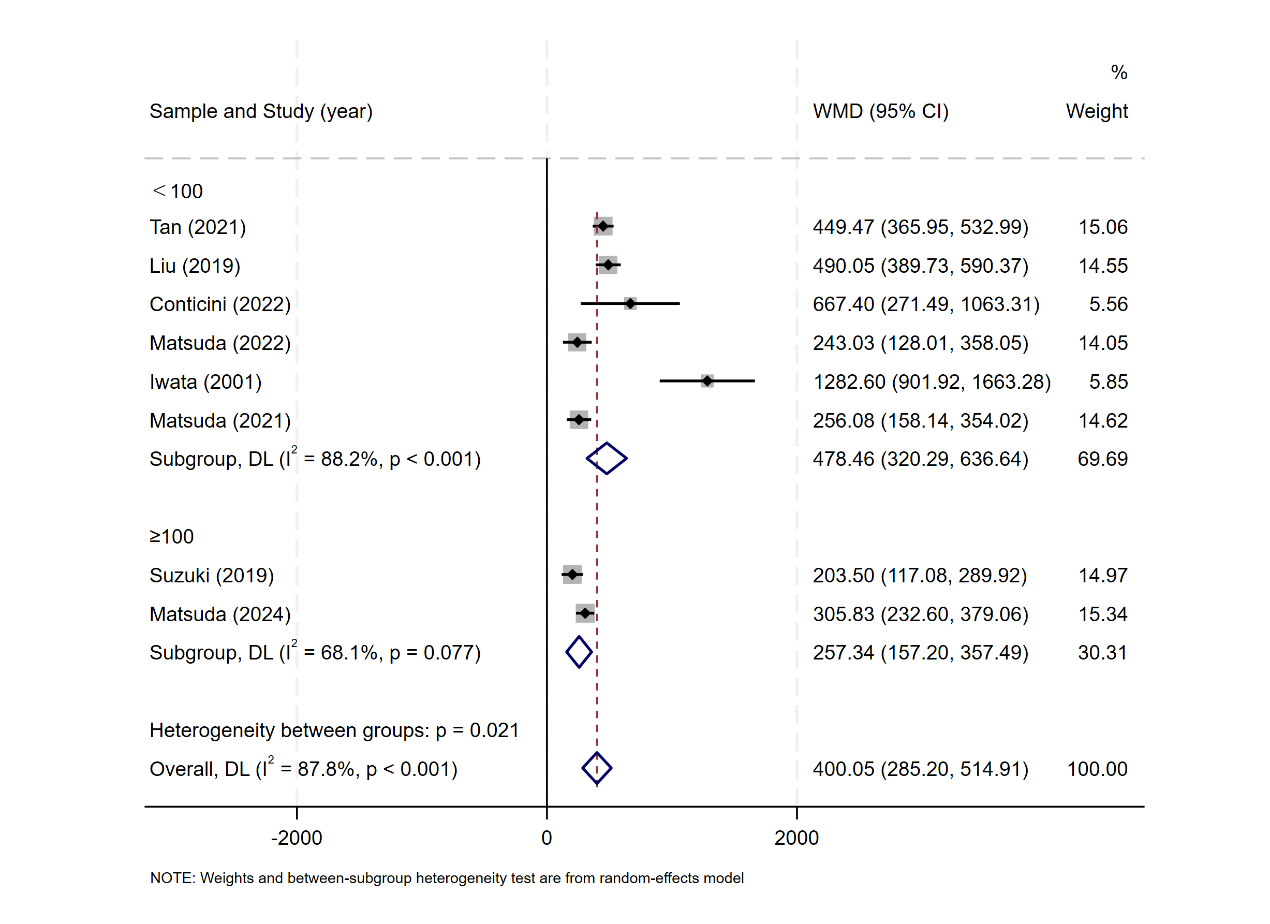


**Figure F.7** Subgroup analysis of the BVAS and subgroup differences of sample.


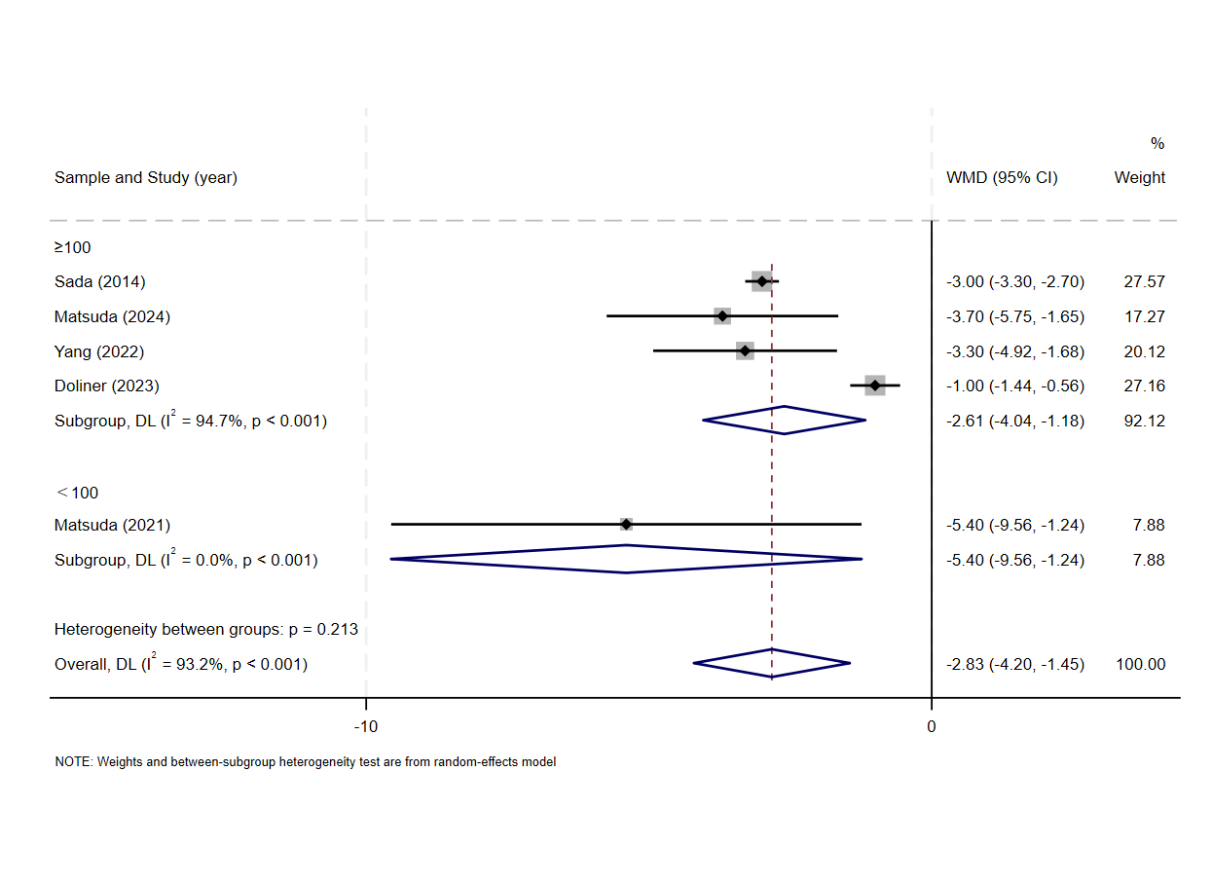


**Figure F.8** Subgroup analysis of the age and subgroup differences of Country.


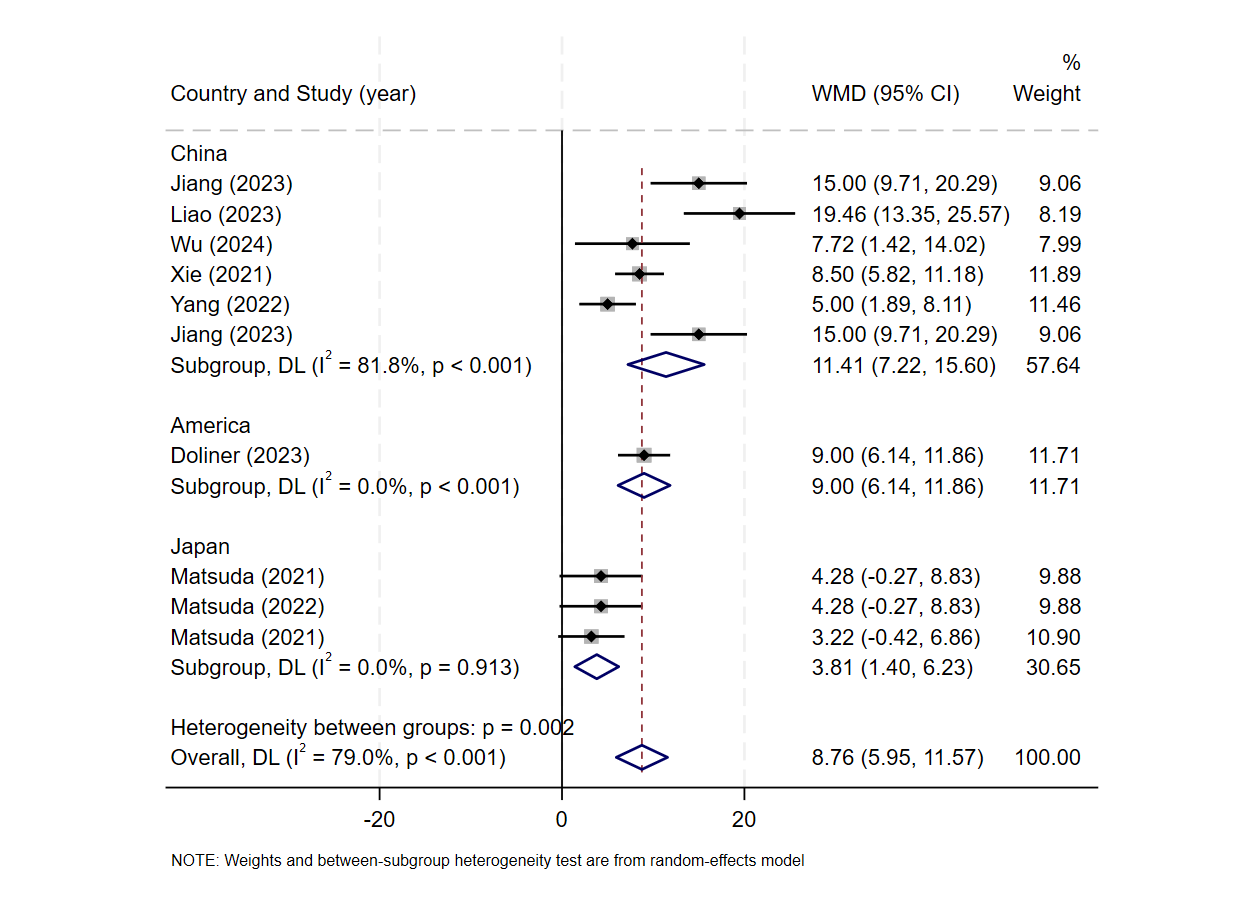


**Figure F.9** Subgroup analysis of the KL-6 and subgroup differences of Country.

**
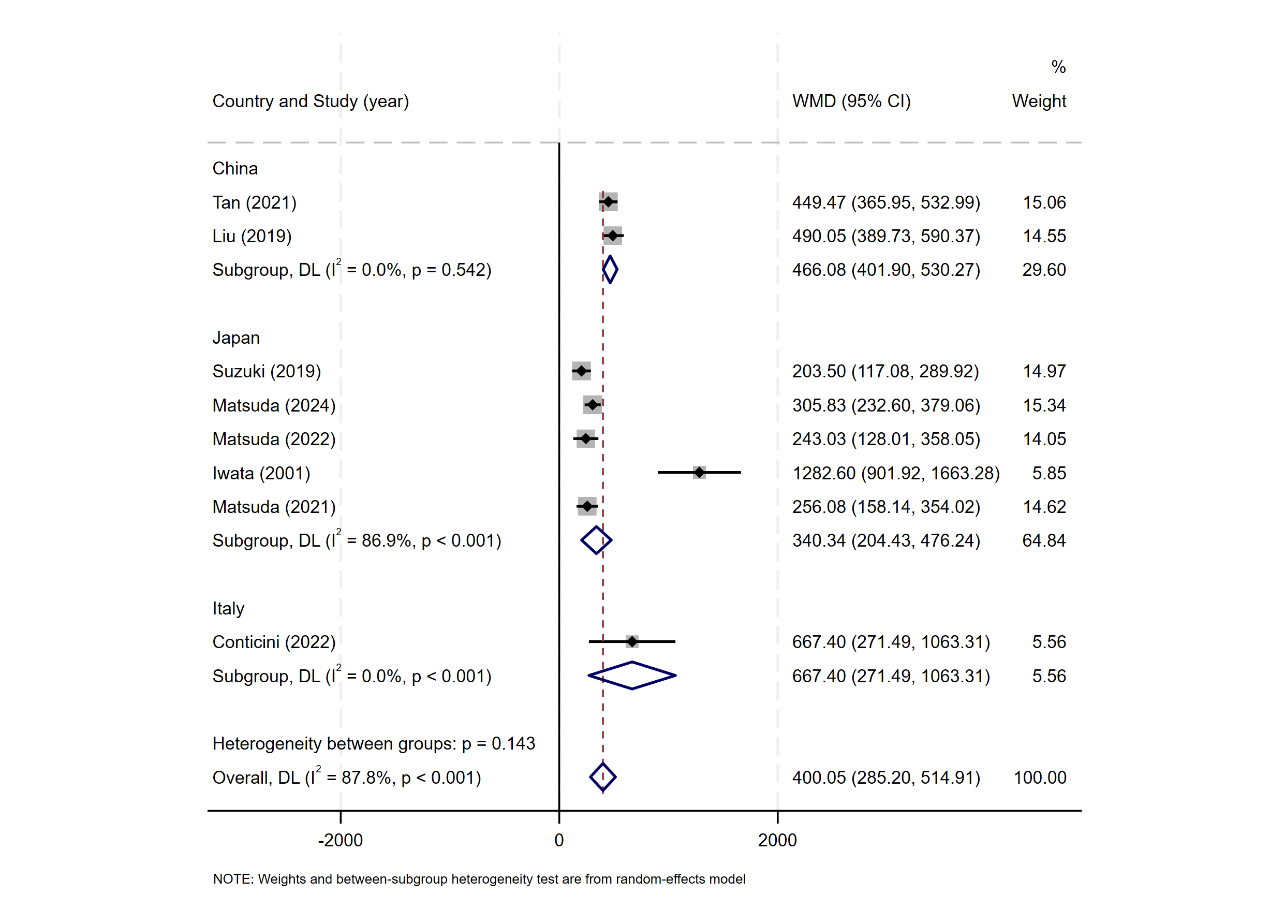
**

**Figure F.10** Subgroup analysis of the ESR and subgroup differences of Country.

**
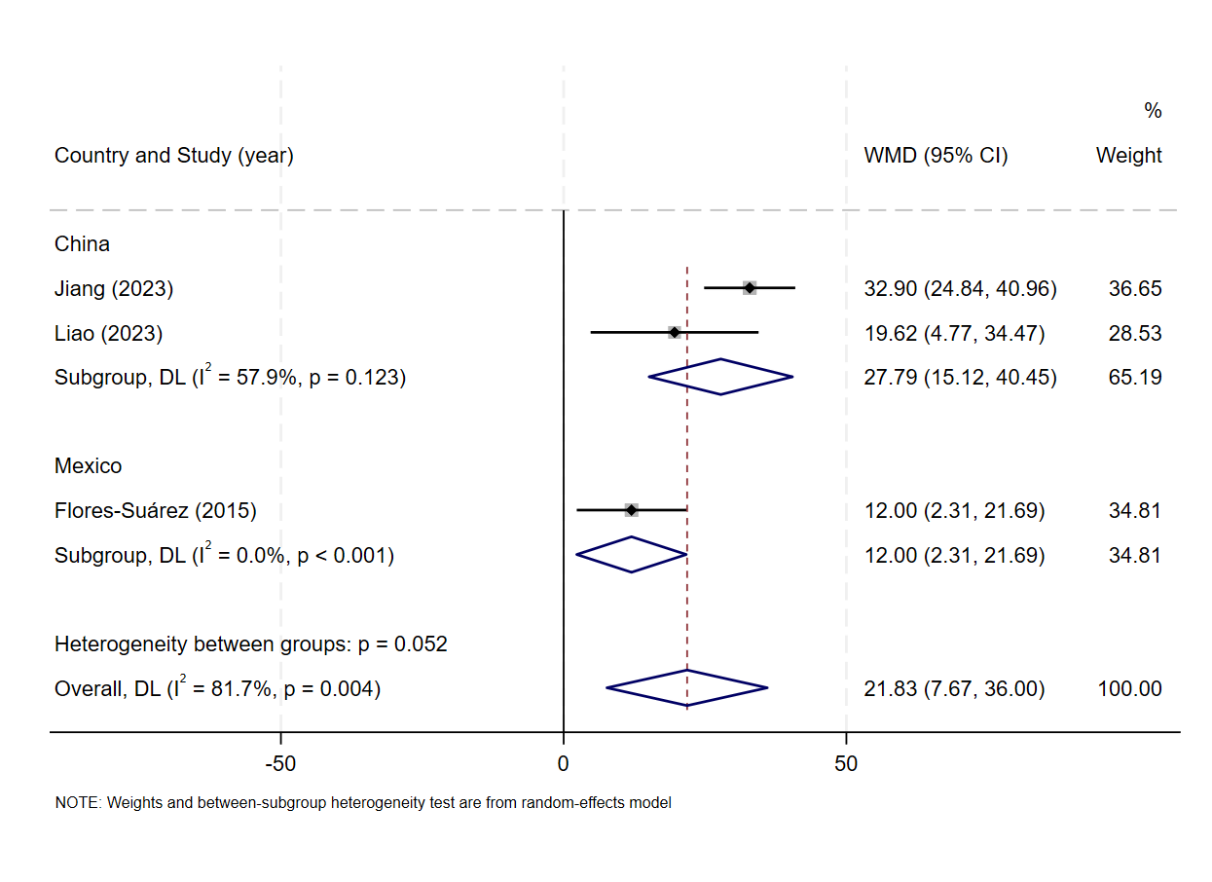
**

**Figure F.11** Subgroup analysis of the BVAS and subgroup differences of Country.

**
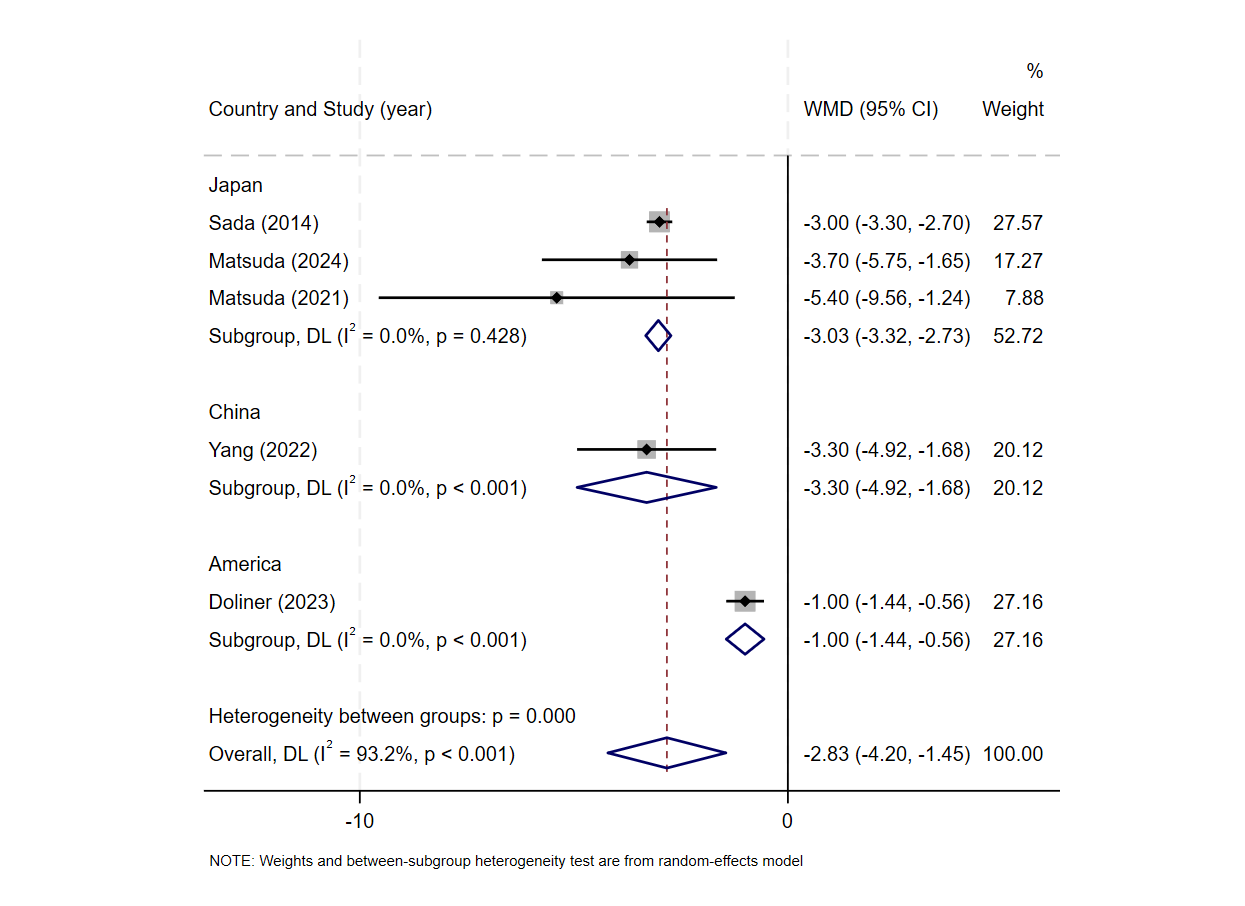
**

**Figure F.12** Subgroup analysis of the age and subgroup differences of follow-up (time).

**
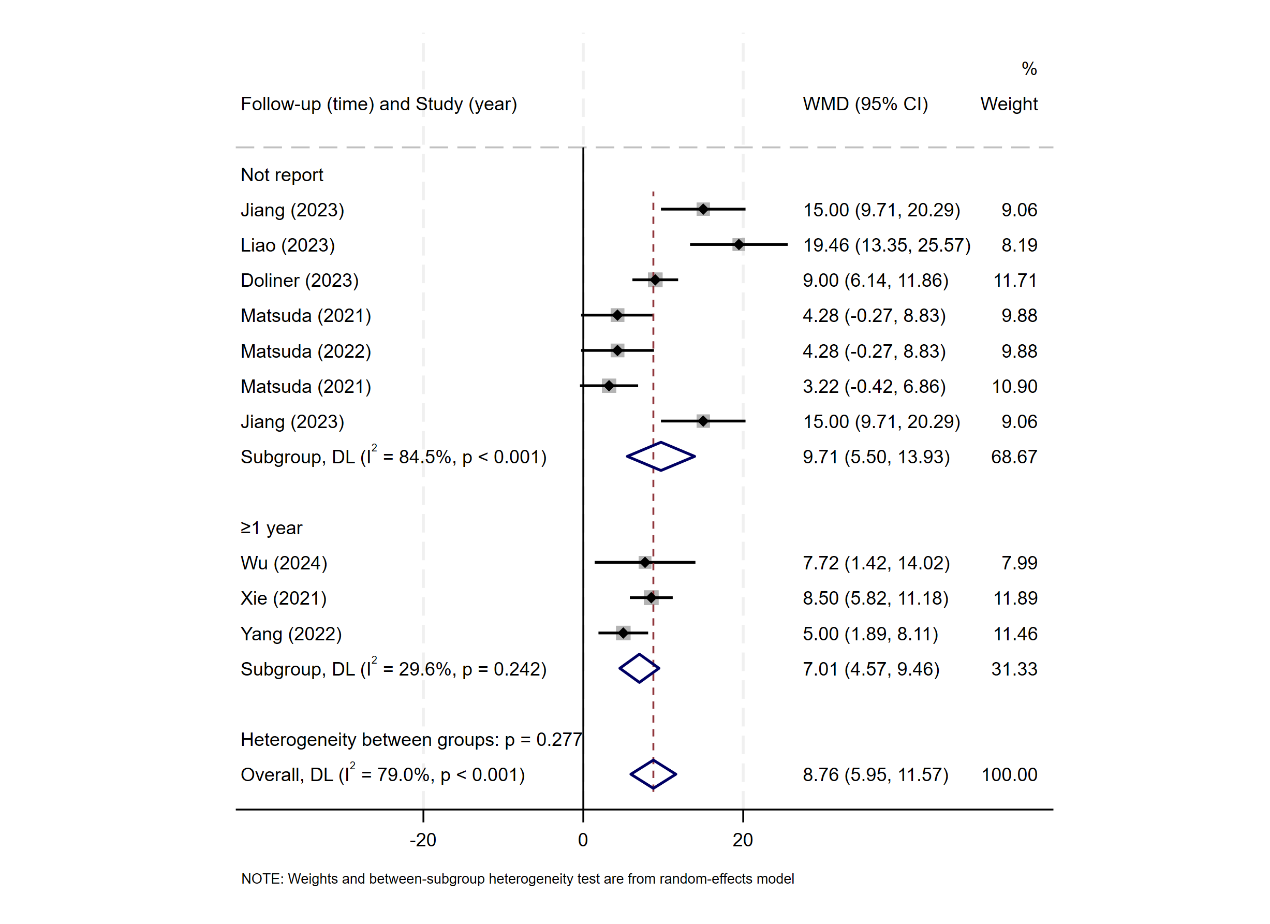
**

**Figure F.13** Subgroup analysis of the BVAS and subgroup differences of follow-up (time).

**
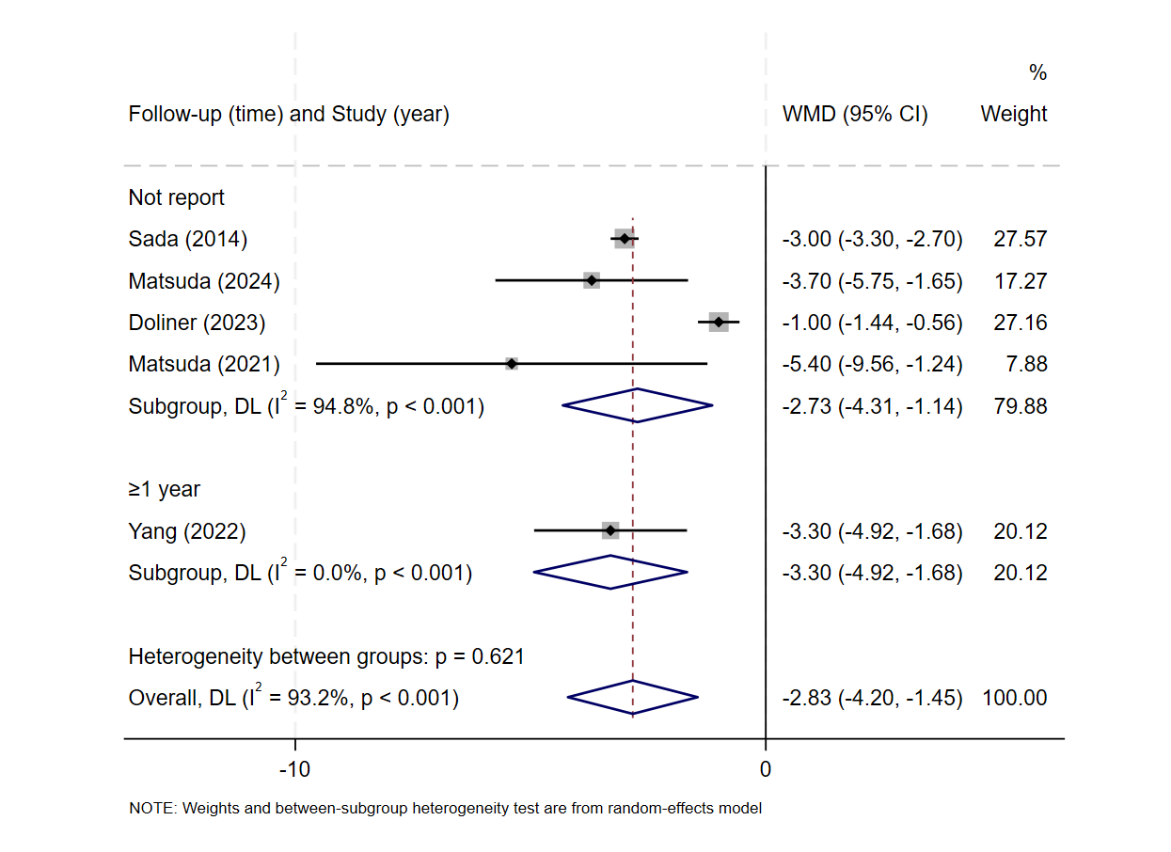
**

**Figure F.14** Subgroup analysis of the age and subgroup differences of type of AAV.

**
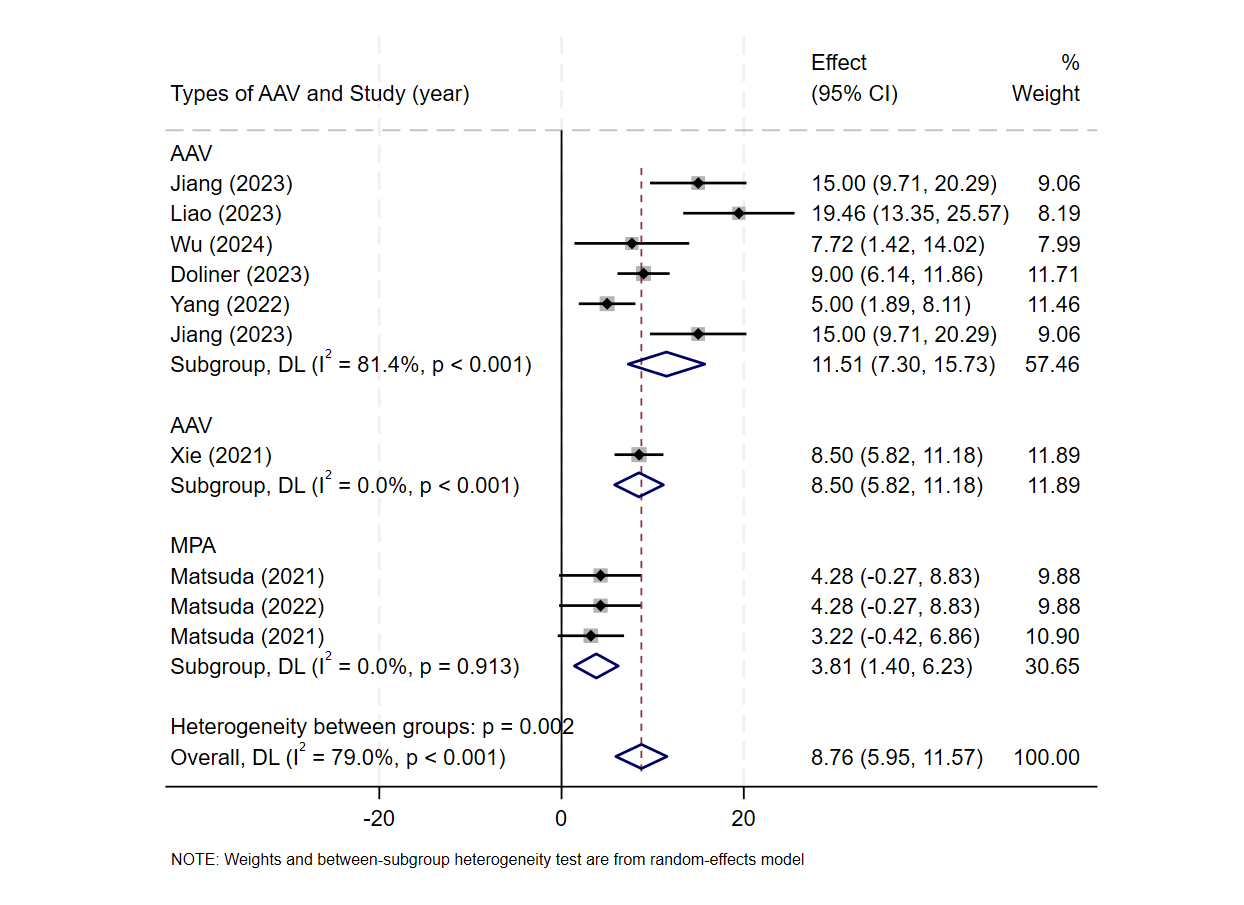
**

**Figure F.15** Subgroup analysis of the KL-6 and subgroup differences of type of AAV.


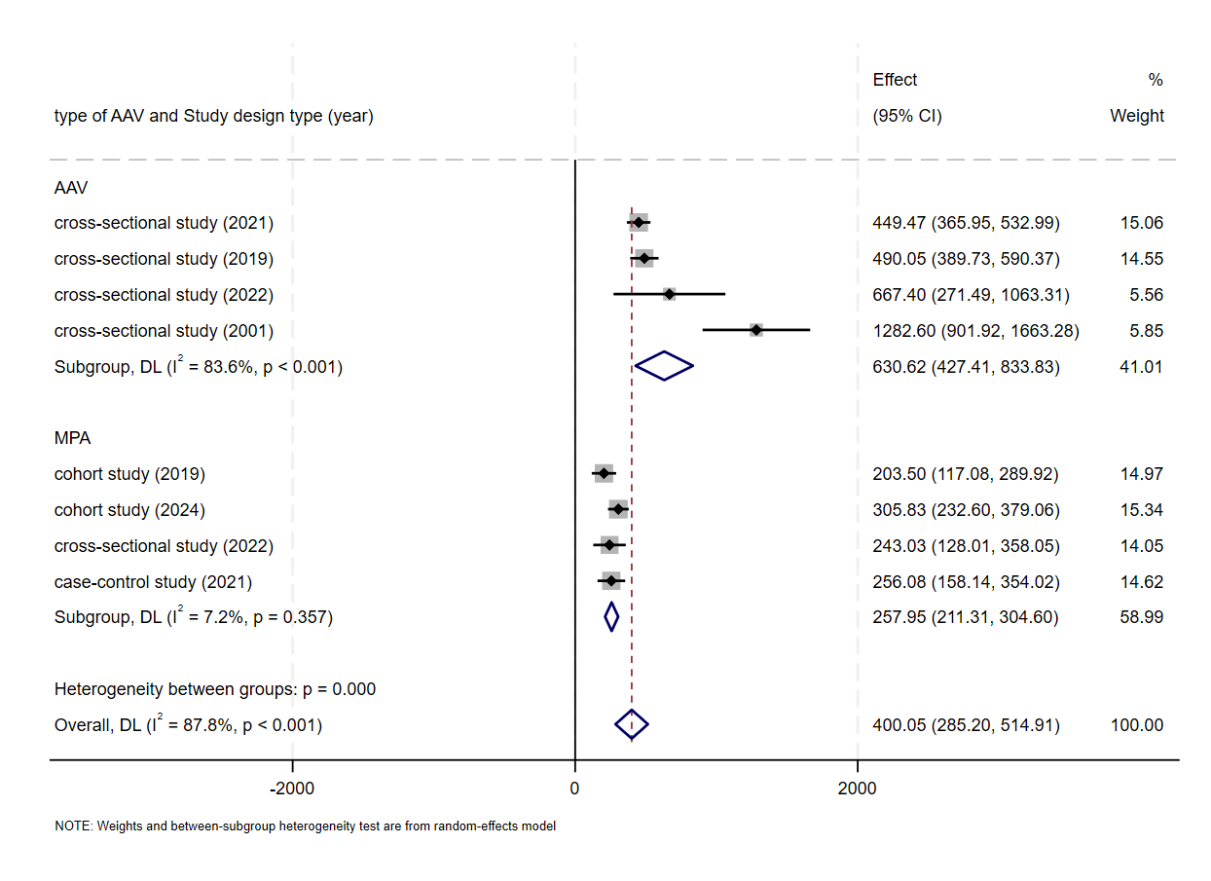


**Figure F.16** Subgroup analysis of the BVAS and subgroup differences of type of AAV.

**
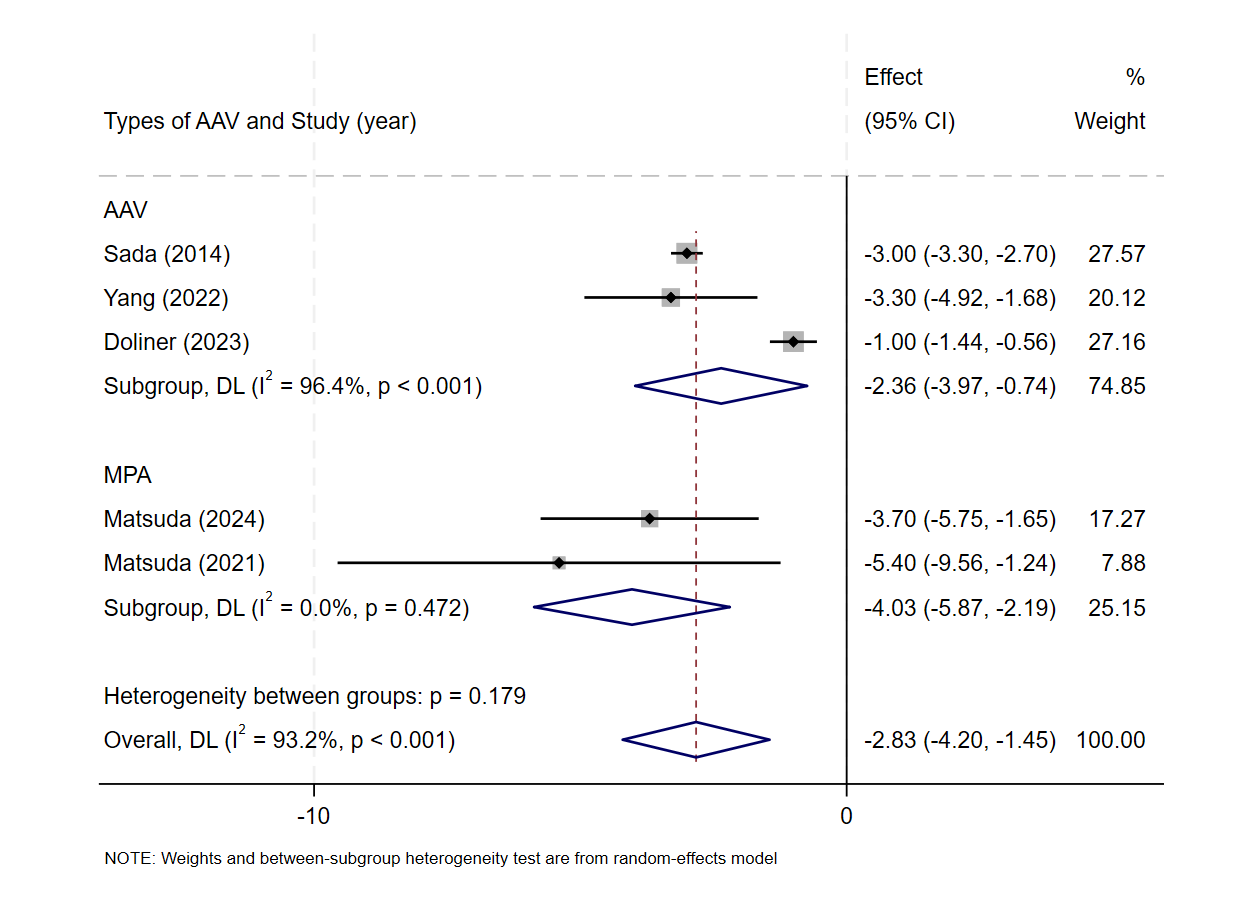
**

**Figure F.17** Subgroup analysis of theKL-6 and subgroup differences of Quality score.


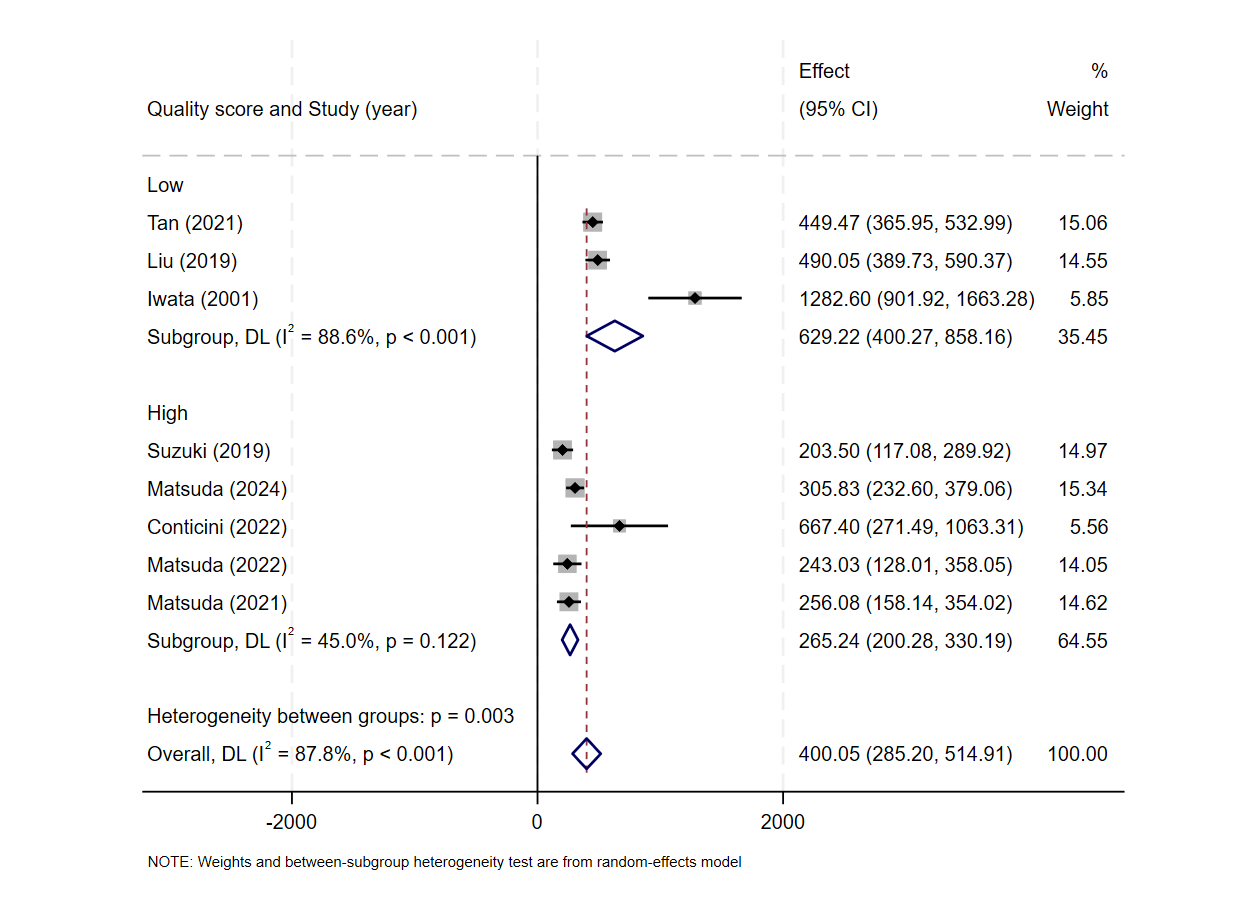

Supplement: Supplementary file 1 [file mmc1.docx]
